# Strong Effects of Temperature on the Early Life Stages of a Cold Stenothermal Fish Species, Brown Trout (*Salmo trutta *L.)

**DOI:** 10.1371/journal.pone.0155487

**Published:** 2016-05-12

**Authors:** Emilie Réalis-Doyelle, Alain Pasquet, Daniel De Charleroy, Pascal Fontaine, Fabrice Teletchea

**Affiliations:** 1 University of Lorraine, UR AFPA, USC INRA 340, Vandoeuvre-lès-Nancy, F-54506, France; 2 CNRS (National Center for Scientific Research) Délégation Régionale Centre Est. 17 rue Notre Dame des Pauvres, F -54500 Vandoeuvre-lès-Nancy, France; 3 Instituut voor Natuur- (INBO), Dwersbos 28, 1630 Linkebeek, Belgium; James Cook University, AUSTRALIA

## Abstract

Temperature is the main abiotic factor that influences the life cycle of poikilotherms. The present study investigated the thermal tolerance and phenotypic plasticity of several parameters (development time, morphometric measures, bioenergetics) for both embryos and fry of a cold stenothermal fish species, brown trout (*Salmo trutta* L.) in order to allow for a holistic evaluation of the potential effects of temperature. Five temperatures (4°C, 6°C, 8°C, 10°C, and 12°C) were tested, and the effects of temperature were analyzed at three stages: hatching, emergence, and first food intake. A mean of 5,440 (S.E. ± 573) eggs, coming from seven females and seven males (seven families) captured close to Linkebeek (Belgium), were used for each temperature. Maximum survival of well-formed fry at first food intake and better use of energy budget were found at 6°C and 8°C, temperatures at which the possible contribution to the next generation should therefore be greatest. At 12°C, the experimental population fell dramatically (0.9% survival rate for well-formed fry at first food intake), and fry had almost no yolk sac at first food intake. The present results on survival at 12°C are in accordance with predictions of a sharp decrease in brown trout numbers in France over the coming decades according to climate change projections (1°C to 5°C temperature rise by 2100 for France). At 10°C, there was also a lower survival rate (55.4% at first food intake). At 4°C, the survival rate was high (76.4% at first food intake), but the deformity rate was much higher (22% at first food intake) than at 6°C, 8°C, and 10°C. The energetic budget showed that at the two extreme temperatures (4°C and 12°C) there was less energy left in the yolk sac at first food intake, suggesting a limited ability to survive starvation.

## Introduction

Climate change is one of the main anthropogenic perturbations, in addition to destruction of habitats, introduction of alien species, pollution, and overexploitation, that have contributed to the erosion of biodiversity over the past century [1, 2]. Mean global temperatures have increased by 0.85°C since 1850 [3], which have partly contributed to the changes in fish fauna observed both in marine [4–6] and freshwater ecosystems [1, 2, 7–9]. According to the latest report of the Intergovernmental Panel on Climate Change [3], the average temperatures on the Earth’s surface could rise between 0.5°C and 5.5°C by the end of this century (2100) [3] with strong local variations. This warming trend could continue to affect fish populations, as most fish species have no physiological ability to regulate their body temperature [10, 11].

Freshwater fish species could be even more vulnerable to global climate change as they have limited dispersal ability within hydrographic networks in which they currently live [12, 13]. In this context, an important scientific issue is to predict how fish populations will cope with future temperature changes [14]. Buisson et al. [12] conducted one of the few studies on freshwater fish in Europe. They used correlative models that first linked the present-day species distribution to a set of environmental and climate variables. Subsequently, application of future climate change scenarios allowed predictions of future potentially suitable habitats for the species [13]. They concluded that the effects of climate change could be rather positive, except for cold-water species [12, 14]. Growing interest has emerged about the variability of the predicted effects from such models due to methodological decisions [12–15]. For example, the sampling method could lead to errors when evaluating the presence and abundance of species due to the migration of adult fish during the year [15]. Consequently, the absence of a given species in a sample does not necessarily imply that this site is not suitable for this species [15]. In addition, the modeling of present-day distribution is, most often, based only on adults, thus not taking into account the environmental requirements of the entire life cycle such as for embryos or larvae [15, 16]. Finally, several biological processes are not included in the modeling approach such as biotic interactions, dispersal ability, and potential colonization of new environments [15]. Other modeling methods have also been recently developed that aim to combine climatic requirements, population dynamics, and dispersal ability [15–16]. Yet, such models require a large amount of knowledge about population dynamics and processes involved in dispersal of the studied species, and as such have only been applied to a limited number of well-known species, such as the Allis shad (*Alosa alosa*) [16] or the Atlantic salmon (*Salmo salar*) [17].

Complementary to these modeling approaches, a possible way to improve both our understanding of the present geographical distribution of freshwater fish and our ability to interpret or model changes in fish communities, is to use the knowledge acquired on the thermal tolerance in fish by field or laboratory experiments [18]. Such information could be useful to refine present-day realized niche based on absence-presence records [12] and to evaluate whether species are today already able to cope with temperature changes. Yet, such knowledge, even for European freshwater species, is relatively old, fragmentary, and difficult to access [18, 19]. Most often, the available information has not been updated, confirmed, or refuted by recent works [19]. Therefore, new data obtained either through field studies coupling temperatures and the presence of fish, or more experimental designs conducted in laboratory on boundary thermal tolerance are required [18]. It seems appropriate to first focus on embryos and larvae, because these early life stages are key for further population recruitment, and are very sensitive to temperature conditions [18, 19].

In the present study, we chose to focus on brown trout (*Salmo trutta* L.) because its catch has dramatically declined in several parts of Europe (50% over the last 15 years [20]), such as in Switzerland and France, probably due to increasing water temperatures [20, 21]. According to projections, its distribution area could be reduced by 76% in France by the end of the century [12, 13] for which projected changes in temperature will be similar to the global average [3]. This species inhabits cold streams, rivers, and lakes [22]. Spawning occurs most often between October and December at around 8°C in rivers and streams with swift current [22, 23]. Few experimental laboratory studies have been performed on the thermal tolerance of early life stages of brown trout. They found that the maximum survival rate of fry was obtained between 8°C and 10°C, and that the lower lethal temperature was close to 1°C, while the upper limit was between 14°C and 16°C [24, 25]. Furthermore, deformity rates increased below 3°C and above 11°C [25, 26]. Previous studies also showed that an increase in temperature could induce an earlier development and a smaller size of hatching fry [24, 25]. Finally, the development time from fertilization to first food intake decreased sharply when temperature increased [27, 28], requiring 180 days at 4°C compared to only 60 days post fertilization at 14°C [24].

The present study had two main goals: (i) experimentally assess the thermal tolerance of early life stages of brown trout and the phenotypic plasticity of various traits, such as the size (and other morphometrics) at hatching, emergence, and first food intake, developmental duration of successive stages, and consumption of energetic reserves (yolk sac, tissue, and whole fish) in order to have a holistic evaluation of the potential effects of temperature, and (ii) based on these new results discuss previous experimental data and projections in order to provide more realistic forecasts for both policy makers and aquatic biodiversity managers.

## Materials and Methods

### Animals and experimental conditions

Fish were handled in accordance with national and international guidelines for the protection and welfare of animals used for scientific purposes (Directive 2010/63/EU). Eggs were obtained in December 2013, from wild broodstock of trout kept in earthen ponds at the Research Institute for Nature and Forest (INBO) located near Brussels. The temperature of the earthen ponds was 8.0°C (S.E. ± 0.2°C) and the oxygen concentration was 9.0 mg L^-1^. Seven females (mean mass: 2.50 ± 0.36 kg, mean length: 58.0 ± 3.9 cm) and seven males (mean mass: 3.18 ± 0.83 kg, mean length: 61.0 ± 4.7 cm) were used. The average age of broodstock was four years. Oocytes were obtained after stripping, and one female was fertilized by one male to obtain seven families. Then, eggs from each family were transported in separate plastic bags with water supplied from the stocking earthen ponds and put into polystyrene boxes to maintain the temperature close to 8°C. Overall, a mean of 4,170 ± 215 eggs per female were collected and transferred to the University of Lorraine, in Nancy (France), the same day.

Five similar recirculating water systems or hatcheries (110 × 64 × 186 cm) were used. Each hatchery had a flow rate of 4 m^3^ h^-1^, and water was U.V.-sterilized. Each hatchery contained eight separate racks (45 × 7 × 12 cm). The whole experiment was performed in a controlled temperature room at 15°C. The photoperiod was natural, with daylight hours increasing from December to the end of the experiment in April. The temperature of water was checked daily (precision of the probe is 0.1°C) in each hatchery, as well as dissolved oxygen, which remained above 9 mg L^-1^. Water quality (ammonia, nitrite and pH) was checked weekly. Total ammonia and nitrite concentrations in each tank were kept below 0.05 and 0.01 mg L^-1^, respectively, and pH remained at 8.0 ± 0.5. Eggs of the seven different families were mixed and distributed within each of the eight racks of the five hatcheries. After one day, the temperature was modified with a 1°C step per hour to reach the desired tested temperatures [29]. The five temperatures (4°C, 6°C, 8°C, 10°C, and 12°C) were chosen according to climate predictions for France.

#### Biological stages

Four biological stages (fertilization, hatching, emergence, and first food intake) were considered and three time periods were defined: P1: from fertilization to hatching, P2: from hatching to emergence, and P3: from emergence to first food intake (beginning of exogenous feeding). Hatching (H) was defined as the time when at least 50% of the fry left the egg shell, emergence (EM) as the time when at least 50% of the fry rose up to swim in the water column, and first food intake (FFO) as the time when at least 50% of the fry started to feed exogenously. We chose 50% because it is most commonly used in the literature [24, 25]. Feed specifically developed for trout (Neo supra S, AL1; Le Gouessant) was given just after emergence twice daily (9.00 a.m. and 2.00 p.m.) [30, 31].

### Parameters studied

#### Survival rate

Dead embryos and fry were removed every day to avoid water pollution. Dead embryos were easily identified as they were white and opaque [32]. Dead individuals (embryos or fry) were counted every day to calculate the daily mortality rate (DMR, in percentage) over a period of time, as follows: [DMR = ((Number of dead individuals cumulated over a period of time / Number of days within this period of time) × 100 / Total number of individuals]. Based on these data, the mortality rate was obtained and we then calculated the survival rate (SR) for the entire experiment (from fertilization to first food intake) and at each stage as follows: [SR = 100 - (Number of dead individuals cumulated over a period of time × 100) / Number of individuals at the beginning of the studied period of time]. The daily survival rate was also calculated for each time period by taking into account its duration. The eggs (dead within 24 h after fertilization) were not taken into account as dead but as unfertilized eggs; they were removed from the analysis.

#### Deformity rate

Fry were classified as either well-formed or not and scored as yes or no. This classification was performed on 128 live fry from each temperature group (n = 16 per rack) at the end of each period (3 times). All fry, which were then finally sampled for morphometric measures, were checked for malformations. These fry were then used for the analysis of bio-energy. Those that were malformed had visible defects, including malformations of the skeleton, head and yolk sac [33, 34]. The deformity rate (DF) of live fry was calculated as follows: [DF = Number of fry malformed × 100 / 128 live sampled fry]. Then, by combining the survival and deformity rates of live fry, we calculated the survival rate of well-formed fry (PS) as follows: [PS = Survival rate × (100—Deformity rate) / 100]. PS provided us with an indication of the quality of the fry population at a given stage.

#### Duration of the developmental stages

The total duration of the experiment corresponded to the time between fertilization and observation of the first food intake (days), spanning to the endogenous feeding period. The relative time (RT) of each time period (P1, P2, and P3) was calculated as follows: [RT = Number of days between two consecutive stages × 100 / total duration of the endogenous feeding period].

#### Morphometric and morpho-anatomical parameters

The morphological parameters analyzed were selected based on Trabelsi [25, 35]. The morphometric measurements were performed on well-formed fry only among the 128 fry sampled per temperature and per stage. Fry were euthanized by an overdose of Ethyl 3-aminobenzoate methanosulfonate salt (MS-222, Tricaine). All measures were obtained with an Optika binocular microscope equipped with a Sony camera, with a precision of 0.01 mm. Three parameters were measured on the body of the fry: total length (TL) (mm), height of the myotome (HM) (mm), and eye diameter (ED) (mm), and two on the yolk sac: vertical and horizontal diameters (mm). The mean diameter of the yolk sac (YSD) was then calculated by averaging the two diameters [35].

#### Energetic value

The dry mass (0.01 mg) was obtained based on the analysis of 24 fry (n = 3 per rack) at three different stages: hatching, emergence, and first food intake. For each fry, the yolk sac and tissue were first separated [24], dried at 60°C for 72 hours, and then weighed using a Denver Instrument S-114 balance (with a precision of 0.01 mg). This allowed obtaining the separate mass of yolk and tissue (MY and MT, respectively), as well as the energetic values of each. The total energetic value of fry was obtained by adding the values of the yolk and tissue. Elemental analyses were performed using a Flash EA 1112 elemental analyzer (Thermo Finnigan, 2003) at the Laboratory of Physical Measures (LMP) at the University of Montpellier II (France). Each sample (25 mg) was divided in two parts: one for carbon (*C*), hydrogen (*H*), nitrogen (*N*), and sulfur (*S*) analyses and the other to determine the total energetic content. Each sample was analyzed in triplicate. The oxygen fraction (*O*, % dry matter) was then computed as follows: [*O* = 100 - (*C* + *H* + *N* + *S*)]. Caloric values (J mg^-1^ dry mass) were computed from *C*, *H*, *O*, and *S* fractions (% dry matter). The following formula was used to calculate the caloric values: [*CV (*J mg^-1^*) =* 0.004184 × [88 *× % C* + 344 × *(*% *H–* 0.125 × % *O)* + 25 × % *S*] [36]. Tissue or yolk sac energetic value (EV, J ind.^-1^) was obtained by multiplying the caloric value (CV, J mg^-1^) by the dry mass (PS, mg): [EV (J ind.^-1^) = CV × PS], as described in [36].

### Statistical analyses

To test the normality of the distributions, a Kolmogorov–Smirnov test was used and the homogeneity of variances was tested using the Levene’s F test. Then, Generalized Linear Models (GLM) were used to determine differences in survival rate, daily mortality rate, deformity rate, survival of well-formed fry, total length of fry, and energy value. In all analyses, temperature and stage were the fixed factors and racks (nested within temperature) were considered as a random factor. The GLM were followed by a Tukey post hoc test to calculate pairwise differences between means. All parameters were expressed as mean ± S.E. Besides, for the survival curve, we used a Cox regression proportional hazard survival analysis. For development time, we used an exponential model. The effect of temperature on the percentage of time between two consecutive stages was tested with chi^2^. All results were considered significant at the level of *p* < 0.05.

A Principal Components Analysis (PCA) was used to investigate the relationships between the six morphometric parameters (ED: eye diameter; TL: total length; HM: height of the myotome; YSD: yolk sac diameter; MY: mass of the yolk sac; and MT: mass of the tissue), as well as the ordination of fry at each of the three studied stages (hatching, emergence, and first food intake). PCA was followed by a hierarchical cluster analysis to identify groups of fry sharing similar morphometric parameters. Then, a mean individual (calculated from the 16 fry sampled per rack, therefore 8 individuals per temperature) was then plotted for the first and second axes for the purpose of clarity. All statistical analyses were performed using Statistica software (version 10).

## Results

### Duration of the developmental stages

**Fig 1:**
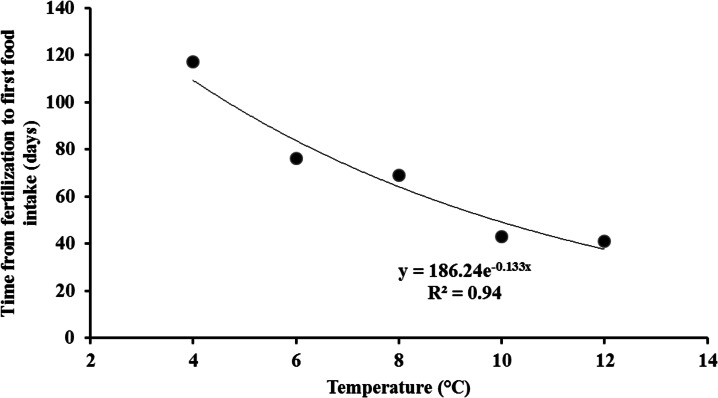
Effect of temperature on the development time from fertilization to first food intake (in days) for brown trout (*Salmo trutta*). Each point represents one value per temperature.

**Fig 2:**
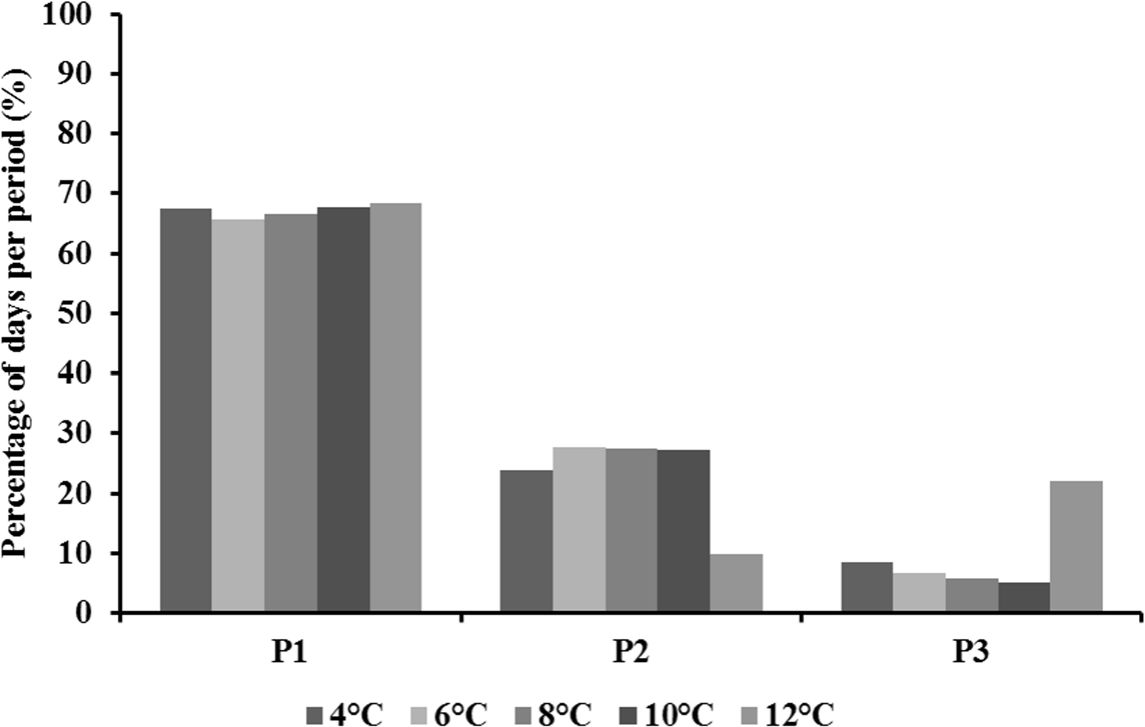
Effect of temperature on the percentage of days per time period for brown trout (*Salmo trutta*). P1: from fertilization to hatching, P2: from hatching to emergence, and P3: from emergence to first food intake. Only one value was used per temperature.

The total duration from fertilization to first food intake was significantly different between temperatures (X^2^ = 196.3, *p* < 0.001). It decreased exponentially with increasing temperatures (Fig 1). The relative duration time of each stage varied among stages (X^2^ = 56.0, *p* < 0.001) but no interaction was observed between stages and temperatures (X^2^ = 11.7, *p* = 0.16). The relative duration of period P1 (incubation) was similar between temperatures (between 66% and 69%) (Fig 2). However, temperature had an effect on the two other periods (P2, P3); yet, this could not be tested statistically: P2 was the shortest and inversely P3 was the longest at 12°C, compared with the other temperatures (Fig 2).

### Influence of temperature on survival rate and deformities

#### Survival rate

**Fig 3:**
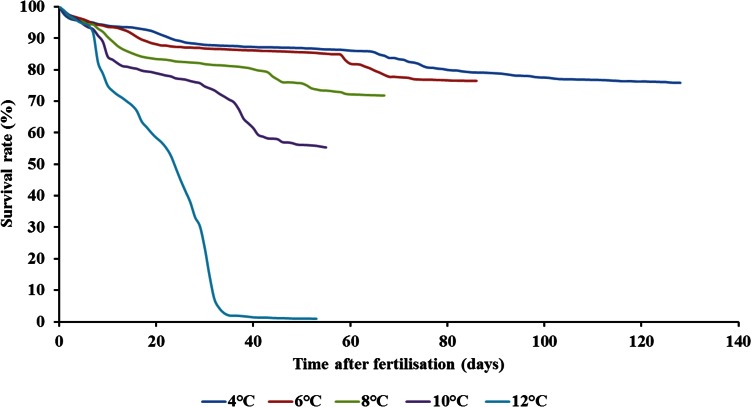
Effect of temperature on the survival rate (%) of brown trout (*Salmo trutta*) from fertilization to first food intake. Each line corresponds to the mean of dead individuals obtained for the 8 racks per day per temperature cumulated throughout the experiment.

During incubation, high peaks of mortality were observed at 6 to 20 days post fertilization depending on the temperature tested: at day 6 at 12°C (number of dead embryos for the entire hatchery, *n* = 595), at day 7 at 10°C (*n* = 213), at day 8 at 8°C (*n* = 83), at day 14 at 6°C (*n* = 49), and at day 20 at 4°C (*n* = 29) (S1 Table). The survival rates were significantly different between temperatures (GLM: *F*_1,4_ = 1221.9, *p* < 0.0001) and stages (GLM: *F*_1,2_ = 176.4, *p* < 0.0001), and were also influenced by the interaction between temperatures and stages (GLM: *F*_1,8_ = 41.7, *p* < 0.0001). There was a slight effect of racks (GLM: *F*_1,35_ = 2.31, *p* < 0.001). Besides, the Cox survival analysis showed that there was a global effect of temperature on mortality (X^2^ = 876, *p* < 0.0001) (Fig 3). Finally, the daily mortality rate (in percentage) during each of the three studied time periods (P1, P2, P3) was significantly different between temperatures (GLM: *F*_1,4_ = 482.2, *p* < 0.0001) and stages (GLM: *F*_1,2_ = 211.6, *p* < 0.0001), and was also influenced by the interaction between temperatures and stages (GLM: *F*_1,8_ = 57.4, *p* < 0.0001). There was no effect of racks (GLM: *F*_1,35_ = 1.0, *p* < 0.0001). The daily mortality rate was highest for both P1 and P2 at 12°C compared with the other temperatures (Table 1). Yet, no difference was found between temperatures for P3 (Table 1).

**Table 1:** Effect of temperature on the daily mortality rate (in percentage) of brown trout (*Salmo trutta*) during each of the three studied period. P1: from fertilization to hatching; P2: from hatching to emergence; P3: from emergence to first food intake. Mean (± S.D.), with n = 8 (racks). Values with the same superscript letter indicate no significant difference between temperatures within each period (Tukey, *p* < 0.05).

	P1	P2	P3
4°C	0.19 ± 0.07 ^a^	0.52 ± 0.19 ^a^	0.02 ± 10^−4 a^
6°C	0.31 ± 0.12 ^ab^	0.22 ± 0.10 ^ab^	0.04 ± 0.03 ^a^
8°C	0.50 ± 0.14 ^ac^	0.52 ± 0.66 ^ab^	0.06 ± 0.05 ^a^
10°C	0.70 ± 0.14 ^bc^	1.13 ± 0.32 ^c^	0.06 ± 0.08 ^a^
12°C	2.32 ± 0.24 ^d^	3.08 ± 0.45 ^d^	0.05 ± 0.02 ^a^

#### Deformity rate

**Fig 4:**
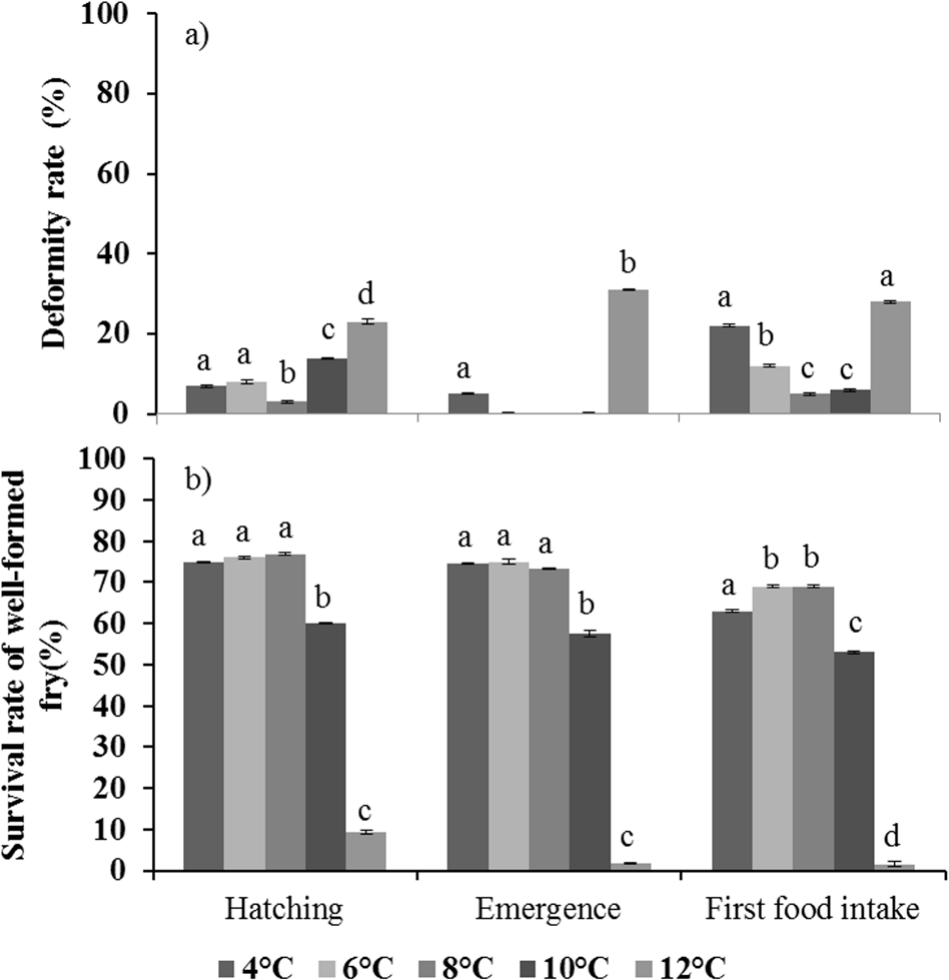
**Effect of temperature on a) the deformity rate (%), and b) survival rate of well-formed fry (%) at three different stages (hatching, emergence, and first food intake) for brown trout (*Salmo trutta*).** Mean (± S.D.), with n = 8 (racks). Values with the same superscript letter indicate no significant difference between temperatures for each of the three stages (Tukey, *p* < 0.05).

The deformity rate was significantly different between temperatures (GLM: *F*_1,4_ = 134.4, *p* < 0.0001) and stages (GLM: *F*_1,2_ = 44.9, *p* <0.0001), and was also influenced by the interaction between temperatures and stages (GLM: *F*_1,8_ = 27.7, *p* < 0.0001). There was no effect of racks (GLM: *F*_1,35_ = 0.8, *p* = 0.75). At hatching, the percentage of malformed fry was highest at 12°C and lowest at 8°C (Fig 4(A)). At both emergence and first food intake, the percentage was highest at the two extreme temperatures (Fig 4(A)).

#### Survival rate of well-formed fry

The survival rate of well-formed fry was significantly different between temperatures (GLM: *F*_1,4_ = 11070.3, *p* < 0.0001) and stages (GLM: *F*_1,2_ = 1244.7, *p* < 0.0001), and was influenced by the interaction between temperatures and stages (GLM: *F*_1,8_ = 535.5, *p* < 0.0001). There was no effect of racks (GLM: *F*_1,35_ = 1.0, *p* = 0.48) (Fig 4(B)). For the three stages, the survival rate of well-formed fry was lowest at 12°C, followed by 10°C (Fig 4(B)). At first food intake, the survival rate was also lower at 4°C than at 6°C and 8°C (Fig 4(A)).

### Morphometric and morpho-anatomical measurements

#### Total length

**Fig 5:**
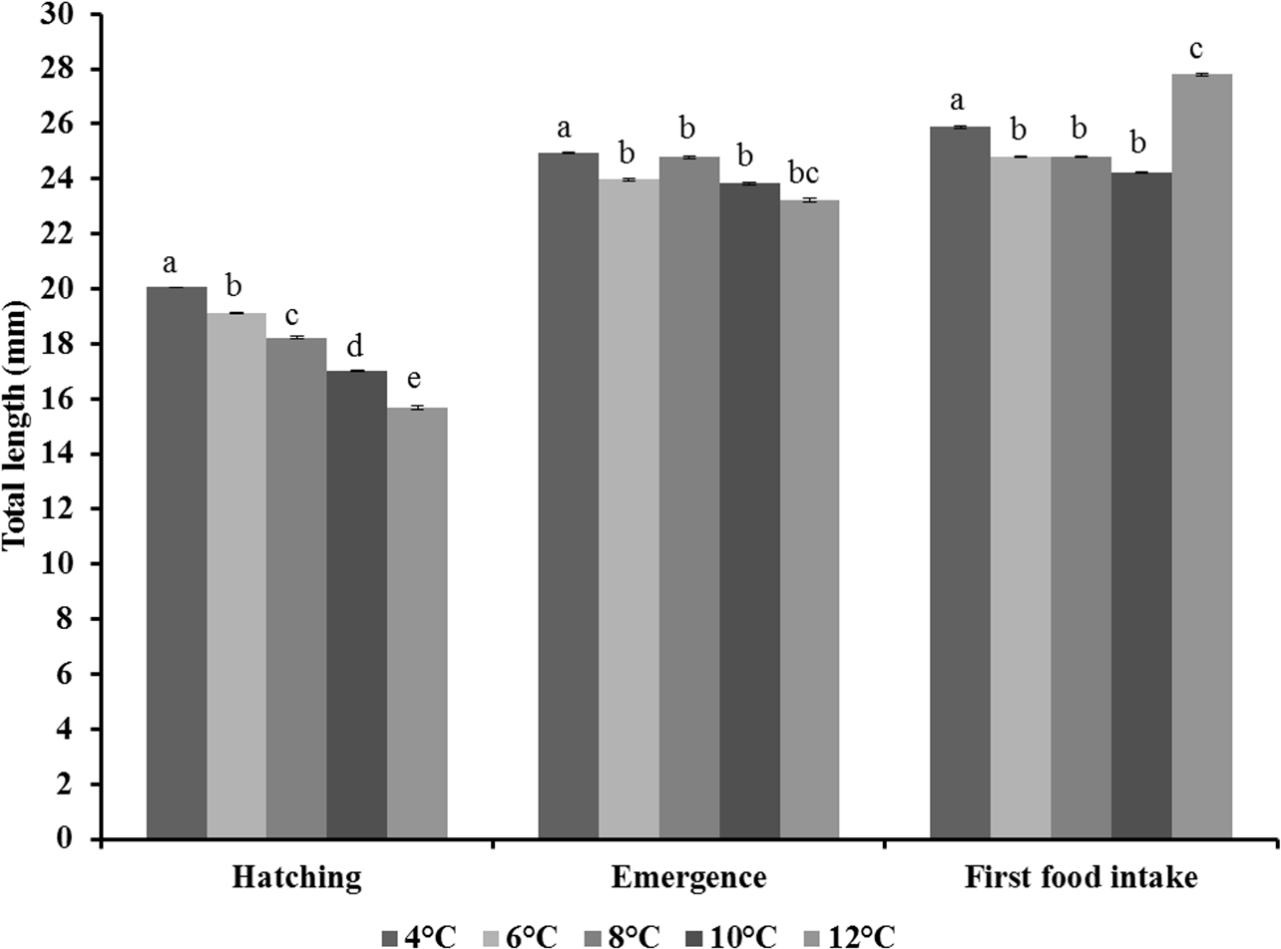
Effect of temperature on the total length of fry of brown trout (*Salmo trutta*) at hatching, emergence, and first food intake. Mean (± S.D.), with n = 8 (racks). Values with the same superscript letter indicate no significant difference between temperatures for each of the three stages (Tukey, *p* < 0.05).

The total length was significantly different between temperatures (GLM: *F*_1,4_ = 108.3, *p* < 0.0001) and stages (GLM: *F*_1,2_ = 4775.3, *p* < 0.0001), and was also influenced by the interaction between temperatures and stages (GLM: *F*_1,8_ = 90.8, *p* < 0.0001). There was no effect of racks (GLM: *F*_1,35_ = 1.1, *p* = 0.33). At hatching, the total length of fry was significantly reduced when temperature increased (Fig 5, S2 Table). At emergence, fry were longest at 4°C and of intermediate length at 6°C, 8°C, 10°C, and 12°C. At first food intake, they were shortest at 6°C, 8°C, and 10°C, of intermediate length at 4°C, and longest at 12°C (Fig 5, S2 Table).

#### Morpho-anatomical changes

**Fig 6:**
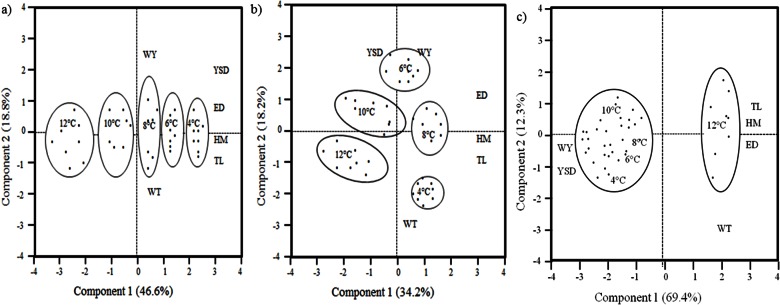
**Principal Components Analysis performed on six morphometric parameters measured on brown trout (S*almo trutta*) fry, a) at hatching, b) at emergence, and c) at first food intake.** TL = total length, HM = height of the myotome, ED = eye diameter, MT = mass of tissue, MY = mass of yolk sac, and YSD = yolk sac diameter. Each point, representing a mean individual (calculated from the 16 fry sampled per rack), was plotted for the first and second axes for the purpose of clarity. The groups are based on a hierarchical cluster analysis.

The PCA for factors influencing hatching enabled us to distinguish two principal axes that explained 65% of the variance (Fig 6(A)). The first axis (47% of the variance) was correlated with four morpho-anatomical parameters: ED (*r* = 0.49), TL (*r* = 0.48), HM (*r* = 0.45), and YSD (*r* = 0.48). The second axis explained 19% of the variance and was correlated with MY (*r* = 0.66) and MT (*r* = - 0.42). At hatching, five clusters were identified by the hierarchical cluster analysis, essentially based on the fry length but not on the mass of yolk sac or tissue (Fig 6(A)). A first cluster (12°C) displayed the shortest individuals with the smallest yolk sac. The second and the third clusters (10°C, 8°C) grouped fry that were of intermediate length and had an intermediate yolk sac. The fourth cluster (6°C) was characterized by longer individuals with a larger yolk sac and the fifth cluster at 4°C was characterized by the longest individuals with the largest yolk sac.

The PCA for factors influencing emergence enabled us to distinguish two principal axes that explained 52% of the variance (Fig 6(B)). The first axis (34% of the variance) was correlated with three parameters: HM (*r* = 0.55), ED (*r* = 0.55), and TL (*r* = 0.48). The second axis explained 18% of the variance and was principally correlated with MY (*r* = 0.65), YSD (*r* = 0.48), and MT (*r* = - 0.51). Five principal clusters were identified. A first cluster (12°C) displayed the shortest (TL) fry with the highest dry tissue mass. The second cluster (10°C) grouped fry that were short with an intermediate yolk sac diameter. The third cluster (6°C) displayed fry with the largest yolk sac diameter, yolk sac dry mass, and average total length. The fourth cluster (8˚C) shared similar traits as the second cluster (10˚C) but with longer fry (TL) with larger myotome than fry at 8°C. The fifth cluster at 4°C displayed the longest fry with the greatest dry tissue mass.

The PCA performed at first food intake enabled us to distinguish two principal axes that explained 82% of the variance (Fig 6(C)). The first axis (70% of the variance) was correlated positively with morpho-anatomical parameters YSD (r = - 0.81) and MY (r = 0.84), and negatively with TL (r = -0.95), HM (r = -0.89), and ED (r = -0.84). The second axis explained 12% of the variance and was mainly correlated with MT (r = - 0.76). Two main clusters were identified. A first cluster (12°C) grouped the longest (TL) fry with the highest dry tissue mass and smallest yolk sac. The second cluster (4°C, 6°C, 8°C, and 10°C) grouped fry with shorter and lighter bodies but heavier yolk sacs. All measures are available in S2 Table.

#### Energy value

**Fig 7:**
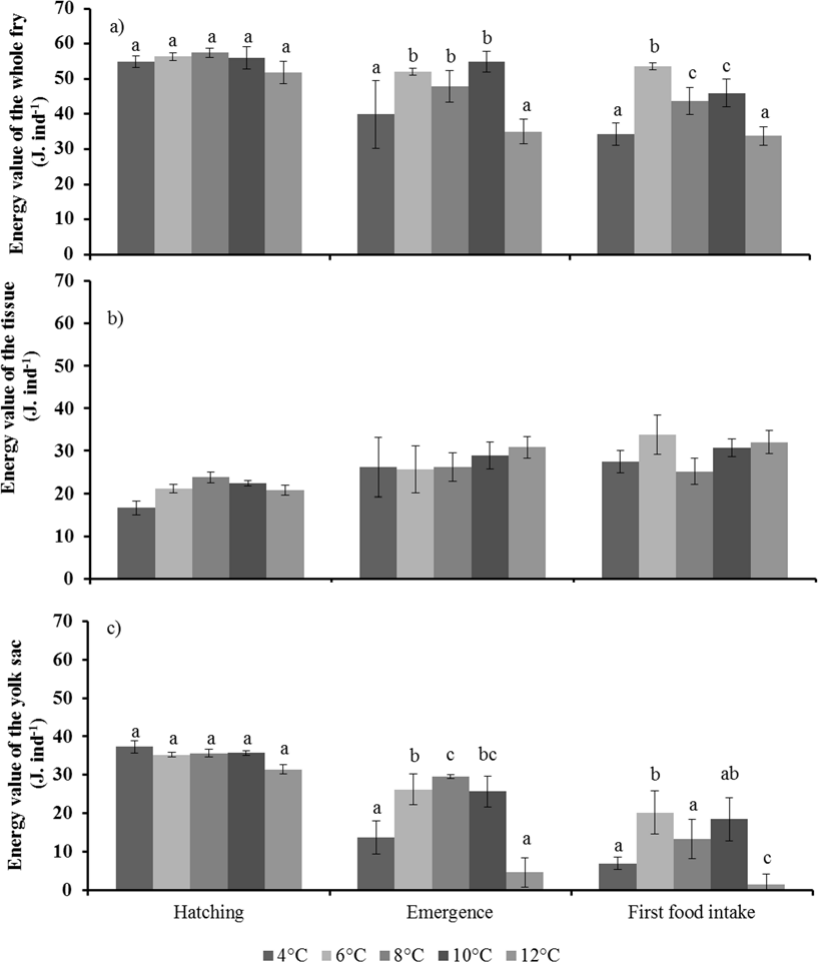
**Effect of temperature on the energy value of fry of brown trout (*Salmo trutta*) at hatching, emergence, and first food intake, a) whole fry, b) tissue, and c) yolk sac.** Mean (± S.D.), with n = 8 (racks). Values with the same letter indicate no significant difference between temperatures for each of the three stages (Tukey, *p* < 0.05).

The energy value (whole individual) varied according to temperatures (GLM, *F*_1,4_ = 55.1, *p* <0.0001) and stages (GLM, *F*_1,2_ = 66.3, *p* < 0.0001), and the interaction between temperatures and stages was significant (GLM: *F*_1,8_ = 14.4, *p* < 0.0001). There was no effect of racks (GLM, *F*_1,35_ = 1.0, *p* = 0.34). At hatching, there was no difference between all temperatures (Fig 7(A)). At emergence, the total energy value was lowest at the two extreme temperatures (4°C and 12°C). At first food intake, the energy value was still lowest at the two extreme temperatures and highest at 6°C (Fig 7(A)). The tissue energy value was significantly different between stages (GLM: *F*_1,2_ = 21.4, *p* < 0.0001) but was not affected by temperature (GLM: *F*_1,8_ = 2.5, *p* = 0.06). There was no effect of racks (GLM: *F*_1,35_ = 1.0, *p* = 0.43) (Fig 7(B)). The yolk sac energy value was significantly different between temperatures (GLM: *F*_1,4_ = 22.1, *p* < 0.0001) and stages (GLM: *F*_1,2_ = 196.6, *p* < 0.0001), and was also influenced by the interaction between temperatures and stages (GLM: *F*_1,8_ = 12.3, *p* < 0.0001). There was no effect of racks (GLM: *F*_1,35_ = 1.3, *p* = 0.19). At hatching, there was no difference between all temperatures (Fig 7(C)). At emergence, the energy value was lowest at the two extreme temperatures. At first food intake, the energy value was significantly lower at 12°C, and highest at 6°C (Fig 7(C)). All data are available in S3 Table and S4 Table.

## Discussion

This experiment explores both the thermal tolerance and phenotypic plasticity of the early life stages of brown trout, supporting previous results obtained chiefly during incubation (from fertilization up to hatching) as well as providing new data from hatching to first food intake. This enables us to have a holistic view of the effects of temperature during the entire endogenous feeding period of brown trout. At first food intake, the survival rate of the fry was only 0.9% at 12°C, and 28% of these surviving fry were malformed. At that stage and temperature, fry were longest with higher dry tissue mass and no remaining yolk sac. At 4°C, the survival rate was highest, yet 22% of the fry were malformed. They were smaller and lighter and had low yolk sac reserve. By contrast, the highest survival of well-formed fry at first food intake was found between 6°C and 8°C.

Daily mortality varied significantly during incubation, with high peaks observed at 6 to 20 days post fertilization. Such peaks in mortality had never been described in brown trout before; however, such an occurrence had been observed at 8 days post fertilization at 10°C in another salmonid species, the rainbow trout *Oncorhynchus mykiss* (21.5% of eggs became white) [32]. This suggests that the earliest ontogenetic stages between fertilization and hatching, in which cell differentiation predominates over growth, may be more sensitive to either mechanical perturbations [33] or unfavorable thermal conditions [33, 37–42, this study]. Moreover, the influence of temperature on the duration of the incubation period of brown trout was studied in widely distant geographic areas: Spain [24], Austria [25], the United States [26], the United Kingdom [43], and Belgium (this study). Similar trends to our results were found, particularly for 6°C, 8°C, and 10°C, confirming the expected negative curvilinear relationship between development time and temperature [24, 44].

At hatching, the highest survival rates for well-formed fry were found at the lowest three temperatures. These results were similar to those obtained previously (75% at 6°C-8°C and 9% at 12°C in the present study compared with 68% at 7°C-9°C and 3% at 13°C [25]). The slightly different results of survival at hatching for the same temperatures might reflect local adaptations of thermal tolerance between trout populations living in different environments or at different latitudes (Spain, Belgium, and Austria). Such a hypothesis could be tested by incubating embryos from different populations under the same conditions and compare their thermal tolerance, a method known as common garden experiment [45]. At first food intake, the survival rate was only 0.9% at 12°C, whereas it was above 70% from 4°C to 8°C; to our knowledge, there are no data available in the literature, especially on the daily mortality between hatching and emergence, on brown trout to compare to our results.

The increase in deformities during incubation at temperatures close to the brown trout’s lower and upper limits is a well-known phenomenon for this species as well as for other fish [25, 46–48]. Embody [26] observed that malformations were more common at hatching at temperatures below 3°C or above 10°C. Lahnsteiner [25] also observed a strong increase in malformations at 13°C, which was also the case here as we found an increase in deformities at 12°C upon hatching. It could be due to the fact that fry are not able to rectify their head and vertebral column after leaving the egg shell [41, 47–51]. Moreover, the increase in deformities at 4°C after emergence could be a consequence of an underinflated swim bladder [52], particularly when fry start swimming [48, 53]. This could lead to over activity of the pectoral fins to compensate for the lack of stabilization (buoyancy) and dynamism, which forces fry to swim in an abnormal and oblique way [54].

At hatching, the size of the fry decreased significantly with higher incubation temperatures (see also [24]). The negative relationship between fry size at hatching and incubation temperature could be partly explained by the fact that higher temperatures promote higher metabolic rates. Several studies have already found lower yolk sac conversion efficiency [55], resulting in a smaller body size at hatching, when temperature increased [24, 56–61]. Indeed, the increased energy consumption for both respiration and maintenance, due to an increase in temperature, will result in a decrease in energy for the formation of new tissue [43, 62, 63], and consequently could result in smaller fry at hatching. Such a negative relationship between size at hatching and temperature has already been described in other cold water species, including Atlantic salmon [64], Atlantic cod (*Gadus morhua*) [56,65], and haddock (*Melanogrammus aeglefinus*) [66]. Besides, it is well known that hatching does not occur at a specific age [67] or properly characterize an ontogenetic stage [68, 69], and it has been proposed that smaller fry at hatching are also premature and do not show the same morphology when temperature increases [24, 56]. Some meristic characteristics, such as vertebral and fin ray counts [59], and potential growth [63], have been reported to be related to incubation temperatures.

In the present study, fry were much larger at hatching when compared to those in a previous study [24], even though embryos were incubated at similar temperatures: 20.0 vs. 15.3 mm (4°C), 19.1 vs. 15.4 mm (6°C), 18.2 vs. 15.0 mm (10°C), 17.2 vs. 14.5 mm (12°C), and 15.6 vs. 14.0 mm (12°C). Such differences could be due to the age and probably the sizes of the broodstock that were used: they were 4 years old in this study, (see Materials and Methods), whereas they were 3 years old (no information on the size and mass) in the study by Ojanguren and Braña [24]. Indeed, Pusher and Humpech [70] found a positive relationship between female size, number and volume of eggs, and fry size at hatching. Smaller females (size: 21.0 cm, mass: 83.7 g) have smaller eggs (3.6 mm^3^) and fry are smaller when they hatch (10.0 mm) in contrast to larger females (size: 34.0 cm, mass: 431.0 g), which have bigger eggs (6.9 mm^3^) resulting in larger fry (18.6 mm). The difference observed in fry size at hatching between different studies might also reflect local adaptations of trout populations. Indeed, a positive relationship between egg size (and thus possibly fry size at hatching [70]) and higher latitudes was found in salmonids [71, 72]. This might partly explain why fry obtained from trout coming from Belgium (this study) were larger at hatching then those coming from Spain [24].

At emergence, fry at 4°C were largest, yet with low energy value. Fry reared at 12°C were smallest and lightest, and they also had low energy value. At first food intake, fry reared at both 4°C and 12°C were still different; at 4°C, they were smaller and lighter than at 12°C. This might be because temperature influences the transition to exogenous feeding by modifying the use of the yolk sac, as well as the maturation of the digestive system necessary for initiating exogenous feeding [55]. During the endogenous feeding period, an increase in temperature would result in an increased consumption of the yolk sac due to increased metabolism [33, 58, 61, 63, 68]. Previous studies have demonstrated that temperature may positively influence first food intake stage by modifying the consumption of the yolk sac, the development and maturation of the digestive system, and the onset of exogenous feeding [68, 73]. But, in the present study, fry at 12°C were close to lethal temperature (0.9% survival rate at first food intake) and from anatomical and morphological observations based on [74], they were “premature” at hatching. So we can hypothesize that a lack or incomplete development of morphological (malformations of the head, jaw, spine) and digestive (intestinal atrophy, intestinal flattening folds) structures necessary to achieve the first food intake stage may explain fry status [75,76].

At 12°C, the population virtually disappeared by first food intake. These experimental results confirmed the prediction of current models that the distribution area of brown trout could decrease by as much as 75.9% in France by 2080 [12, 13] if global warming was to result in an increase in water temperature from 8 to 12 degrees. Besides, as these predictions are based on the thermal tolerance of adults, the effect of global warming may be even stronger in view of the results obtained here. Although the survival rate of well-formed fry was greater than 50% at 4°C and 10°C, the energy value was lower when compared to 6°C and 8°C. At 4°C, the decrease in energy available in the yolk sac was significant. The energy value in the yolk sac gives time for fry to learn predatory behaviors (including repeated attacks) and for maturation of the digestive system necessary for the onset of exogenous feeding [77]. This lack of time due to lower energy could influence the point-of-no-return or PNR (corresponding to when the individual will die due to irreversible starvation) for fry [75, 77]. Indeed, if adequate prey items are not sufficiently available, first-feeding fry could die before the onset of irreversible starvation [77]. Duration of the endogenous feeding period can also strongly influence the PNR [24–27, 36, 75]. Indeed, large variations in the time to reach first food intake (4°C and 10°C) could reduce the “window of opportunity” for fry to find adequate prey, a phenomenon known as "match-mismatch" [75, 76, 78–80]. MacLeod et al. [81], while rearing coral fish larvae (*Amphiprion percula*) at three different temperatures (29.2°C, 30.7°C, and 32.2°C) and with different feeding frequencies, found that these two factors affect larval survival, metabolism, and growth. High energy use at higher temperatures led to less energy available for growth, resulting in longer time to metamorphosis, especially when food supplies were low. They suggested that lower amounts of food could be more challenging in natural environments because larvae have to swim and spend more energy. This phenomenon has also been observed in natural conditions, such as in Lake Washington (United States) where, in 2002, the thermal stratification took place 20 days earlier than usual due to warming waters [79]. This caused a shift between the phytoplankton bloom and the presence of consumers, zooplankton (*Daphnia* spp.), which did not respond in the same way to warmer waters. This shift led to a temporary inability for *Daphnia* spp. to feed on phytoplankton. These effects on the micro-fauna had in turn consequences on freshwater communities. Kai et al. [78] also showed that pikeperch (*Sander lucioperca*) populations from Lake Peipsi and Lake Võrtsjärv, two lakes of the Czech Republic, were indirectly affected by global warming. In these lakes, global warming led to a decrease in the number of larger cladocerans. This drop in prey availability was linked to the negative recruitment of juvenile pikeperch [78]. Finally, increasing temperatures could modify several biotic and abiotic factors in the immediate surroundings of brown trout, including hydrology [82]. Notably, these changes might have a negative effect on the benthic (*Gammarus* sp.) and terrestrial (*Musca domestica*) invertebrate communities, on which young trout feed [83]. This could cause changes in the hunting strategies of trout for terrestrial invertebrates at the water surface to benthic prey [84].

## Conclusions

The present study explored the thermal tolerance and phenotypic plasticity of various traits for the early life stages of brown trout. First, optimum survival of well-formed fry at first food intake was determined to be between 6°C and 8°C, temperatures at which the possible contribution to the next generation should be highest. When temperatures increased to 12°C, the population fell dramatically. These results are in accordance with current models that predict a significant decrease in brown trout in France in the coming decades due to global warming. Besides, even though the survival rate was high at 4°C, fry were also markedly affected, as they showed lower energy values at first food intake and their development time was three times longer than at 8°C. This longer development time, along with a lower energy value at first food intake, could reduce the “window of opportunity” for fry to find adequate prey, thus causing their death as a result of irreversible starvation.

## Supporting Information

S1 Table Table of cumulative number of dead individuals per day for each temperature. (DOCX)

S2 Table Table of morphometric measurements for each temperature (cm). Hatching, (H), emergence (EM), and first food intake (Ffo).
(DOCX)

S3 Table Table of composition parameters and energy value for egg and yolk sac at hatching, emergence, and first food intake for each temperature. (DOCX)

S4 Table Table of composition parameters and energy value for tissue at hatching, emergence, and first food intake for each temperature. (DOCX)
