# Supplementary material for: Strong Effects of Temperature on the Early Life Stages of a Cold Stenothermal Fish Species, Brown Trout (Salmo trutta L.)
Source: PLoS One. 2016 May 12;11(5):e0155487. doi: 10.1371/journal.pone.0155487 (PMC4865038; doi:10.1371/journal.pone.0155487)
Supplement: S1 Table — (DOCX) [file pone.0155487.s001.docx]

| Dated | 4°C | 6°C | 8°C | 10°C | 12°C |
| --- | --- | --- | --- | --- | --- |
| 19/12/2013 | unfertilized | | | | |
| 20/12/2013 |  |  |  |  |  |
| 21/12/2013 | 315 | 288 | 225 | 270 | 280 |
| 22/12/2013 | 410 | 360 | 253 | 321 | 389 |
| 23/12/2013 | 249 | 230 | 283 | 246 | 326 |
| 24/12/2013 | 252 | 266 | 334 | 300 | 311 |
| 26/12/2013 | 266 | 308 | 334 | 321 | 459 |
| 27/12/2013 | 294 | 329 | 413 | 330 | 1020 |
| 28/12/2013 | 304 | 341 | 472 | 364 | 1323 |
| 29/12/2013 | 320 | 368 | 572 | 459 | 1350 |
| 30/12/2013 | 332 | 372 | 654 | 560 | 1518 |
| 31/12/2013 | 337 | 386 | 738 | 781 | 1654 |
| 01/01/2014 | 342 | 414 | 797 | 858 | 1781 |
| 02/01/2014 | 345 | 445 | 839 | 915 | 1867 |
| 03/01/2014 | 349 | 494 | 877 | 957 | 1937 |
| 04/01/2014 | 364 | 552 | 913 | 974 | 2013 |
| 05/01/2014 | 377 | 600 | 942 | 993 | 2095 |
| 06/01/2014 | 390 | 631 | 960 | 1007 | 2208 |
| 07/01/2014 | 409 | 665 | 974 | 1033 | 2423 |
| 08/01/2014 | 439 | 685 | 987 | 1050 | 2558 |
| 09/01/2014 | 471 | 707 | 992 | 1060 | 2673 |
| 10/01/2014 | 502 | 710 | 1000 | 1076 | 2783 |
| 11/01/2014 | 533 | 725 | 1013 | 1096 | 2871 |
| 12/01/2014 | 565 | 734 | 1032 | 1116 | 2989 |
| 13/01/2014 | 577 | 736 | 1039 | 1125 | 3151 |
| 14/01/2014 | 605 | 746 | 1044 | 1156 | 3413 |
| 15/01/2014 | 616 | 746 | 1052 | 1173 | 3664 |
| 16/01/2014 | 625 | 752 | 1057 | 1183 | 3901 |
| 17/01/2014 | 635 | 753 | 1064 | 1200 | 4141 |
| 18/01/2014 | 645 | 764 | 1083 | 1223 | 4446 |
| 19/01/2014 | 649 | 768 | 1092 | 1241 | 4660 |
| 20/01/2014 | 652 | 772 | 1101 | 1287 | 4668 |
| 21/01/2014 | 658 | 773 | 1104 | 1320 | 4685 |
| 22/01/2014 | 661 | 776 | 1109 | 1358 | 5005 |
| 23/01/2014 | 663 | 782 | 1118 | 1408 | 5048 |
| 24/01/2014 | 665 | 786 | 1124 | 1453 | 5098 |
| 25/01/2014 | 669 | 791 | 1132 | 1503 | 5265 |
| 26/01/2014 | 676 | 794 | 1144 | 1549 | 5265 |
| 27/01/2014 | 679 | 796 | 1156 | 1654 | 5267 |
| 28/01/2014 | 681 | 798 | 1179 | 1796 | 5269 |
| 29/01/2014 | 686 | 803 | 1203 | 1888 | 5300 |
| 30/01/2014 | 687 | 809 | 1214 | 1953 | 5400 |
| 31/01/2014 | 688 | 809 | 1238 | 2069 | 5476 |
| 01/02/2014 | 688 | 812 | 1317 | 2105 | 5476 |
| 02/02/2014 | 690 | 815 | 1372 | 2250 | 5480 |
| 03/02/2014 | 694 | 817 | 1425 | 2288 | 5482 |
| 04/02/2014 | 694 | 821 | 1427 | 2302 | 5482 |
| 05/02/2014 | 696 | 825 | 1429 | 2314 | 5485 |
| 06/02/2014 | 699 | 829 | 1439 | 2438 | 5485 |
| 07/02/2014 | 699 | 831 | 1446 | 2544 | 5485 |
| 08/02/2014 | 706 | 835 | 1485 | 2547 | 5485 |
| 09/02/2014 | 707 | 840 | 1541 | 2560 | 5486 |
| 10/02/2014 | 712 | 845 | 1542 | 2600 | 5488 |
| 11/02/2014 | 719 | 854 | 1544 | 2602 | 5488 |
| 12/02/2014 | 723 | 860 | 1544 | 2605 |  |
| 13/02/2014 | 725 | 867 | 1544 |  |  |
| 14/02/2014 | 728 | 870 | 1544 |  |  |
| 15/02/2014 | 728 | 876 | 1546 |  |  |
| 16/02/2014 | 734 | 980 | 1547 |  |  |
| 17/02/2014 | 744 | 1043 | 1547 |  |  |
| 18/02/2014 | 747 | 1051 | 1547 |  |  |
| 19/02/2014 | 751 | 1059 | 1547 |  |  |
| 20/02/2014 | 753 | 1095 | 1550 |  |  |
| 21/02/2014 | 756 | 1095 | 1551 |  |  |
| 22/02/2014 | 771 | 1096 | 1551 |  |  |
| 23/02/2014 | 805 | 1096 | 1551 |  |  |
| 24/02/2014 | 830 | 1096 | 1551 |  |  |
| 25/02/2014 | 864 | 1096 |  |  |  |
| 26/02/2014 | 868 | 1096 |  |  |  |
| 27/02/2014 | 881 | 1098 |  |  |  |
| 28/02/2014 | 900 | 1099 |  |  |  |
| 01/03/2014 | 929 | 1100 |  |  |  |
| 02/03/2014 | 944 | 1101 |  |  |  |
| 03/03/2014 | 962 | 1105 |  |  |  |
| 04/03/2014 | 1001 | 1105 |  |  |  |
| 05/03/2014 | 1026 | 1105 |  |  |  |
| 06/03/2014 | 1036 | 1110 |  |  |  |
| 07/03/2014 | 1050 | 1110 |  |  |  |
| 08/03/2014 | 1056 | 1112 |  |  |  |
| 09/03/2014 | 1070 | 1113 |  |  |  |
| 10/03/2014 | 1079 | 1113 |  |  |  |
| 11/03/2014 | 1082 | 1115 |  |  |  |
| 12/03/2014 | 1094 | 1117 |  |  |  |
| 13/03/2014 | 1105 | 1118 |  |  |  |
| 14/03/2014 | 1113 | 1119 |  |  |  |
| 15/03/2014 | 1116 | 1119 |  |  |  |
| 16/03/2014 | 1117 |  |  |  |  |
| 17/03/2014 | 1120 |  |  |  |  |
| 18/03/2014 | 1123 |  |  |  |  |
| 19/03/2014 | 1126 |  |  |  |  |
| 20/03/2014 | 1134 |  |  |  |  |
| 21/03/2014 | 1144 |  |  |  |  |
| 22/03/2014 | 1153 |  |  |  |  |
| 23/03/2014 | 1048 |  |  |  |  |
| 24/03/2014 | 1166 |  |  |  |  |
| 25/03/2014 | 1174 |  |  |  |  |
| 26/03/2014 | 1176 |  |  |  |  |
| 27/03/2014 | 1192 |  |  |  |  |
| 28/03/2014 | 1197 |  |  |  |  |
| 29/03/2014 | 1200 |  |  |  |  |
| 30/03/2014 | 1207 |  |  |  |  |
| 31/03/2014 | 1217 |  |  |  |  |
| 01/04/2014 | 1226 |  |  |  |  |
| 02/04/2014 | 1228 |  |  |  |  |
| 03/04/2014 | 1232 |  |  |  |  |
| 04/04/2014 | 1232 |  |  |  |  |
| 05/04/2014 | 1236 |  |  |  |  |
| 06/04/2014 | 1236 |  |  |  |  |
| 07/04/2014 | 1238 |  |  |  |  |
| 08/04/2014 | 1241 |  |  |  |  |
| 09/04/2014 | 1241 |  |  |  |  |
| 10/04/2014 | 1245 |  |  |  |  |
| 11/04/2014 | 1307 |  |  |  |  |
| 12/04/2014 | 1354 |  |  |  |  |
| 13/04/2014 | 1357 |  |  |  |  |
| 14/04/2014 | 1383 |  |  |  |  |
| 15/04/2014 | 1400 |  |  |  |  |
| 16/04/2014 | 1403 |  |  |  |  |
| 17/04/2014 | 1406 |  |  |  |  |
| 18/04/2014 | 1415 |  |  |  |  |
| 19/04/2014 | 1420 |  |  |  |  |
| 20/04/2014 | 1425 |  |  |  |  |
| 21/04/2014 | 1425 |  |  |  |  |
| 22/04/2014 | 1425 |  |  |  |  |
| 23/04/2014 | 1425 |  |  |  |  |
| 24/04/2014 | 1425 |  |  |  |  |
| 25/04/2014 | 1425 |  |  |  |  |
| 26/04/2014 | 1425 |  |  |  |  |
| Nb of total individus | 5938 | 5080 | 5349 | 4737 | 6098 |
