# Supplementary material for: Strong Effects of Temperature on the Early Life Stages of a Cold Stenothermal Fish Species, Brown Trout (Salmo trutta L.)
Source: PLoS One. 2016 May 12;11(5):e0155487. doi: 10.1371/journal.pone.0155487 (PMC4865038; doi:10.1371/journal.pone.0155487)
Supplement: S2 Table — Hatching, (H), emergence (EM), and first food intake (Ffo). (DOCX) [file pone.0155487.s002.docx]

| Temperature | Biological stages | Total length | Average diameter of the yolk sac | Avarage Diameter of the eye | Height myotom |
| --- | --- | --- | --- | --- | --- |
| 4°C | H | 19.82 | 6.86 | 0.11 | 0.17 |
| 4°C | H | 19.92 | 7.15 | 0.13 | 0.20 |
| 4°C | H | 18.62 | 7.24 | 0.11 | 0.18 |
| 4°C | H | 20.01 | 7.44 | 0.14 | 0.18 |
| 4°C | H | 19.74 | 7.91 | 0.14 | 0.18 |
| 4°C | H | 20.50 | 8.88 | 0.13 | 0.20 |
| 4°C | H | 20.47 | 7.69 | 0.15 | 0.19 |
| 4°C | H | 20.85 | 8.42 | 0.11 | 0.19 |
| 4°C | H | 20.91 | 8.69 | 0.14 | 0.18 |
| 4°C | H | 19.93 | 7.67 | 0.15 | 0.19 |
| 4°C | H | 19.99 | 8.84 | 0.16 | 0.17 |
| 4°C | H | 19.37 | 7.73 | 0.13 | 0.19 |
| 4°C | H | 21.27 | 9.06 | 0.12 | 0.18 |
| 4°C | H | 20.71 | 8.86 | 0.12 | 0.19 |
| 4°C | H | 20.82 | 7.77 | 0.12 | 0.16 |
| 4°C | H | 18.06 | 7.63 | 0.13 | 0.17 |
| 4°C | H | 19.54 | 7.35 | 0.14 | 0.17 |
| 4°C | H | 19.00 | 8.43 | 0.11 | 0.18 |
| 4°C | H | 19.35 | 7.85 | 0.14 | 0.18 |
| 4°C | H | 20.73 | 7.86 | 0.14 | 0.17 |
| 4°C | H | 19.32 | 8.16 | 0.12 | 0.18 |
| 4°C | H | 19.40 | 7.80 | 0.12 | 0.16 |
| 4°C | H | 18.31 | 5.50 | 0.11 | 0.19 |
| 4°C | H | 20.09 | 7.93 | 0.14 | 0.18 |
| 4°C | H | 20.45 | 7.80 | 0.12 | 0.16 |
| 4°C | H | 19.17 | 7.60 | 0.13 | 0.18 |
| 4°C | H | 21.00 | 7.68 | 0.14 | 0.18 |
| 4°C | H | 20.68 | 7.49 | 0.14 | 0.17 |
| 4°C | H | 20.90 | 9.14 | 0.12 | 0.19 |
| 4°C | H | 20.32 | 8.29 | 0.15 | 0.17 |
| 4°C | H | 19.84 | 7.88 | 0.13 | 0.18 |
| 4°C | H | 20.16 | 7.79 | 0.12 | 0.19 |
| 4°C | H | 20.76 | 8.54 | 0.15 | 0.17 |
| 4°C | H | 19.77 | 8.16 | 0.13 | 0.18 |
| 4°C | H | 19.65 | 8.42 | 0.15 | 0.18 |
| 4°C | H | 18.97 | 8.50 | 0.13 | 0.17 |
| 4°C | H | 19.68 | 6.80 | 0.12 | 0.18 |
| 4°C | H | 19.41 | 8.50 | 0.13 | 0.19 |
| 4°C | H | 20.55 | 8.53 | 0.16 | 0.17 |
| 4°C | H | 18.19 | 7.75 | 0.15 | 0.18 |
| 4°C | H | 19.92 | 9.16 | 0.11 | 0.18 |
| 4°C | H | 20.81 | 8.86 | 0.14 | 0.18 |
| 4°C | H | 20.91 | 8.68 | 0.15 | 0.19 |
| 4°C | H | 21.12 | 9.12 | 0.14 | 0.18 |
| 4°C | H | 20.97 | 9.19 | 0.12 | 0.15 |
| 4°C | H | 19.27 | 6.65 | 0.13 | 0.15 |
| 4°C | H | 19.27 | 6.65 | 0.13 | 0.18 |
| 4°C | H | 19.36 | 8.15 | 0.14 | 0.19 |
| 4°C | H | 20.55 | 7.63 | 0.12 | 0.19 |
| 4°C | H | 20.91 | 7.74 | 0.16 | 0.18 |
| 4°C | H | 19.87 | 7.06 | 0.13 | 0.17 |
| 4°C | H | 19.84 | 7.37 | 0.14 | 0.19 |
| 4°C | H | 19.95 | 7.37 | 0.15 | 0.18 |
| 4°C | H | 19.34 | 7.51 | 0.13 | 0.19 |
| 4°C | H | 20.51 | 8.47 | 0.13 | 0.17 |
| 4°C | H | 20.60 | 7.58 | 0.15 | 0.19 |
| 4°C | H | 20.51 | 7.88 | 0.13 | 0.20 |
| 4°C | H | 20.85 | 9.42 | 0.14 | 0.19 |
| 4°C | H | 20.02 | 8.61 | 0.15 | 0.20 |
| 4°C | H | 20.82 | 8.07 | 0.13 | 0.17 |
| 4°C | H | 19.88 | 7.24 | 0.11 | 0.17 |
| 4°C | H | 19.80 | 7.27 | 0.12 | 0.18 |
| 4°C | H | 20.60 | 8.71 | 0.15 | 0.20 |
| 4°C | H | 20.73 | 8.21 | 0.12 | 0.18 |
| 4°C | H | 20.35 | 7.15 | 0.13 | 0.17 |
| 4°C | H | 21.34 | 8.42 | 0.14 | 0.18 |
| 4°C | H | 20.22 | 7.15 | 0.13 | 0.18 |
| 4°C | H | 20.51 | 7.87 | 0.14 | 0.18 |
| 4°C | H | 20.51 | 7.87 | 0.14 | 0.17 |
| 4°C | H | 20.34 | 6.95 | 0.12 | 0.18 |
| 4°C | H | 20.38 | 7.95 | 0.13 | 0.18 |
| 4°C | H | 19.64 | 7.25 | 0.14 | 0.18 |
| 4°C | H | 20.22 | 8.23 | 0.12 | 0.17 |
| 4°C | H | 20.19 | 8.37 | 0.12 | 0.19 |
| 4°C | H | 19.69 | 7.40 | 0.11 | 0.18 |
| 4°C | H | 19.78 | 7.18 | 0.13 | 0.17 |
| 4°C | H | 19.34 | 7.29 | 0.11 | 0.18 |
| 4°C | H | 20.25 | 7.31 | 0.12 | 0.17 |
| 4°C | H | 19.48 | 7.25 | 0.13 | 0.19 |
| 4°C | H | 19.43 | 7.24 | 0.14 | 0.19 |
| 4°C | H | 20.69 | 6.91 | 0.13 | 0.15 |
| 4°C | H | 18.95 | 6.78 | 0.13 | 0.17 |
| 4°C | H | 19.97 | 7.73 | 0.15 | 0.19 |
| 4°C | H | 20.81 | 7.09 | 0.12 | 0.17 |
| 4°C | H | 18.53 | 7.72 | 0.13 | 0.18 |
| 4°C | H | 20.58 | 7.24 | 0.12 | 0.19 |
| 4°C | H | 20.78 | 7.31 | 0.14 | 0.20 |
| 4°C | H | 20.91 | 7.41 | 0.14 | 0.20 |
| 4°C | H | 20.34 | 7.92 | 0.15 | 0.19 |
| 4°C | H | 19.23 | 7.32 | 0.12 | 0.18 |
| 4°C | H | 19.28 | 7.30 | 0.14 | 0.17 |
| 4°C | H | 18.42 | 7.24 | 0.15 | 0.17 |
| 4°C | H | 19.39 | 7.76 | 0.12 | 0.19 |
| 4°C | H | 20.63 | 6.99 | 0.12 | 0.19 |
| 4°C | H | 19.99 | 8.93 | 0.14 | 0.18 |
| 4°C | H | 19.15 | 7.21 | 0.12 | 0.18 |
| 4°C | H | 19.78 | 7.96 | 0.14 | 0.17 |
| 4°C | H | 19.25 | 8.05 | 0.14 | 0.18 |
| 4°C | H | 18.77 | 7.26 | 0.12 | 0.16 |
| 4°C | H | 19.57 | 7.73 | 0.12 | 0.17 |
| 4°C | H | 19.50 | 9.01 | 0.12 | 0.17 |
| 4°C | H | 19.13 | 8.18 | 0.12 | 0.17 |
| 4°C | H | 19.44 | 8.15 | 0.12 | 0.17 |
| 4°C | H | 19.90 | 7.46 | 0.15 | 0.17 |
| 4°C | H | 19.21 | 7.12 | 0.12 | 0.18 |
| 4°C | H | 19.65 | 7.46 | 0.16 | 0.19 |
| 4°C | H | 19.77 | 7.79 | 0.14 | 0.19 |
| 4°C | H | 19.62 | 7.77 | 0.12 | 0.17 |
| 4°C | H | 19.47 | 7.48 | 0.11 | 0.17 |
| 4°C | H | 19.35 | 7.15 | 0.15 | 0.17 |
| 4°C | H | 20.00 | 6.84 | 0.11 | 0.17 |
| 4°C | H | 20.10 | 7.02 | 0.14 | 0.16 |
| 4°C | H | 21.21 | 8.11 | 0.12 | 0.17 |
| 4°C | H | 18.58 | 8.07 | 0.12 | 0.18 |
| 4°C | H | 19.16 | 7.06 | 0.14 | 0.17 |
| 4°C | H | 20.15 | 7.36 | 0.15 | 0.17 |
| 4°C | H | 18.70 | 7.37 | 0.14 | 0.19 |
| 4°C | H | 20.27 | 7.89 | 0.14 | 0.18 |
| 4°C | EM | 24.86 | 7.54 | 0.18 | 0.26 |
| 4°C | EM | 26.58 | 7.12 | 0.16 | 0.24 |
| 4°C | EM | 23.62 | 7.26 | 0.17 | 0.26 |
| 4°C | EM | 24.26 | 8.00 | 0.18 | 0.23 |
| 4°C | EM | 25.93 | 7.82 | 0.15 | 0.26 |
| 4°C | EM | 26.07 | 8.34 | 0.16 | 0.25 |
| 4°C | EM | 24.37 | 7.99 | 0.16 | 0.23 |
| 4°C | EM | 24.18 | 7.73 | 0.15 | 0.22 |
| 4°C | EM | 23.73 | 8.43 | 0.15 | 0.22 |
| 4°C | EM | 24.93 | 7.70 | 0.17 | 0.24 |
| 4°C | EM | 24.00 | 8.32 | 0.16 | 0.22 |
| 4°C | EM | 24.38 | 7.77 | 0.18 | 0.23 |
| 4°C | EM | 24.76 | 8.42 | 0.15 | 0.25 |
| 4°C | EM | 23.94 | 7.72 | 0.14 | 0.22 |
| 4°C | EM | 23.74 | 7.23 | 0.14 | 0.24 |
| 4°C | EM | 23.72 | 8.40 | 0.15 | 0.22 |
| 4°C | EM | 24.48 | 8.51 | 0.15 | 0.20 |
| 4°C | EM | 25.17 | 7.71 | 0.14 | 0.23 |
| 4°C | EM | 25.69 | 7.55 | 0.15 | 0.24 |
| 4°C | EM | 24.51 | 8.33 | 0.17 | 0.25 |
| 4°C | EM | 24.28 | 7.96 | 0.18 | 0.23 |
| 4°C | EM | 25.33 | 8.22 | 0.15 | 0.24 |
| 4°C | EM | 24.49 | 8.88 | 0.15 | 0.23 |
| 4°C | EM | 24.94 | 7.94 | 0.18 | 0.24 |
| 4°C | EM | 24.46 | 7.31 | 0.17 | 0.23 |
| 4°C | EM | 25.05 | 9.02 | 0.17 | 0.26 |
| 4°C | EM | 25.10 | 8.11 | 0.16 | 0.25 |
| 4°C | EM | 25.79 | 7.79 | 0.15 | 0.24 |
| 4°C | EM | 24.83 | 7.85 | 0.18 | 0.22 |
| 4°C | EM | 25.34 | 8.24 | 0.17 | 0.24 |
| 4°C | EM | 24.91 | 9.28 | 0.14 | 0.22 |
| 4°C | EM | 24.61 | 7.86 | 0.18 | 0.23 |
| 4°C | EM | 24.07 | 9.99 | 0.17 | 0.22 |
| 4°C | EM | 24.92 | 7.32 | 0.17 | 0.23 |
| 4°C | EM | 24.45 | 8.99 | 0.17 | 0.22 |
| 4°C | EM | 26.05 | 7.72 | 0.17 | 0.23 |
| 4°C | EM | 26.03 | 7.98 | 0.14 | 0.24 |
| 4°C | EM | 25.77 | 7.14 | 0.14 | 0.25 |
| 4°C | EM | 25.03 | 8.60 | 0.16 | 0.22 |
| 4°C | EM | 26.32 | 7.16 | 0.16 | 0.25 |
| 4°C | EM | 24.49 | 8.84 | 0.17 | 0.24 |
| 4°C | EM | 25.63 | 8.53 | 0.18 | 0.25 |
| 4°C | EM | 25.93 | 8.77 | 0.16 | 0.25 |
| 4°C | EM | 24.46 | 8.75 | 0.15 | 0.22 |
| 4°C | EM | 24.71 | 9.24 | 0.15 | 0.23 |
| 4°C | EM | 23.72 | 7.00 | 0.14 | 0.23 |
| 4°C | EM | 25.44 | 7.55 | 0.15 | 0.24 |
| 4°C | EM | 25.46 | 8.99 | 0.16 | 0.23 |
| 4°C | EM | 25.24 | 8.82 | 0.15 | 0.23 |
| 4°C | EM | 24.49 | 7.77 | 0.15 | 0.23 |
| 4°C | EM | 23.88 | 8.28 | 0.17 | 0.24 |
| 4°C | EM | 25.27 | 7.93 | 0.18 | 0.24 |
| 4°C | EM | 24.34 | 7.55 | 0.17 | 0.25 |
| 4°C | EM | 25.59 | 7.33 | 0.18 | 0.24 |
| 4°C | EM | 23.72 | 8.80 | 0.15 | 0.24 |
| 4°C | EM | 24.83 | 8.36 | 0.17 | 0.24 |
| 4°C | EM | 24.36 | 8.45 | 0.15 | 0.22 |
| 4°C | EM | 24.86 | 8.40 | 0.15 | 0.25 |
| 4°C | EM | 24.28 | 8.40 | 0.15 | 0.23 |
| 4°C | EM | 25.05 | 8.42 | 0.15 | 0.24 |
| 4°C | EM | 26.01 | 7.79 | 0.18 | 0.23 |
| 4°C | EM | 25.62 | 8.18 | 0.17 | 0.25 |
| 4°C | EM | 25.48 | 7.75 | 0.16 | 0.24 |
| 4°C | EM | 25.41 | 7.80 | 0.15 | 0.24 |
| 4°C | EM | 25.55 | 7.97 | 0.16 | 0.26 |
| 4°C | EM | 25.42 | 7.77 | 0.14 | 0.25 |
| 4°C | EM | 24.67 | 7.95 | 0.18 | 0.24 |
| 4°C | EM | 25.01 | 8.26 | 0.18 | 0.23 |
| 4°C | EM | 24.81 | 7.62 | 0.17 | 0.22 |
| 4°C | EM | 25.52 | 7.68 | 0.14 | 0.25 |
| 4°C | EM | 25.25 | 9.33 | 0.15 | 0.24 |
| 4°C | EM | 16.52 | 7.11 | 0.15 | 0.23 |
| 4°C | EM | 25.65 | 9.14 | 0.16 | 0.23 |
| 4°C | EM | 25.60 | 8.20 | 0.18 | 0.24 |
| 4°C | EM | 24.24 | 8.14 | 0.15 | 0.23 |
| 4°C | EM | 24.94 | 8.04 | 0.16 | 0.25 |
| 4°C | EM | 23.98 | 8.12 | 0.15 | 0.21 |
| 4°C | EM | 25.25 | 7.92 | 0.18 | 0.24 |
| 4°C | EM | 25.87 | 8.65 | 0.18 | 0.25 |
| 4°C | EM | 25.73 | 7.89 | 0.15 | 0.25 |
| 4°C | EM | 26.41 | 7.75 | 0.16 | 0.25 |
| 4°C | EM | 22.38 | 5.32 | 0.15 | 0.18 |
| 4°C | EM | 24.96 | 6.82 | 0.18 | 0.24 |
| 4°C | EM | 24.68 | 7.81 | 0.15 | 0.23 |
| 4°C | EM | 25.01 | 8.22 | 0.17 | 0.24 |
| 4°C | EM | 24.81 | 7.87 | 0.14 | 0.24 |
| 4°C | EM | 23.74 | 9.05 | 0.18 | 0.20 |
| 4°C | EM | 24.97 | 6.73 | 0.15 | 0.23 |
| 4°C | EM | 25.39 | 6.85 | 0.18 | 0.23 |
| 4°C | EM | 26.52 | 8.39 | 0.16 | 0.24 |
| 4°C | EM | 21.67 | 8.28 | 0.14 | 0.18 |
| 4°C | EM | 24.29 | 8.04 | 0.18 | 0.22 |
| 4°C | EM | 25.05 | 7.57 | 0.15 | 0.23 |
| 4°C | EM | 25.49 | 7.10 | 0.15 | 0.25 |
| 4°C | EM | 25.90 | 7.51 | 0.17 | 0.25 |
| 4°C | EM | 24.13 | 8.81 | 0.15 | 0.23 |
| 4°C | EM | 24.91 | 7.98 | 0.17 | 0.23 |
| 4°C | EM | 24.60 | 8.48 | 0.18 | 0.23 |
| 4°C | EM | 24.89 | 7.95 | 0.15 | 0.24 |
| 4°C | EM | 24.53 | 8.19 | 0.15 | 0.22 |
| 4°C | Ffo | 26.30 | 0.72 | 7.25 | 0.27 |
| 4°C | Ffo | 26.00 | 0.72 | 7.18 | 0.26 |
| 4°C | Ffo | 25.57 | 0.71 | 7.12 | 0.25 |
| 4°C | Ffo | 25.74 | 7.66 | 0.15 | 0.27 |
| 4°C | Ffo | 25.94 | 6.86 | 0.15 | 0.27 |
| 4°C | Ffo | 26.85 | 7.61 | 0.15 | 0.26 |
| 4°C | Ffo | 25.78 | 7.15 | 0.16 | 0.28 |
| 4°C | Ffo | 27.23 | 7.70 | 0.18 | 0.27 |
| 4°C | Ffo | 25.80 | 6.90 | 0.16 | 0.26 |
| 4°C | Ffo | 26.49 | 7.43 | 0.17 | 0.28 |
| 4°C | Ffo | 27.32 | 7.08 | 0.17 | 0.28 |
| 4°C | Ffo | 25.33 | 8.05 | 0.17 | 0.27 |
| 4°C | Ffo | 25.87 | 8.22 | 0.16 | 0.27 |
| 4°C | Ffo | 26.48 | 7.15 | 0.15 | 0.27 |
| 4°C | Ffo | 25.97 | 8.12 | 0.16 | 0.27 |
| 4°C | Ffo | 25.86 | 8.01 | 0.17 | 0.26 |
| 4°C | Ffo | 25.93 | 8.21 | 0.18 | 0.27 |
| 4°C | Ffo | 25.67 | 7.27 | 0.18 | 0.28 |
| 4°C | Ffo | 26.76 | 7.06 | 0.19 | 0.26 |
| 4°C | Ffo | 26.06 | 7.35 | 0.16 | 0.26 |
| 4°C | Ffo | 26.27 | 7.80 | 0.18 | 0.28 |
| 4°C | Ffo | 27.21 | 8.11 | 0.18 | 0.30 |
| 4°C | Ffo | 26.88 | 8.15 | 0.18 | 0.27 |
| 4°C | Ffo | 26.06 | 8.18 | 0.19 | 0.26 |
| 4°C | Ffo | 25.69 | 6.85 | 0.16 | 0.28 |
| 4°C | Ffo | 25.75 | 6.94 | 0.19 | 0.28 |
| 4°C | Ffo | 25.14 | 8.31 | 0.17 | 0.27 |
| 4°C | Ffo | 26.48 | 7.06 | 0.15 | 0.27 |
| 4°C | Ffo | 27.24 | 8.39 | 0.15 | 0.29 |
| 4°C | Ffo | 25.63 | 8.11 | 0.19 | 0.26 |
| 4°C | Ffo | 27.44 | 8.17 | 0.17 | 0.27 |
| 4°C | Ffo | 27.30 | 7.27 | 0.16 | 0.28 |
| 4°C | Ffo | 24.97 | 7.24 | 0.15 | 0.27 |
| 4°C | Ffo | 25.23 | 6.82 | 0.16 | 0.27 |
| 4°C | Ffo | 25.50 | 7.02 | 0.18 | 0.28 |
| 4°C | Ffo | 25.47 | 7.57 | 0.16 | 0.26 |
| 4°C | Ffo | 27.01 | 7.35 | 0.16 | 0.27 |
| 4°C | Ffo | 25.97 | 7.74 | 0.15 | 0.28 |
| 4°C | Ffo | 27.15 | 7.94 | 0.16 | 0.27 |
| 4°C | Ffo | 25.19 | 7.54 | 0.19 | 0.27 |
| 4°C | Ffo | 26.67 | 6.83 | 0.16 | 0.28 |
| 4°C | Ffo | 26.77 | 8.03 | 0.17 | 0.29 |
| 4°C | Ffo | 25.14 | 6.60 | 0.16 | 0.26 |
| 4°C | Ffo | 25.42 | 7.25 | 0.15 | 0.26 |
| 4°C | Ffo | 25.54 | 7.95 | 0.17 | 0.26 |
| 4°C | Ffo | 26.46 | 8.07 | 0.16 | 0.27 |
| 4°C | Ffo | 25.64 | 7.16 | 0.18 | 0.27 |
| 4°C | Ffo | 26.52 | 7.53 | 0.17 | 0.28 |
| 4°C | Ffo | 26.55 | 7.34 | 0.17 | 0.26 |
| 4°C | Ffo | 26.94 | 7.52 | 0.17 | 0.28 |
| 4°C | Ffo | 25.86 | 7.37 | 0.17 | 0.27 |
| 4°C | Ffo | 26.18 | 7.73 | 0.18 | 0.26 |
| 4°C | Ffo | 25.28 | 7.67 | 0.20 | 0.26 |
| 4°C | Ffo | 25.89 | 7.34 | 0.16 | 0.28 |
| 4°C | Ffo | 25.93 | 7.98 | 0.16 | 0.29 |
| 4°C | Ffo | 26.79 | 7.52 | 0.16 | 0.28 |
| 4°C | Ffo | 27.34 | 8.00 | 0.15 | 0.28 |
| 4°C | Ffo | 25.47 | 8.14 | 0.15 | 0.26 |
| 4°C | Ffo | 27.28 | 6.85 | 0.16 | 0.24 |
| 4°C | Ffo | 25.09 | 8.03 | 0.15 | 0.24 |
| 4°C | Ffo | 27.47 | 8.13 | 0.15 | 0.28 |
| 4°C | Ffo | 26.96 | 7.47 | 0.16 | 0.27 |
| 4°C | Ffo | 26.41 | 7.68 | 0.16 | 0.27 |
| 4°C | Ffo | 25.52 | 7.44 | 0.17 | 0.26 |
| 4°C | Ffo | 24.72 | 7.51 | 0.18 | 0.25 |
| 4°C | Ffo | 26.50 | 7.01 | 0.16 | 0.28 |
| 4°C | Ffo | 22.33 | 5.37 | 0.15 | 0.21 |
| 4°C | Ffo | 24.64 | 7.94 | 0.15 | 0.26 |
| 4°C | Ffo | 25.68 | 7.84 | 0.16 | 0.25 |
| 4°C | Ffo | 25.64 | 7.40 | 0.15 | 0.25 |
| 4°C | Ffo | 25.93 | 8.89 | 0.15 | 0.27 |
| 4°C | Ffo | 25.44 | 7.57 | 0.14 | 0.27 |
| 4°C | Ffo | 25.72 | 7.08 | 0.14 | 0.27 |
| 4°C | Ffo | 25.56 | 7.78 | 0.18 | 0.25 |
| 4°C | Ffo | 23.55 | 7.89 | 0.16 | 0.24 |
| 4°C | Ffo | 24.94 | 7.21 | 0.14 | 0.25 |
| 4°C | Ffo | 25.59 | 7.61 | 0.16 | 0.25 |
| 4°C | Ffo | 25.07 | 7.93 | 0.15 | 0.25 |
| 4°C | Ffo | 25.83 | 7.81 | 0.16 | 0.24 |
| 4°C | Ffo | 27.22 | 8.03 | 0.16 | 0.26 |
| 4°C | Ffo | 25.89 | 8.22 | 0.15 | 0.26 |
| 4°C | Ffo | 25.27 | 8.69 | 0.16 | 0.26 |
| 4°C | Ffo | 27.07 | 7.61 | 0.16 | 0.27 |
| 4°C | Ffo | 25.38 | 7.70 | 0.15 | 0.26 |
| 4°C | Ffo | 26.78 | 7.48 | 0.15 | 0.26 |
| 4°C | Ffo | 25.11 | 7.58 | 0.15 | 0.23 |
| 4°C | Ffo | 25.86 | 8.47 | 0.15 | 0.26 |
| 4°C | Ffo | 25.49 | 8.13 | 0.17 | 0.27 |
| 4°C | Ffo | 25.22 | 6.85 | 0.16 | 0.26 |
| 4°C | Ffo | 24.68 | 6.81 | 0.14 | 0.28 |
| 4°C | Ffo | 26.15 | 6.80 | 0.16 | 0.26 |
| 4°C | Ffo | 23.95 | 6.66 | 0.15 | 0.24 |
| 4°C | Ffo | 23.66 | 7.94 | 0.15 | 0.25 |
| 4°C | Ffo | 24.92 | 8.08 | 0.16 | 0.25 |
| 4°C | Ffo | 25.19 | 7.21 | 0.15 | 0.24 |
| 4°C | Ffo | 25.70 | 6.58 | 0.15 | 0.28 |
| 4°C | Ffo | 25.79 | 6.83 | 0.15 | 0.25 |
| 4°C | Ffo | 24.76 | 7.15 | 0.16 | 0.24 |
| 4°C | Ffo | 26.91 | 7.28 | 0.15 | 0.26 |
| 4°C | Ffo | 25.05 | 6.97 | 0.15 | 0.25 |
| 4°C | Ffo | 25.80 | 7.11 | 0.17 | 0.27 |
| 6°C | H | 18.43 | 7.84 | 0.92 | 1.55 |
| 6°C | H | 18.57 | 6.58 | 1.29 | 1.60 |
| 6°C | H | 18.44 | 7.88 | 1.35 | 1.37 |
| 6°C | H | 19.36 | 7.48 | 1.05 | 1.80 |
| 6°C | H | 18.91 | 7.79 | 1.32 | 1.66 |
| 6°C | H | 17.31 | 7.95 | 1.05 | 1.50 |
| 6°C | H | 19.91 | 8.90 | 1.28 | 1.76 |
| 6°C | H | 17.31 | 7.85 | 1.33 | 1.66 |
| 6°C | H | 19.91 | 8.76 | 1.05 | 1.65 |
| 6°C | H | 19.36 | 8.06 | 1.40 | 1.52 |
| 6°C | H | 19.72 | 7.74 | 1.49 | 1.63 |
| 6°C | H | 19.43 | 8.19 | 1.35 | 1.65 |
| 6°C | H | 19.34 | 8.91 | 1.47 | 1.88 |
| 6°C | H | 19.06 | 7.02 | 1.20 | 1.80 |
| 6°C | H | 18.47 | 6.79 | 1.12 | 1.89 |
| 6°C | H | 19.59 | 7.78 | 1.36 | 1.84 |
| 6°C | H | 19.41 | 7.81 | 1.44 | 1.88 |
| 6°C | H | 18.95 | 8.35 | 1.12 | 1.69 |
| 6°C | H | 19.51 | 7.92 | 1.30 | 1.79 |
| 6°C | H | 19.18 | 7.53 | 1.39 | 1.71 |
| 6°C | H | 19.50 | 7.78 | 1.26 | 1.83 |
| 6°C | H | 19.44 | 7.68 | 1.43 | 1.86 |
| 6°C | H | 19.18 | 7.46 | 1.33 | 1.71 |
| 6°C | H | 19.49 | 7.11 | 1.16 | 1.68 |
| 6°C | H | 20.13 | 8.69 | 1.19 | 1.66 |
| 6°C | H | 19.40 | 7.82 | 1.20 | 1.67 |
| 6°C | H | 19.17 | 8.58 | 1.30 | 1.70 |
| 6°C | H | 19.21 | 8.17 | 1.41 | 1.81 |
| 6°C | H | 19.16 | 7.74 | 1.20 | 1.93 |
| 6°C | H | 18.67 | 8.13 | 1.40 | 1.74 |
| 6°C | H | 19.80 | 7.79 | 1.25 | 1.73 |
| 6°C | H | 19.18 | 8.17 | 1.10 | 1.92 |
| 6°C | H | 19.30 | 8.09 | 1.37 | 1.82 |
| 6°C | H | 19.26 | 7.07 | 1.19 | 1.66 |
| 6°C | H | 18.60 | 7.54 | 1.23 | 1.75 |
| 6°C | H | 19.60 | 7.71 | 1.48 | 1.91 |
| 6°C | H | 19.56 | 7.54 | 1.20 | 1.69 |
| 6°C | H | 18.74 | 7.37 | 1.06 | 1.68 |
| 6°C | H | 19.60 | 7.97 | 1.27 | 1.84 |
| 6°C | H | 18.90 | 7.59 | 1.14 | 1.74 |
| 6°C | H | 19.35 | 6.97 | 1.15 | 1.79 |
| 6°C | H | 19.81 | 8.17 | 1.21 | 1.77 |
| 6°C | H | 19.02 | 7.55 | 1.33 | 1.78 |
| 6°C | H | 19.36 | 8.48 | 1.52 | 1.66 |
| 6°C | H | 19.98 | 8.49 | 1.42 | 1.68 |
| 6°C | H | 19.30 | 8.22 | 1.41 | 1.64 |
| 6°C | H | 18.78 | 6.80 | 1.09 | 1.72 |
| 6°C | H | 18.84 | 7.30 | 1.13 | 1.78 |
| 6°C | H | 18.18 | 7.08 | 1.06 | 1.80 |
| 6°C | H | 19.48 | 7.93 | 1.42 | 1.77 |
| 6°C | H | 19.84 | 8.27 | 1.27 | 1.91 |
| 6°C | H | 18.97 | 7.33 | 1.39 | 1.80 |
| 6°C | H | 19.69 | 7.62 | 1.35 | 1.85 |
| 6°C | H | 18.57 | 7.84 | 1.39 | 1.87 |
| 6°C | H | 19.07 | 7.82 | 1.21 | 1.69 |
| 6°C | H | 19.31 | 7.93 | 1.37 | 1.84 |
| 6°C | H | 19.22 | 8.01 | 1.23 | 1.70 |
| 6°C | H | 19.44 | 7.69 | 1.48 | 1.73 |
| 6°C | H | 18.68 | 8.21 | 1.07 | 1.58 |
| 6°C | H | 20.27 | 7.87 | 1.40 | 1.82 |
| 6°C | H | 20.13 | 7.69 | 1.20 | 1.74 |
| 6°C | H | 18.79 | 7.35 | 1.33 | 1.76 |
| 6°C | H | 18.83 | 7.70 | 1.34 | 1.77 |
| 6°C | H | 19.27 | 8.15 | 1.44 | 1.80 |
| 6°C | H | 19.15 | 8.17 | 1.36 | 1.74 |
| 6°C | H | 19.39 | 7.48 | 1.25 | 1.78 |
| 6°C | H | 19.06 | 7.81 | 1.50 | 1.79 |
| 6°C | H | 19.14 | 7.87 | 1.14 | 1.58 |
| 6°C | H | 20.27 | 9.43 | 1.36 | 1.77 |
| 6°C | H | 19.79 | 8.36 | 1.16 | 1.62 |
| 6°C | H | 18.67 | 6.84 | 1.21 | 1.61 |
| 6°C | H | 18.62 | 7.42 | 1.38 | 1.64 |
| 6°C | H | 18.82 | 6.78 | 1.35 | 1.86 |
| 6°C | H | 18.70 | 7.14 | 1.39 | 1.75 |
| 6°C | H | 19.50 | 7.55 | 1.26 | 1.84 |
| 6°C | H | 19.52 | 7.87 | 1.21 | 1.79 |
| 6°C | H | 18.90 | 7.22 | 1.14 | 1.65 |
| 6°C | H | 18.66 | 7.66 | 1.20 | 1.57 |
| 6°C | H | 19.16 | 7.65 | 1.29 | 1.82 |
| 6°C | H | 19.78 | 8.21 | 1.31 | 1.80 |
| 6°C | H | 19.07 | 7.75 | 1.37 | 1.75 |
| 6°C | H | 19.69 | 7.55 | 1.16 | 1.87 |
| 6°C | H | 18.39 | 7.85 | 1.44 | 1.78 |
| 6°C | H | 18.90 | 7.76 | 1.39 | 1.76 |
| 6°C | H | 19.28 | 7.77 | 1.36 | 1.85 |
| 6°C | H | 19.52 | 7.34 | 1.45 | 1.80 |
| 6°C | H | 19.44 | 8.14 | 1.36 | 1.81 |
| 6°C | H | 19.42 | 7.69 | 1.43 | 1.72 |
| 6°C | H | 19.00 | 7.35 | 1.50 | 1.90 |
| 6°C | H | 19.62 | 7.88 | 1.36 | 1.77 |
| 6°C | H | 18.68 | 7.48 | 1.24 | 1.74 |
| 6°C | H | 19.00 | 7.82 | 1.23 | 1.61 |
| 6°C | H | 17.77 | 7.61 | 1.16 | 1.53 |
| 6°C | H | 19.96 | 8.01 | 1.23 | 1.72 |
| 6°C | H | 18.83 | 7.79 | 1.14 | 1.76 |
| 6°C | H | 19.37 | 7.67 | 1.28 | 1.68 |
| 6°C | H | 19.51 | 7.49 | 1.34 | 1.71 |
| 6°C | H | 19.32 | 8.06 | 1.15 | 1.85 |
| 6°C | H | 19.51 | 7.61 | 1.15 | 1.62 |
| 6°C | H | 18.42 | 7.93 | 1.25 | 1.59 |
| 6°C | H | 19.24 | 7.96 | 1.37 | 1.64 |
| 6°C | H | 18.06 | 7.41 | 1.05 | 1.77 |
| 6°C | H | 18.59 | 8.34 | 1.13 | 1.76 |
| 6°C | H | 19.52 | 7.65 | 1.18 | 1.80 |
| 6°C | H | 19.15 | 8.11 | 1.44 | 1.92 |
| 6°C | H | 19.38 | 6.89 | 1.46 | 1.69 |
| 6°C | H | 19.03 | 6.96 | 1.10 | 1.68 |
| 6°C | H | 19.04 | 7.85 | 1.35 | 1.78 |
| 6°C | H | 17.97 | 7.26 | 1.18 | 1.60 |
| 6°C | H | 18.49 | 8.12 | 1.43 | 1.62 |
| 6°C | H | 18.84 | 7.93 | 1.38 | 1.73 |
| 6°C | H | 18.48 | 7.93 | 1.44 | 1.93 |
| 6°C | H | 19.02 | 7.74 | 1.23 | 1.88 |
| 6°C | H | 18.83 | 7.99 | 1.44 | 1.80 |
| 6°C | H | 18.55 | 8.15 | 1.31 | 1.63 |
| 6°C | EM | 23.96 | 8.27 | 1.77 | 2.24 |
| 6°C | EM | 22.43 | 8.08 | 1.66 | 2.17 |
| 6°C | EM | 23.50 | 7.08 | 1.80 | 2.15 |
| 6°C | EM | 23.54 | 7.59 | 1.73 | 2.29 |
| 6°C | EM | 24.20 | 7.99 | 1.77 | 2.16 |
| 6°C | EM | 24.62 | 8.38 | 1.50 | 2.24 |
| 6°C | EM | 22.61 | 8.42 | 1.63 | 2.06 |
| 6°C | EM | 23.41 | 7.58 | 1.50 | 2.28 |
| 6°C | EM | 23.06 | 8.45 | 1.66 | 1.86 |
| 6°C | EM | 24.50 | 7.94 | 1.48 | 2.34 |
| 6°C | EM | 22.46 | 8.52 | 1.53 | 2.05 |
| 6°C | EM | 23.64 | 8.47 | 1.50 | 1.97 |
| 6°C | EM | 24.46 | 7.70 | 1.67 | 2.18 |
| 6°C | EM | 25.49 | 8.90 | 1.50 | 2.07 |
| 6°C | EM | 24.10 | 7.85 | 1.47 | 2.09 |
| 6°C | EM | 22.82 | 7.64 | 1.46 | 2.49 |
| 6°C | EM | 23.63 | 8.28 | 1.69 | 1.98 |
| 6°C | EM | 25.22 | 7.84 | 1.66 | 2.18 |
| 6°C | EM | 24.94 | 8.04 | 1.45 | 2.38 |
| 6°C | EM | 24.24 | 6.84 | 1.64 | 2.06 |
| 6°C | EM | 23.24 | 9.13 | 1.68 | 2.11 |
| 6°C | EM | 25.28 | 7.21 | 1.43 | 2.34 |
| 6°C | EM | 21.74 | 7.83 | 1.23 | 2.08 |
| 6°C | EM | 24.20 | 7.53 | 1.71 | 2.45 |
| 6°C | EM | 24.05 | 7.59 | 1.43 | 2.27 |
| 6°C | EM | 24.23 | 6.87 | 1.63 | 2.10 |
| 6°C | EM | 25.46 | 7.91 | 1.72 | 2.12 |
| 6°C | EM | 23.75 | 8.01 | 1.47 | 2.09 |
| 6°C | EM | 23.59 | 7.88 | 1.79 | 2.16 |
| 6°C | EM | 25.24 | 7.80 | 1.62 | 2.24 |
| 6°C | EM | 24.56 | 8.78 | 1.75 | 2.20 |
| 6°C | EM | 24.55 | 9.55 | 1.43 | 2.24 |
| 6°C | EM | 24.89 | 7.80 | 1.47 | 2.29 |
| 6°C | EM | 23.95 | 8.99 | 1.47 | 2.07 |
| 6°C | EM | 24.31 | 7.35 | 1.58 | 2.30 |
| 6°C | EM | 25.16 | 7.47 | 1.52 | 2.24 |
| 6°C | EM | 25.37 | 7.33 | 1.68 | 2.18 |
| 6°C | EM | 24.79 | 8.70 | 1.47 | 2.30 |
| 6°C | EM | 25.00 | 7.64 | 1.72 | 2.22 |
| 6°C | EM | 25.48 | 8.77 | 1.50 | 2.29 |
| 6°C | EM | 22.23 | 7.77 | 1.48 | 1.89 |
| 6°C | EM | 23.48 | 6.92 | 1.51 | 2.26 |
| 6°C | EM | 23.91 | 7.89 | 1.63 | 2.13 |
| 6°C | EM | 24.15 | 7.25 | 1.50 | 2.31 |
| 6°C | EM | 21.87 | 7.99 | 1.39 | 1.93 |
| 6°C | EM | 23.50 | 8.18 | 1.71 | 1.92 |
| 6°C | EM | 21.85 | 7.46 | 1.61 | 1.81 |
| 6°C | EM | 23.74 | 8.75 | 1.48 | 2.14 |
| 6°C | EM | 23.36 | 8.03 | 1.30 | 2.22 |
| 6°C | EM | 25.95 | 7.79 | 1.80 | 2.36 |
| 6°C | EM | 24.97 | 8.48 | 1.59 | 2.34 |
| 6°C | EM | 23.30 | 8.48 | 1.69 | 1.87 |
| 6°C | EM | 23.54 | 6.47 | 1.69 | 1.91 |
| 6°C | EM | 23.59 | 7.26 | 1.67 | 1.92 |
| 6°C | EM | 24.59 | 7.96 | 1.48 | 2.30 |
| 6°C | EM | 23.10 | 7.86 | 1.32 | 2.07 |
| 6°C | EM | 23.25 | 8.06 | 1.45 | 1.69 |
| 6°C | EM | 23.33 | 7.96 | 1.73 | 1.93 |
| 6°C | EM | 23.02 | 8.46 | 1.28 | 1.50 |
| 6°C | EM | 25.01 | 7.67 | 1.72 | 2.16 |
| 6°C | EM | 23.44 | 8.52 | 1.42 | 2.03 |
| 6°C | EM | 23.45 | 7.94 | 1.62 | 1.77 |
| 6°C | EM | 22.87 | 7.72 | 1.41 | 2.08 |
| 6°C | EM | 23.14 | 8.61 | 1.48 | 2.16 |
| 6°C | EM | 23.41 | 8.87 | 1.68 | 1.86 |
| 6°C | EM | 24.42 | 8.16 | 1.54 | 2.27 |
| 6°C | EM | 24.80 | 8.00 | 1.77 | 2.10 |
| 6°C | EM | 24.84 | 7.56 | 1.65 | 2.23 |
| 6°C | EM | 23.17 | 8.24 | 1.43 | 1.71 |
| 6°C | EM | 22.40 | 6.58 | 1.62 | 1.57 |
| 6°C | EM | 23.94 | 7.35 | 1.43 | 2.08 |
| 6°C | EM | 24.08 | 7.35 | 1.42 | 2.23 |
| 6°C | EM | 24.15 | 7.13 | 1.72 | 2.40 |
| 6°C | EM | 24.56 | 7.77 | 1.76 | 2.38 |
| 6°C | EM | 23.11 | 7.98 | 1.69 | 2.18 |
| 6°C | EM | 24.75 | 7.40 | 1.49 | 2.32 |
| 6°C | EM | 24.92 | 7.32 | 1.63 | 2.21 |
| 6°C | EM | 23.67 | 6.88 | 1.68 | 1.64 |
| 6°C | EM | 25.33 | 7.25 | 1.50 | 2.29 |
| 6°C | EM | 24.09 | 9.01 | 1.69 | 2.14 |
| 6°C | EM | 23.72 | 7.67 | 1.50 | 2.20 |
| 6°C | EM | 23.68 | 8.27 | 1.69 | 2.28 |
| 6°C | EM | 23.67 | 7.77 | 1.71 | 2.26 |
| 6°C | EM | 24.81 | 8.55 | 1.70 | 2.24 |
| 6°C | EM | 23.43 | 8.59 | 1.43 | 1.76 |
| 6°C | EM | 24.41 | 7.60 | 1.39 | 2.06 |
| 6°C | EM | 24.71 | 7.70 | 1.71 | 2.33 |
| 6°C | EM | 23.53 | 8.23 | 1.34 | 2.10 |
| 6°C | EM | 24.58 | 7.34 | 1.45 | 1.87 |
| 6°C | EM | 23.94 | 7.45 | 1.33 | 2.00 |
| 6°C | EM | 24.66 | 7.02 | 1.69 | 2.23 |
| 6°C | EM | 24.59 | 7.08 | 1.73 | 2.13 |
| 6°C | EM | 24.30 | 8.52 | 1.43 | 1.89 |
| 6°C | EM | 22.82 | 8.09 | 1.45 | 2.03 |
| 6°C | EM | 24.72 | 6.97 | 1.42 | 2.22 |
| 6°C | EM | 23.81 | 8.14 | 1.35 | 2.09 |
| 6°C | EM | 24.09 | 8.22 | 1.72 | 2.12 |
| 6°C | EM | 23.28 | 8.37 | 1.68 | 2.09 |
| 6°C | EM | 24.32 | 8.12 | 1.73 | 2.24 |
| 6°C | EM | 23.95 | 7.57 | 1.77 | 2.14 |
| 6°C | Ffo | 25.09 | 8.35 | 1.69 | 2.68 |
| 6°C | Ffo | 24.17 | 7.03 | 1.41 | 2.27 |
| 6°C | Ffo | 24.31 | 6.82 | 1.55 | 2.51 |
| 6°C | Ffo | 24.46 | 7.27 | 1.76 | 2.41 |
| 6°C | Ffo | 24.59 | 6.87 | 1.43 | 2.32 |
| 6°C | Ffo | 25.44 | 6.35 | 1.65 | 2.52 |
| 6°C | Ffo | 25.73 | 6.94 | 1.72 | 2.53 |
| 6°C | Ffo | 25.49 | 6.70 | 1.51 | 2.39 |
| 6°C | Ffo | 25.62 | 6.44 | 1.63 | 2.58 |
| 6°C | Ffo | 25.69 | 6.68 | 1.48 | 2.56 |
| 6°C | Ffo | 25.45 | 7.36 | 1.50 | 2.30 |
| 6°C | Ffo | 25.31 | 6.79 | 1.56 | 2.33 |
| 6°C | Ffo | 26.18 | 6.72 | 1.88 | 2.53 |
| 6°C | Ffo | 26.44 | 6.93 | 1.45 | 2.55 |
| 6°C | Ffo | 25.09 | 8.35 | 1.77 | 2.68 |
| 6°C | Ffo | 24.63 | 6.48 | 1.53 | 2.55 |
| 6°C | Ffo | 24.29 | 7.24 | 1.57 | 2.61 |
| 6°C | Ffo | 24.91 | 7.08 | 1.74 | 2.59 |
| 6°C | Ffo | 25.22 | 6.66 | 1.37 | 2.59 |
| 6°C | Ffo | 21.50 | 6.62 | 1.71 | 2.14 |
| 6°C | Ffo | 24.00 | 6.01 | 1.30 | 2.35 |
| 6°C | Ffo | 23.94 | 7.00 | 1.65 | 2.37 |
| 6°C | Ffo | 25.72 | 7.16 | 1.75 | 2.58 |
| 6°C | Ffo | 24.85 | 6.30 | 1.51 | 2.54 |
| 6°C | Ffo | 25.80 | 6.85 | 1.56 | 2.64 |
| 6°C | Ffo | 26.12 | 6.63 | 1.79 | 2.72 |
| 6°C | Ffo | 23.96 | 6.78 | 1.59 | 2.38 |
| 6°C | Ffo | 24.41 | 7.04 | 1.59 | 2.21 |
| 6°C | Ffo | 24.16 | 7.34 | 1.60 | 2.13 |
| 6°C | Ffo | 23.88 | 7.28 | 1.48 | 2.29 |
| 6°C | Ffo | 23.56 | 7.27 | 1.76 | 2.29 |
| 6°C | Ffo | 23.29 | 6.70 | 1.90 | 2.45 |
| 6°C | Ffo | 25.14 | 6.81 | 1.69 | 2.57 |
| 6°C | Ffo | 25.06 | 7.09 | 1.57 | 2.34 |
| 6°C | Ffo | 25.93 | 7.26 | 1.43 | 2.45 |
| 6°C | Ffo | 25.11 | 6.35 | 1.38 | 2.40 |
| 6°C | Ffo | 25.12 | 6.74 | 1.61 | 2.37 |
| 6°C | Ffo | 25.51 | 6.76 | 1.49 | 2.53 |
| 6°C | Ffo | 24.28 | 7.08 | 1.85 | 2.22 |
| 6°C | Ffo | 25.74 | 7.87 | 1.51 | 2.48 |
| 6°C | Ffo | 25.33 | 6.85 | 1.57 | 2.41 |
| 6°C | Ffo | 25.31 | 6.66 | 1.63 | 2.53 |
| 6°C | Ffo | 25.29 | 9.80 | 1.80 | 2.66 |
| 6°C | Ffo | 24.03 | 6.22 | 1.45 | 2.52 |
| 6°C | Ffo | 24.73 | 6.37 | 1.47 | 2.51 |
| 6°C | Ffo | 24.39 | 7.08 | 1.61 | 2.55 |
| 6°C | Ffo | 23.23 | 7.24 | 1.52 | 2.36 |
| 6°C | Ffo | 24.27 | 6.50 | 1.80 | 2.40 |
| 6°C | Ffo | 24.76 | 6.87 | 1.66 | 2.69 |
| 6°C | Ffo | 24.48 | 6.78 | 1.47 | 2.54 |
| 6°C | Ffo | 26.56 | 6.82 | 1.75 | 2.39 |
| 6°C | Ffo | 25.49 | 6.77 | 1.40 | 2.58 |
| 6°C | Ffo | 22.91 | 6.77 | 1.34 | 2.35 |
| 6°C | Ffo | 23.80 | 7.30 | 1.51 | 2.34 |
| 6°C | Ffo | 25.75 | 7.00 | 1.82 | 2.48 |
| 6°C | Ffo | 25.85 | 6.85 | 1.48 | 2.58 |
| 6°C | Ffo | 25.61 | 6.87 | 1.71 | 2.55 |
| 6°C | Ffo | 24.29 | 6.84 | 1.64 | 2.43 |
| 6°C | Ffo | 25.36 | 7.75 | 1.69 | 2.50 |
| 6°C | Ffo | 24.13 | 6.82 | 1.49 | 2.43 |
| 6°C | Ffo | 23.48 | 6.81 | 1.46 | 2.50 |
| 6°C | Ffo | 24.29 | 6.90 | 1.50 | 2.57 |
| 6°C | Ffo | 26.27 | 7.29 | 1.51 | 2.40 |
| 6°C | Ffo | 24.82 | 7.09 | 1.44 | 2.48 |
| 6°C | Ffo | 26.04 | 6.45 | 1.48 | 2.52 |
| 6°C | Ffo | 23.54 | 7.06 | 1.45 | 2.21 |
| 6°C | Ffo | 24.21 | 7.43 | 1.78 | 2.45 |
| 6°C | Ffo | 25.49 | 6.92 | 1.80 | 2.69 |
| 6°C | Ffo | 25.49 | 7.12 | 1.62 | 2.52 |
| 6°C | Ffo | 24.80 | 7.30 | 1.56 | 2.49 |
| 6°C | Ffo | 24.49 | 6.64 | 1.67 | 2.56 |
| 6°C | Ffo | 24.77 | 6.56 | 1.51 | 2.30 |
| 6°C | Ffo | 24.58 | 6.40 | 1.71 | 2.50 |
| 6°C | Ffo | 24.67 | 7.37 | 1.77 | 2.46 |
| 6°C | Ffo | 24.72 | 6.91 | 1.60 | 2.44 |
| 6°C | Ffo | 24.54 | 6.70 | 1.82 | 2.65 |
| 6°C | Ffo | 24.45 | 6.41 | 1.56 | 2.49 |
| 6°C | Ffo | 24.67 | 6.55 | 1.73 | 2.56 |
| 6°C | Ffo | 24.63 | 7.09 | 1.63 | 2.61 |
| 6°C | Ffo | 24.44 | 7.29 | 1.80 | 2.47 |
| 6°C | Ffo | 25.38 | 6.63 | 1.52 | 2.44 |
| 6°C | Ffo | 23.93 | 6.87 | 1.70 | 2.28 |
| 6°C | Ffo | 24.70 | 6.63 | 1.37 | 2.44 |
| 6°C | Ffo | 23.91 | 8.14 | 1.48 | 2.49 |
| 6°C | Ffo | 24.33 | 8.39 | 1.50 | 2.37 |
| 6°C | Ffo | 25.97 | 8.71 | 1.59 | 2.38 |
| 6°C | Ffo | 25.28 | 8.52 | 1.52 | 2.51 |
| 6°C | Ffo | 23.93 | 6.87 | 1.40 | 2.28 |
| 6°C | Ffo | 24.73 | 7.69 | 1.57 | 2.36 |
| 6°C | Ffo | 25.95 | 7.89 | 1.76 | 2.58 |
| 6°C | Ffo | 25.99 | 7.46 | 1.59 | 2.62 |
| 6°C | Ffo | 25.53 | 8.64 | 1.66 | 2.54 |
| 6°C | Ffo | 24.85 | 8.22 | 1.43 | 2.30 |
| 6°C | Ffo | 24.99 | 7.76 | 1.85 | 2.50 |
| 6°C | Ffo | 25.20 | 7.13 | 1.66 | 2.54 |
| 6°C | Ffo | 25.63 | 7.61 | 1.79 | 2.46 |
| 6°C | Ffo | 25.29 | 7.82 | 1.30 | 2.39 |
| 6°C | Ffo | 23.61 | 8.30 | 1.17 | 2.29 |
| 6°C | Ffo | 24.74 | 7.85 | 1.50 | 2.25 |
| 6°C | Ffo | 24.04 | 7.76 | 1.89 | 2.29 |
| 6°C | Ffo | 23.96 | 7.72 | 1.58 | 2.16 |
| 6°C | Ffo | 25.87 | 7.50 | 1.79 | 2.58 |
| 6°C | Ffo | 25.17 | 6.50 | 1.31 | 2.18 |
| 6°C | Ffo | 25.46 | 7.72 | 1.49 | 2.29 |
| 6°C | Ffo | 24.89 | 7.07 | 1.56 | 2.40 |
| 6°C | Ffo | 25.27 | 8.31 | 1.49 | 2.63 |
| 6°C | Ffo | 24.98 | 7.25 | 1.44 | 2.26 |
| 6°C | Ffo | 25.19 | 8.05 | 1.37 | 2.64 |
| 6°C | Ffo | 24.16 | 7.99 | 1.81 | 2.28 |
| 6°C | Ffo | 25.17 | 7.43 | 1.43 | 2.67 |
| 6°C | Ffo | 25.18 | 8.30 | 1.28 | 2.47 |
| 6°C | Ffo | 23.06 | 8.26 | 0.97 | 2.07 |
| 8°C | H | 17.99 | 7.91 | 1.01 | 1.62 |
| 8°C | H | 18.63 | 8.31 | 1.38 | 1.67 |
| 8°C | H | 18.69 | 7.89 | 1.32 | 1.64 |
| 8°C | H | 18.60 | 7.87 | 1.14 | 1.53 |
| 8°C | H | 19.01 | 8.37 | 1.12 | 1.61 |
| 8°C | H | 18.67 | 7.91 | 1.31 | 1.53 |
| 8°C | H | 18.75 | 7.77 | 1.31 | 1.55 |
| 8°C | H | 18.55 | 8.26 | 1.39 | 1.60 |
| 8°C | H | 19.17 | 8.38 | 1.04 | 1.73 |
| 8°C | H | 18.38 | 7.77 | 1.12 | 1.70 |
| 8°C | H | 18.98 | 8.43 | 1.26 | 1.64 |
| 8°C | H | 18.24 | 8.28 | 1.17 | 1.58 |
| 8°C | H | 19.55 | 8.41 | 1.37 | 1.68 |
| 8°C | H | 19.19 | 8.93 | 1.12 | 1.67 |
| 8°C | H | 19.19 | 9.03 | 1.32 | 1.34 |
| 8°C | H | 19.17 | 8.56 | 1.37 | 1.58 |
| 8°C | H | 18.15 | 6.81 | 1.05 | 1.47 |
| 8°C | H | 18.42 | 7.38 | 1.28 | 1.75 |
| 8°C | H | 18.76 | 7.18 | 1.27 | 1.43 |
| 8°C | H | 18.58 | 7.19 | 1.34 | 1.76 |
| 8°C | H | 18.63 | 7.24 | 1.34 | 1.70 |
| 8°C | H | 18.11 | 7.44 | 1.32 | 1.66 |
| 8°C | H | 18.90 | 7.44 | 1.21 | 1.64 |
| 8°C | H | 17.62 | 7.36 | 1.06 | 1.65 |
| 8°C | H | 16.88 | 6.82 | 0.94 | 1.26 |
| 8°C | H | 18.19 | 7.88 | 1.19 | 1.69 |
| 8°C | H | 18.37 | 7.61 | 1.00 | 1.71 |
| 8°C | H | 18.34 | 7.74 | 1.26 | 1.63 |
| 8°C | H | 18.73 | 8.16 | 1.01 | 1.54 |
| 8°C | H | 16.53 | 6.37 | 0.89 | 1.37 |
| 8°C | H | 18.66 | 7.35 | 1.00 | 1.56 |
| 8°C | H | 18.69 | 8.37 | 1.21 | 1.73 |
| 8°C | H | 17.71 | 6.35 | 1.23 | 1.63 |
| 8°C | H | 17.33 | 6.20 | 1.14 | 1.27 |
| 8°C | H | 16.86 | 6.55 | 1.14 | 1.44 |
| 8°C | H | 17.87 | 7.29 | 1.20 | 1.71 |
| 8°C | H | 17.98 | 7.26 | 1.22 | 1.67 |
| 8°C | H | 18.00 | 7.42 | 1.27 | 1.50 |
| 8°C | H | 18.75 | 7.61 | 1.28 | 1.28 |
| 8°C | H | 18.98 | 7.75 | 1.37 | 1.67 |
| 8°C | H | 18.84 | 7.76 | 0.84 | 1.60 |
| 8°C | H | 18.33 | 7.34 | 1.23 | 1.23 |
| 8°C | H | 18.84 | 7.47 | 1.20 | 1.58 |
| 8°C | H | 18.40 | 7.83 | 1.33 | 1.67 |
| 8°C | H | 18.30 | 7.87 | 1.23 | 1.71 |
| 8°C | H | 18.01 | 7.77 | 1.12 | 1.77 |
| 8°C | H | 17.93 | 8.41 | 1.02 | 1.77 |
| 8°C | H | 17.43 | 7.17 | 1.04 | 1.52 |
| 8°C | H | 17.78 | 7.70 | 1.26 | 1.60 |
| 8°C | H | 17.98 | 7.75 | 1.22 | 1.63 |
| 8°C | H | 17.86 | 7.98 | 1.28 | 1.59 |
| 8°C | H | 17.83 | 6.88 | 1.17 | 1.66 |
| 8°C | H | 19.03 | 7.47 | 1.35 | 1.70 |
| 8°C | H | 17.70 | 7.60 | 1.22 | 1.41 |
| 8°C | H | 18.64 | 7.62 | 1.11 | 1.79 |
| 8°C | H | 18.73 | 8.26 | 1.36 | 1.65 |
| 8°C | H | 18.75 | 9.06 | 1.27 | 1.37 |
| 8°C | H | 17.76 | 7.53 | 1.27 | 1.57 |
| 8°C | H | 17.66 | 7.82 | 0.96 | 1.57 |
| 8°C | H | 18.96 | 8.33 | 1.26 | 1.60 |
| 8°C | H | 18.40 | 7.61 | 1.28 | 1.66 |
| 8°C | H | 18.98 | 8.29 | 1.42 | 1.64 |
| 8°C | H | 18.95 | 7.76 | 1.11 | 1.65 |
| 8°C | H | 17.29 | 6.62 | 1.15 | 1.60 |
| 8°C | H | 17.73 | 6.71 | 1.17 | 1.67 |
| 8°C | H | 18.17 | 7.61 | 1.03 | 1.68 |
| 8°C | H | 18.09 | 7.22 | 1.25 | 1.72 |
| 8°C | H | 19.38 | 7.70 | 1.39 | 1.78 |
| 8°C | H | 18.57 | 7.41 | 1.20 | 1.72 |
| 8°C | H | 18.30 | 7.83 | 1.24 | 1.83 |
| 8°C | H | 17.82 | 7.19 | 1.08 | 1.74 |
| 8°C | H | 18.58 | 7.67 | 1.32 | 1.76 |
| 8°C | H | 18.28 | 7.25 | 1.19 | 1.56 |
| 8°C | H | 16.35 | 6.70 | 0.91 | 1.60 |
| 8°C | H | 18.54 | 7.53 | 1.37 | 1.88 |
| 8°C | H | 18.53 | 7.85 | 1.10 | 1.68 |
| 8°C | H | 19.41 | 8.63 | 1.43 | 1.68 |
| 8°C | H | 17.96 | 7.54 | 1.13 | 1.88 |
| 8°C | H | 18.08 | 7.13 | 1.30 | 1.49 |
| 8°C | H | 18.22 | 7.90 | 1.36 | 1.69 |
| 8°C | H | 18.09 | 8.03 | 1.24 | 1.39 |
| 8°C | H | 18.02 | 7.43 | 1.21 | 1.69 |
| 8°C | H | 18.46 | 6.85 | 1.27 | 1.59 |
| 8°C | H | 18.43 | 7.35 | 1.37 | 1.64 |
| 8°C | H | 18.01 | 7.64 | 1.19 | 1.78 |
| 8°C | H | 18.63 | 7.99 | 1.40 | 1.96 |
| 8°C | H | 18.24 | 6.94 | 1.39 | 1.66 |
| 8°C | H | 17.86 | 7.52 | 1.41 | 1.83 |
| 8°C | H | 19.47 | 7.56 | 1.34 | 1.61 |
| 8°C | H | 18.32 | 7.75 | 1.36 | 1.81 |
| 8°C | H | 17.67 | 7.70 | 1.31 | 1.79 |
| 8°C | H | 17.64 | 7.77 | 1.29 | 1.88 |
| 8°C | H | 17.91 | 7.73 | 1.24 | 1.78 |
| 8°C | H | 17.93 | 7.42 | 1.14 | 1.83 |
| 8°C | H | 18.23 | 7.88 | 1.25 | 1.75 |
| 8°C | H | 19.41 | 6.67 | 1.19 | 1.82 |
| 8°C | H | 17.90 | 6.84 | 1.43 | 1.62 |
| 8°C | H | 17.91 | 7.18 | 1.16 | 1.62 |
| 8°C | H | 17.67 | 7.74 | 1.14 | 1.65 |
| 8°C | H | 17.04 | 7.09 | 1.18 | 1.77 |
| 8°C | H | 18.47 | 7.56 | 1.23 | 1.65 |
| 8°C | H | 17.68 | 7.51 | 1.27 | 2.00 |
| 8°C | H | 19.56 | 7.41 | 1.37 | 1.75 |
| 8°C | H | 18.11 | 8.45 | 1.11 | 1.82 |
| 8°C | H | 18.75 | 7.35 | 1.46 | 1.78 |
| 8°C | H | 17.90 | 8.82 | 1.35 | 1.98 |
| 8°C | H | 18.81 | 8.03 | 1.27 | 1.65 |
| 8°C | H | 17.85 | 7.05 | 1.12 | 1.68 |
| 8°C | H | 18.01 | 7.17 | 1.22 | 1.58 |
| 8°C | H | 17.78 | 7.23 | 1.35 | 1.62 |
| 8°C | H | 17.59 | 7.70 | 1.27 | 1.70 |
| 8°C | H | 17.96 | 7.02 | 1.05 | 1.72 |
| 8°C | H | 17.62 | 7.25 | 1.13 | 1.64 |
| 8°C | H | 18.17 | 7.13 | 1.21 | 1.82 |
| 8°C | H | 17.50 | 8.06 | 1.21 | 1.58 |
| 8°C | H | 17.02 | 7.33 | 1.19 | 1.63 |
| 8°C | H | 18.20 | 7.43 | 1.23 | 1.86 |
| 8°C | H | 17.47 | 8.26 | 1.21 | 1.51 |
| 8°C | H | 16.57 | 6.42 | 1.09 | 1.60 |
| 8°C | H | 17.31 | 7.77 | 1.19 | 1.56 |
| 8°C | H | 17.52 | 7.10 | 1.17 | 1.68 |
| 8°C | H | 19.12 | 7.74 | 1.32 | 1.78 |
| 8°C | H | 18.62 | 7.94 | 1.39 | 2.06 |
| 8°C | EM | 25.22 | 6.44 | 1.79 | 2.24 |
| 8°C | EM | 24.82 | 7.78 | 1.55 | 2.36 |
| 8°C | EM | 24.74 | 7.28 | 1.66 | 2.23 |
| 8°C | EM | 25.11 | 7.08 | 1.81 | 2.41 |
| 8°C | EM | 26.21 | 7.68 | 1.76 | 2.38 |
| 8°C | EM | 26.28 | 7.60 | 1.69 | 2.10 |
| 8°C | EM | 25.00 | 8.32 | 1.69 | 2.20 |
| 8°C | EM | 25.10 | 7.42 | 1.80 | 2.23 |
| 8°C | EM | 26.31 | 7.45 | 1.74 | 2.42 |
| 8°C | EM | 25.67 | 8.27 | 1.74 | 2.45 |
| 8°C | EM | 25.90 | 7.40 | 1.69 | 2.10 |
| 8°C | EM | 24.36 | 7.77 | 1.73 | 2.11 |
| 8°C | EM | 24.87 | 8.64 | 1.68 | 2.27 |
| 8°C | EM | 24.89 | 7.86 | 1.79 | 2.05 |
| 8°C | EM | 23.79 | 8.67 | 1.40 | 2.20 |
| 8°C | EM | 24.52 | 8.55 | 1.91 | 2.13 |
| 8°C | EM | 24.74 | 8.77 | 1.76 | 2.30 |
| 8°C | EM | 24.60 | 8.92 | 1.53 | 2.29 |
| 8°C | EM | 25.40 | 7.65 | 1.71 | 2.20 |
| 8°C | EM | 24.83 | 7.75 | 1.77 | 2.33 |
| 8°C | EM | 24.90 | 7.57 | 1.89 | 2.43 |
| 8°C | EM | 24.70 | 8.10 | 1.72 | 2.07 |
| 8°C | EM | 24.42 | 7.38 | 1.47 | 2.37 |
| 8°C | EM | 24.51 | 7.25 | 1.66 | 2.29 |
| 8°C | EM | 24.22 | 7.56 | 1.72 | 2.13 |
| 8°C | EM | 23.74 | 8.35 | 1.63 | 2.04 |
| 8°C | EM | 23.74 | 9.13 | 1.77 | 2.25 |
| 8°C | EM | 24.58 | 7.37 | 1.71 | 2.30 |
| 8°C | EM | 25.48 | 7.07 | 1.71 | 2.30 |
| 8°C | EM | 24.68 | 7.36 | 1.72 | 2.26 |
| 8°C | EM | 24.75 | 7.12 | 1.67 | 2.10 |
| 8°C | EM | 23.87 | 8.51 | 1.52 | 2.39 |
| 8°C | EM | 25.25 | 7.17 | 1.87 | 2.20 |
| 8°C | EM | 24.02 | 7.61 | 1.63 | 2.15 |
| 8°C | EM | 24.75 | 7.44 | 1.53 | 2.16 |
| 8°C | EM | 24.21 | 8.24 | 1.53 | 2.32 |
| 8°C | EM | 24.71 | 7.72 | 1.95 | 2.19 |
| 8°C | EM | 23.95 | 7.42 | 1.80 | 2.08 |
| 8°C | EM | 23.60 | 8.02 | 1.76 | 2.21 |
| 8°C | EM | 25.13 | 7.84 | 1.45 | 2.15 |
| 8°C | EM | 24.29 | 7.65 | 1.78 | 2.43 |
| 8°C | EM | 26.13 | 7.39 | 1.72 | 2.24 |
| 8°C | EM | 25.43 | 6.81 | 1.75 | 2.24 |
| 8°C | EM | 25.12 | 7.43 | 1.47 | 2.20 |
| 8°C | EM | 24.20 | 7.95 | 1.69 | 2.06 |
| 8°C | EM | 24.08 | 8.84 | 1.83 | 2.14 |
| 8°C | EM | 23.94 | 7.77 | 1.62 | 1.99 |
| 8°C | EM | 23.92 | 7.64 | 1.54 | 2.24 |
| 8°C | EM | 24.00 | 8.13 | 1.71 | 2.08 |
| 8°C | EM | 24.95 | 6.14 | 1.48 | 2.19 |
| 8°C | EM | 27.72 | 6.28 | 1.40 | 2.17 |
| 8°C | EM | 25.87 | 6.86 | 1.41 | 1.98 |
| 8°C | EM | 25.25 | 6.39 | 1.72 | 2.26 |
| 8°C | EM | 25.07 | 7.18 | 1.35 | 2.05 |
| 8°C | EM | 23.81 | 7.18 | 1.19 | 2.01 |
| 8°C | EM | 24.78 | 6.15 | 1.72 | 2.17 |
| 8°C | EM | 24.60 | 6.61 | 1.36 | 2.21 |
| 8°C | EM | 25.26 | 6.63 | 1.71 | 1.86 |
| 8°C | EM | 27.21 | 6.63 | 1.67 | 2.38 |
| 8°C | EM | 25.03 | 7.35 | 1.74 | 2.09 |
| 8°C | EM | 24.01 | 8.31 | 1.71 | 2.03 |
| 8°C | EM | 24.79 | 7.54 | 1.47 | 2.38 |
| 8°C | EM | 24.65 | 7.06 | 1.80 | 2.34 |
| 8°C | EM | 25.34 | 7.52 | 1.72 | 2.18 |
| 8°C | EM | 24.28 | 7.85 | 1.74 | 2.03 |
| 8°C | EM | 23.94 | 8.03 | 1.43 | 2.22 |
| 8°C | EM | 25.31 | 7.02 | 1.38 | 2.11 |
| 8°C | EM | 25.87 | 6.94 | 1.48 | 2.09 |
| 8°C | EM | 24.34 | 6.99 | 1.56 | 2.17 |
| 8°C | EM | 23.94 | 8.36 | 1.73 | 2.21 |
| 8°C | EM | 24.11 | 8.52 | 1.55 | 2.18 |
| 8°C | EM | 23.63 | 7.24 | 1.37 | 2.04 |
| 8°C | EM | 23.54 | 8.05 | 1.73 | 2.30 |
| 8°C | EM | 25.07 | 7.04 | 1.69 | 2.13 |
| 8°C | EM | 24.10 | 7.80 | 1.78 | 2.45 |
| 8°C | EM | 25.20 | 7.30 | 1.45 | 2.31 |
| 8°C | EM | 25.49 | 6.93 | 1.58 | 2.19 |
| 8°C | EM | 24.31 | 7.88 | 1.49 | 2.06 |
| 8°C | EM | 24.60 | 7.74 | 1.84 | 2.32 |
| 8°C | EM | 25.37 | 7.40 | 1.54 | 2.05 |
| 8°C | EM | 24.57 | 8.03 | 1.53 | 2.04 |
| 8°C | EM | 23.87 | 7.55 | 1.79 | 2.40 |
| 8°C | EM | 25.29 | 7.24 | 1.63 | 2.16 |
| 8°C | EM | 24.26 | 7.84 | 1.81 | 2.39 |
| 8°C | EM | 24.46 | 7.28 | 1.84 | 2.20 |
| 8°C | EM | 24.90 | 6.87 | 1.51 | 2.33 |
| 8°C | EM | 25.81 | 6.76 | 1.50 | 2.13 |
| 8°C | EM | 24.16 | 6.56 | 1.50 | 2.16 |
| 8°C | EM | 25.20 | 7.57 | 1.79 | 2.30 |
| 8°C | EM | 25.19 | 7.35 | 1.51 | 2.20 |
| 8°C | EM | 25.18 | 7.08 | 1.48 | 2.42 |
| 8°C | EM | 26.07 | 7.13 | 1.85 | 2.26 |
| 8°C | EM | 24.49 | 8.11 | 1.85 | 2.31 |
| 8°C | EM | 25.27 | 7.66 | 1.80 | 2.22 |
| 8°C | EM | 25.58 | 6.98 | 1.45 | 2.19 |
| 8°C | EM | 24.53 | 7.84 | 1.50 | 2.26 |
| 8°C | EM | 25.68 | 7.81 | 1.44 | 2.33 |
| 8°C | Ffo | 24.53 | 6.83 | 1.53 | 2.46 |
| 8°C | Ffo | 24.29 | 6.66 | 1.57 | 2.26 |
| 8°C | Ffo | 23.88 | 6.77 | 1.50 | 2.43 |
| 8°C | Ffo | 24.18 | 7.21 | 1.83 | 2.37 |
| 8°C | Ffo | 24.67 | 6.84 | 1.44 | 2.29 |
| 8°C | Ffo | 24.92 | 6.43 | 1.57 | 2.62 |
| 8°C | Ffo | 26.27 | 7.05 | 1.68 | 2.54 |
| 8°C | Ffo | 24.46 | 6.60 | 1.50 | 2.35 |
| 8°C | Ffo | 26.12 | 6.24 | 1.50 | 2.57 |
| 8°C | Ffo | 25.67 | 6.71 | 1.59 | 2.52 |
| 8°C | Ffo | 24.74 | 7.27 | 1.84 | 2.26 |
| 8°C | Ffo | 24.98 | 6.88 | 1.57 | 2.30 |
| 8°C | Ffo | 26.11 | 6.66 | 1.87 | 2.43 |
| 8°C | Ffo | 26.03 | 6.96 | 1.52 | 2.32 |
| 8°C | Ffo | 24.61 | 6.57 | 1.52 | 2.38 |
| 8°C | Ffo | 24.53 | 7.23 | 1.55 | 2.50 |
| 8°C | Ffo | 24.53 | 7.13 | 1.45 | 2.54 |
| 8°C | Ffo | 25.10 | 6.36 | 1.65 | 2.53 |
| 8°C | Ffo | 21.53 | 6.65 | 1.75 | 1.83 |
| 8°C | Ffo | 23.68 | 5.98 | 1.48 | 2.26 |
| 8°C | Ffo | 23.74 | 6.80 | 1.55 | 2.25 |
| 8°C | Ffo | 25.06 | 6.97 | 1.52 | 2.57 |
| 8°C | Ffo | 24.75 | 6.41 | 1.47 | 2.26 |
| 8°C | Ffo | 25.76 | 6.72 | 1.42 | 2.59 |
| 8°C | Ffo | 26.42 | 6.68 | 1.45 | 2.58 |
| 8°C | Ffo | 23.61 | 7.37 | 1.90 | 2.01 |
| 8°C | Ffo | 23.48 | 6.67 | 1.73 | 2.43 |
| 8°C | Ffo | 23.39 | 6.99 | 1.67 | 2.42 |
| 8°C | Ffo | 23.70 | 7.43 | 1.84 | 2.27 |
| 8°C | Ffo | 23.25 | 6.56 | 1.58 | 2.37 |
| 8°C | Ffo | 24.58 | 6.79 | 1.64 | 2.57 |
| 8°C | Ffo | 25.18 | 7.25 | 1.51 | 2.41 |
| 8°C | Ffo | 23.87 | 7.04 | 1.61 | 2.07 |
| 8°C | Ffo | 26.53 | 7.10 | 1.56 | 2.42 |
| 8°C | Ffo | 25.32 | 6.06 | 1.41 | 2.37 |
| 8°C | Ffo | 24.81 | 6.74 | 1.53 | 2.23 |
| 8°C | Ffo | 25.82 | 6.63 | 1.43 | 2.40 |
| 8°C | Ffo | 24.33 | 6.91 | 1.62 | 2.26 |
| 8°C | Ffo | 26.19 | 8.01 | 1.50 | 2.40 |
| 8°C | Ffo | 24.91 | 6.52 | 1.50 | 2.29 |
| 8°C | Ffo | 24.29 | 6.30 | 1.43 | 2.43 |
| 8°C | Ffo | 23.99 | 6.51 | 1.68 | 2.54 |
| 8°C | Ffo | 23.79 | 7.49 | 1.74 | 2.39 |
| 8°C | Ffo | 23.42 | 7.36 | 1.45 | 2.26 |
| 8°C | Ffo | 24.52 | 6.43 | 2.03 | 2.28 |
| 8°C | Ffo | 24.99 | 6.97 | 1.66 | 2.52 |
| 8°C | Ffo | 25.57 | 6.76 | 1.63 | 2.46 |
| 8°C | Ffo | 26.11 | 6.91 | 1.71 | 2.25 |
| 8°C | Ffo | 25.05 | 6.52 | 1.43 | 2.69 |
| 8°C | Ffo | 23.33 | 6.66 | 1.59 | 2.30 |
| 8°C | Ffo | 23.54 | 7.39 | 1.45 | 2.10 |
| 8°C | Ffo | 25.47 | 6.92 | 1.43 | 2.25 |
| 8°C | Ffo | 25.64 | 7.03 | 1.53 | 2.43 |
| 8°C | Ffo | 25.40 | 7.58 | 1.54 | 2.48 |
| 8°C | Ffo | 24.70 | 6.89 | 1.64 | 2.44 |
| 8°C | Ffo | 24.68 | 6.83 | 1.61 | 2.18 |
| 8°C | Ffo | 24.09 | 6.66 | 1.47 | 2.39 |
| 8°C | Ffo | 23.63 | 6.66 | 1.66 | 2.36 |
| 8°C | Ffo | 24.06 | 6.89 | 1.60 | 2.50 |
| 8°C | Ffo | 26.04 | 7.39 | 1.66 | 2.49 |
| 8°C | Ffo | 25.28 | 7.18 | 1.51 | 2.27 |
| 8°C | Ffo | 25.40 | 6.36 | 1.46 | 2.35 |
| 8°C | Ffo | 23.89 | 6.86 | 1.50 | 2.36 |
| 8°C | Ffo | 24.04 | 7.46 | 1.58 | 2.34 |
| 8°C | Ffo | 25.11 | 6.55 | 1.83 | 2.69 |
| 8°C | Ffo | 25.91 | 7.20 | 1.62 | 2.42 |
| 8°C | Ffo | 25.29 | 7.15 | 1.55 | 2.38 |
| 8°C | Ffo | 24.68 | 6.71 | 1.69 | 2.45 |
| 8°C | Ffo | 24.09 | 6.50 | 1.51 | 2.17 |
| 8°C | Ffo | 24.58 | 6.33 | 1.55 | 2.46 |
| 8°C | Ffo | 24.13 | 7.03 | 1.50 | 2.44 |
| 8°C | Ffo | 25.27 | 6.83 | 1.71 | 2.38 |
| 8°C | Ffo | 24.33 | 6.34 | 1.75 | 2.52 |
| 8°C | Ffo | 24.35 | 6.56 | 1.53 | 2.40 |
| 8°C | Ffo | 24.55 | 6.57 | 1.98 | 2.42 |
| 8°C | Ffo | 25.39 | 7.24 | 1.90 | 2.61 |
| 8°C | Ffo | 24.16 | 6.90 | 1.73 | 2.48 |
| 8°C | Ffo | 25.45 | 6.49 | 1.48 | 2.39 |
| 8°C | Ffo | 24.25 | 6.43 | 1.83 | 2.31 |
| 8°C | Ffo | 24.71 | 7.42 | 1.43 | 2.33 |
| 8°C | Ffo | 25.01 | 6.65 | 1.85 | 2.44 |
| 8°C | Ffo | 25.38 | 6.78 | 1.70 | 2.46 |
| 8°C | Ffo | 25.06 | 6.41 | 1.78 | 2.53 |
| 8°C | Ffo | 25.19 | 7.45 | 1.38 | 2.29 |
| 8°C | Ffo | 25.50 | 6.61 | 1.59 | 2.40 |
| 8°C | Ffo | 25.74 | 6.52 | 1.67 | 2.42 |
| 8°C | Ffo | 24.62 | 6.60 | 1.79 | 2.60 |
| 8°C | Ffo | 24.29 | 6.38 | 1.50 | 2.47 |
| 8°C | Ffo | 23.78 | 6.50 | 1.89 | 2.25 |
| 8°C | Ffo | 24.17 | 7.04 | 1.39 | 2.43 |
| 8°C | Ffo | 23.39 | 6.46 | 1.60 | 2.28 |
| 8°C | Ffo | 24.95 | 7.06 | 1.85 | 2.63 |
| 8°C | Ffo | 24.84 | 6.55 | 1.53 | 2.46 |
| 8°C | Ffo | 23.75 | 6.88 | 1.49 | 2.20 |
| 8°C | Ffo | 24.20 | 7.00 | 1.83 | 2.23 |
| 8°C | Ffo | 25.81 | 6.45 | 1.67 | 2.27 |
| 8°C | Ffo | 26.14 | 6.79 | 1.47 | 2.39 |
| 8°C | Ffo | 26.21 | 6.58 | 1.76 | 2.64 |
| 8°C | Ffo | 24.75 | 6.84 | 1.53 | 2.42 |
| 8°C | Ffo | 25.07 | 7.24 | 1.77 | 2.29 |
| 8°C | Ffo | 24.95 | 6.73 | 1.49 | 2.36 |
| 8°C | Ffo | 24.09 | 6.58 | 1.61 | 2.48 |
| 8°C | Ffo | 23.92 | 6.90 | 1.64 | 2.36 |
| 8°C | Ffo | 25.36 | 6.27 | 1.53 | 2.47 |
| 8°C | Ffo | 24.62 | 7.56 | 1.36 | 2.48 |
| 8°C | Ffo | 25.16 | 6.38 | 1.55 | 2.56 |
| 8°C | Ffo | 25.41 | 6.67 | 1.72 | 2.43 |
| 8°C | Ffo | 24.25 | 6.18 | 1.53 | 2.36 |
| 8°C | Ffo | 25.40 | 7.06 | 1.89 | 2.40 |
| 8°C | Ffo | 26.02 | 6.52 | 1.55 | 2.38 |
| 8°C | Ffo | 25.72 | 7.34 | 1.61 | 2.40 |
| 8°C | Ffo | 26.22 | 6.54 | 1.60 | 2.61 |
| 8°C | Ffo | 25.51 | 7.07 | 1.36 | 2.52 |
| 8°C | Ffo | 25.40 | 6.60 | 1.48 | 2.48 |
| 8°C | Ffo | 25.72 | 6.61 | 1.63 | 2.41 |
| 8°C | Ffo | 25.56 | 6.86 | 1.76 | 2.54 |
| 10°C | H | 15.02 | 7.26 | 1.02 | 1.10 |
| 10°C | H | 16.58 | 6.60 | 0.87 | 1.04 |
| 10°C | H | 17.96 | 7.43 | 1.05 | 1.43 |
| 10°C | H | 17.88 | 8.14 | 1.29 | 1.32 |
| 10°C | H | 16.68 | 6.60 | 0.97 | 1.31 |
| 10°C | H | 16.35 | 7.71 | 0.87 | 1.19 |
| 10°C | H | 17.41 | 7.55 | 1.14 | 1.44 |
| 10°C | H | 16.79 | 7.23 | 1.11 | 1.36 |
| 10°C | H | 15.98 | 7.22 | 0.99 | 1.63 |
| 10°C | H | 17.47 | 6.54 | 0.92 | 1.43 |
| 10°C | H | 17.73 | 7.36 | 1.14 | 1.32 |
| 10°C | H | 18.29 | 6.78 | 1.21 | 1.66 |
| 10°C | H | 16.96 | 7.13 | 1.22 | 1.53 |
| 10°C | H | 17.39 | 6.93 | 0.94 | 1.23 |
| 10°C | H | 17.89 | 7.00 | 1.19 | 1.80 |
| 10°C | H | 16.88 | 6.83 | 1.20 | 1.49 |
| 10°C | H | 16.78 | 7.05 | 1.04 | 1.70 |
| 10°C | H | 16.82 | 6.70 | 1.09 | 1.55 |
| 10°C | H | 17.42 | 6.67 | 1.18 | 1.47 |
| 10°C | H | 18.01 | 7.68 | 1.25 | 1.18 |
| 10°C | H | 17.32 | 7.98 | 1.22 | 1.40 |
| 10°C | H | 17.64 | 6.71 | 1.18 | 1.45 |
| 10°C | H | 17.61 | 6.67 | 1.21 | 1.55 |
| 10°C | H | 16.92 | 7.25 | 1.28 | 1.49 |
| 10°C | H | 17.19 | 7.42 | 1.19 | 1.55 |
| 10°C | H | 17.10 | 7.12 | 1.10 | 1.61 |
| 10°C | H | 13.12 | 6.79 | 1.19 | 1.37 |
| 10°C | H | 17.20 | 6.74 | 1.23 | 1.26 |
| 10°C | H | 17.34 | 6.80 | 1.15 | 1.64 |
| 10°C | H | 18.40 | 7.14 | 1.23 | 1.35 |
| 10°C | H | 17.12 | 7.38 | 1.23 | 1.57 |
| 10°C | H | 17.97 | 6.98 | 1.27 | 1.46 |
| 10°C | H | 15.45 | 7.06 | 1.22 | 1.45 |
| 10°C | H | 16.97 | 8.15 | 1.11 | 1.34 |
| 10°C | H | 17.98 | 7.60 | 1.14 | 1.56 |
| 10°C | H | 15.56 | 6.82 | 1.15 | 1.64 |
| 10°C | H | 15.37 | 6.67 | 0.94 | 1.46 |
| 10°C | H | 16.46 | 6.82 | 1.05 | 1.50 |
| 10°C | H | 16.44 | 6.46 | 0.98 | 1.39 |
| 10°C | H | 18.10 | 7.37 | 0.98 | 1.14 |
| 10°C | H | 17.43 | 7.32 | 0.97 | 1.43 |
| 10°C | H | 17.49 | 6.96 | 1.26 | 1.33 |
| 10°C | H | 16.61 | 7.21 | 1.23 | 1.43 |
| 10°C | H | 17.42 | 6.45 | 0.98 | 1.60 |
| 10°C | H | 16.40 | 7.70 | 1.19 | 1.48 |
| 10°C | H | 16.35 | 7.63 | 1.17 | 1.49 |
| 10°C | H | 17.84 | 8.39 | 0.87 | 1.35 |
| 10°C | H | 15.88 | 7.52 | 1.17 | 1.39 |
| 10°C | H | 15.82 | 7.38 | 1.05 | 1.53 |
| 10°C | H | 16.84 | 8.00 | 1.05 | 1.47 |
| 10°C | H | 17.49 | 7.05 | 1.08 | 1.31 |
| 10°C | H | 17.74 | 7.79 | 1.35 | 1.54 |
| 10°C | H | 17.07 | 7.58 | 1.32 | 1.32 |
| 10°C | H | 17.12 | 7.97 | 1.17 | 1.57 |
| 10°C | H | 17.10 | 7.46 | 1.23 | 1.48 |
| 10°C | H | 16.22 | 7.71 | 1.34 | 1.63 |
| 10°C | H | 17.41 | 8.03 | 1.16 | 1.49 |
| 10°C | H | 16.08 | 7.57 | 1.16 | 1.55 |
| 10°C | H | 17.56 | 7.14 | 1.07 | 1.52 |
| 10°C | H | 18.01 | 8.12 | 1.45 | 1.26 |
| 10°C | H | 17.28 | 7.95 | 1.26 | 1.46 |
| 10°C | H | 17.48 | 6.47 | 1.14 | 1.60 |
| 10°C | H | 17.16 | 7.86 | 1.08 | 1.64 |
| 10°C | H | 17.00 | 7.98 | 1.18 | 1.60 |
| 10°C | H | 17.43 | 7.37 | 1.09 | 1.40 |
| 10°C | H | 17.19 | 8.15 | 1.11 | 1.41 |
| 10°C | H | 16.89 | 7.66 | 1.15 | 1.41 |
| 10°C | H | 15.17 | 8.05 | 1.24 | 1.67 |
| 10°C | H | 16.73 | 8.15 | 1.23 | 1.48 |
| 10°C | H | 17.92 | 8.23 | 1.16 | 1.30 |
| 10°C | H | 17.87 | 7.65 | 1.23 | 1.59 |
| 10°C | H | 17.10 | 8.11 | 1.55 | 1.39 |
| 10°C | H | 17.50 | 7.91 | 1.24 | 1.54 |
| 10°C | H | 17.56 | 7.79 | 1.30 | 1.60 |
| 10°C | H | 17.56 | 7.97 | 1.16 | 1.53 |
| 10°C | H | 17.02 | 7.97 | 1.16 | 1.47 |
| 10°C | H | 17.29 | 7.51 | 1.18 | 1.31 |
| 10°C | H | 16.51 | 8.38 | 1.38 | 1.54 |
| 10°C | H | 17.99 | 8.72 | 1.14 | 1.32 |
| 10°C | H | 17.19 | 8.39 | 1.10 | 1.57 |
| 10°C | H | 18.17 | 7.64 | 0.80 | 1.48 |
| 10°C | H | 17.57 | 8.04 | 1.17 | 1.63 |
| 10°C | H | 22.98 | 8.21 | 1.34 | 1.49 |
| 10°C | EM | 24.65 | 6.87 | 1.28 | 1.94 |
| 10°C | EM | 24.41 | 8.22 | 1.57 | 1.98 |
| 10°C | EM | 22.91 | 7.85 | 1.50 | 2.10 |
| 10°C | EM | 23.37 | 7.51 | 1.45 | 2.00 |
| 10°C | EM | 22.81 | 8.29 | 1.37 | 2.01 |
| 10°C | EM | 24.36 | 7.74 | 1.45 | 2.00 |
| 10°C | EM | 24.95 | 7.06 | 1.53 | 2.28 |
| 10°C | EM | 22.39 | 6.88 | 1.53 | 2.17 |
| 10°C | EM | 22.92 | 6.99 | 1.36 | 2.04 |
| 10°C | EM | 23.20 | 7.72 | 1.54 | 1.91 |
| 10°C | EM | 22.46 | 6.27 | 1.50 | 1.95 |
| 10°C | EM | 23.65 | 7.26 | 1.52 | 1.83 |
| 10°C | EM | 24.75 | 7.00 | 1.37 | 2.22 |
| 10°C | EM | 23.65 | 7.41 | 1.39 | 2.22 |
| 10°C | EM | 22.78 | 6.72 | 1.47 | 2.17 |
| 10°C | EM | 23.61 | 8.42 | 1.36 | 2.10 |
| 10°C | EM | 23.24 | 7.77 | 1.45 | 2.16 |
| 10°C | EM | 23.44 | 7.57 | 1.32 | 2.08 |
| 10°C | EM | 24.33 | 7.20 | 1.42 | 2.04 |
| 10°C | EM | 24.05 | 7.35 | 1.22 | 2.14 |
| 10°C | EM | 24.25 | 7.29 | 1.41 | 2.03 |
| 10°C | EM | 24.01 | 6.47 | 1.53 | 2.03 |
| 10°C | EM | 24.51 | 6.67 | 1.41 | 2.08 |
| 10°C | EM | 23.76 | 7.16 | 1.36 | 1.98 |
| 10°C | EM | 21.95 | 7.56 | 1.52 | 2.09 |
| 10°C | EM | 23.82 | 8.01 | 1.23 | 2.01 |
| 10°C | EM | 23.37 | 7.40 | 1.39 | 1.97 |
| 10°C | EM | 23.31 | 7.25 | 1.25 | 2.03 |
| 10°C | EM | 24.87 | 6.86 | 1.45 | 1.85 |
| 10°C | EM | 24.17 | 7.20 | 1.40 | 1.81 |
| 10°C | EM | 23.04 | 7.33 | 1.60 | 1.80 |
| 10°C | EM | 24.61 | 6.98 | 1.43 | 2.09 |
| 10°C | EM | 23.95 | 6.98 | 1.29 | 2.05 |
| 10°C | EM | 24.13 | 7.64 | 1.44 | 1.93 |
| 10°C | EM | 24.04 | 7.42 | 1.44 | 1.88 |
| 10°C | EM | 22.99 | 7.04 | 1.43 | 2.00 |
| 10°C | EM | 24.25 | 7.57 | 1.56 | 1.98 |
| 10°C | EM | 22.82 | 6.20 | 1.41 | 1.79 |
| 10°C | EM | 24.70 | 7.80 | 1.46 | 1.80 |
| 10°C | EM | 24.28 | 6.79 | 1.58 | 2.32 |
| 10°C | EM | 23.69 | 7.17 | 1.45 | 2.10 |
| 10°C | EM | 23.91 | 7.55 | 1.38 | 2.08 |
| 10°C | EM | 24.04 | 6.75 | 1.33 | 2.22 |
| 10°C | EM | 24.30 | 6.69 | 1.78 | 2.22 |
| 10°C | EM | 23.63 | 6.87 | 1.66 | 2.05 |
| 10°C | EM | 22.62 | 6.44 | 1.53 | 1.99 |
| 10°C | EM | 23.17 | 6.77 | 1.66 | 2.12 |
| 10°C | EM | 26.74 | 7.71 | 1.78 | 2.11 |
| 10°C | EM | 23.55 | 7.33 | 1.34 | 2.00 |
| 10°C | EM | 24.61 | 7.84 | 1.61 | 2.04 |
| 10°C | EM | 23.04 | 7.36 | 1.38 | 2.30 |
| 10°C | EM | 24.76 | 7.17 | 1.70 | 2.13 |
| 10°C | EM | 23.73 | 7.08 | 1.37 | 2.15 |
| 10°C | EM | 24.64 | 7.00 | 1.37 | 2.22 |
| 10°C | EM | 24.19 | 6.36 | 1.31 | 2.24 |
| 10°C | EM | 24.34 | 7.02 | 1.42 | 2.25 |
| 10°C | EM | 24.06 | 7.86 | 1.40 | 2.32 |
| 10°C | EM | 24.00 | 6.26 | 1.44 | 2.33 |
| 10°C | EM | 24.28 | 6.93 | 1.45 | 2.33 |
| 10°C | EM | 24.58 | 7.34 | 1.48 | 2.40 |
| 10°C | EM | 24.83 | 7.04 | 1.77 | 2.14 |
| 10°C | EM | 24.97 | 7.76 | 1.41 | 2.07 |
| 10°C | EM | 24.19 | 7.73 | 1.58 | 2.16 |
| 10°C | EM | 23.75 | 7.50 | 1.68 | 2.18 |
| 10°C | EM | 23.43 | 7.56 | 1.69 | 2.07 |
| 10°C | EM | 24.61 | 7.94 | 1.40 | 2.17 |
| 10°C | EM | 22.75 | 7.05 | 1.51 | 2.30 |
| 10°C | EM | 27.17 | 7.70 | 1.40 | 2.40 |
| 10°C | EM | 24.76 | 6.62 | 1.26 | 2.41 |
| 10°C | EM | 23.79 | 7.70 | 1.73 | 2.50 |
| 10°C | EM | 24.03 | 6.38 | 1.55 | 2.37 |
| 10°C | EM | 23.36 | 7.67 | 1.52 | 2.50 |
| 10°C | EM | 24.92 | 7.52 | 1.71 | 2.25 |
| 10°C | EM | 24.18 | 8.06 | 1.88 | 2.48 |
| 10°C | EM | 24.87 | 6.76 | 1.78 | 1.93 |
| 10°C | EM | 23.49 | 7.15 | 1.75 | 2.29 |
| 10°C | EM | 23.77 | 7.59 | 1.72 | 2.25 |
| 10°C | EM | 24.13 | 7.50 | 1.47 | 2.17 |
| 10°C | EM | 26.53 | 7.32 | 1.52 | 2.10 |
| 10°C | EM | 25.48 | 6.83 | 1.74 | 2.31 |
| 10°C | EM | 26.31 | 7.14 | 1.67 | 2.51 |
| 10°C | EM | 22.80 | 7.37 | 1.46 | 2.33 |
| 10°C | EM | 27.28 | 7.06 | 1.32 | 2.11 |
| 10°C | EM | 24.42 | 6.53 | 1.29 | 1.96 |
| 10°C | EM | 23.95 | 7.33 | 1.41 | 2.54 |
| 10°C | EM | 24.59 | 7.03 | 1.59 | 2.18 |
| 10°C | EM | 23.31 | 6.54 | 1.65 | 2.17 |
| 10°C | EM | 24.60 | 7.32 | 1.74 | 2.26 |
| 10°C | EM | 25.03 | 6.98 | 1.59 | 2.15 |
| 10°C | EM | 24.80 | 6.50 | 1.48 | 2.26 |
| 10°C | EM | 24.00 | 6.80 | 1.41 | 2.44 |
| 10°C | EM | 25.26 | 7.42 | 1.39 | 2.37 |
| 10°C | EM | 24.43 | 7.14 | 1.56 | 2.24 |
| 10°C | EM | 25.15 | 7.81 | 1.57 | 2.36 |
| 10°C | EM | 24.47 | 7.19 | 1.28 | 2.23 |
| 10°C | EM | 23.86 | 7.07 | 1.58 | 2.24 |
| 10°C | EM | 25.39 | 8.14 | 1.82 | 2.15 |
| 10°C | EM | 24.91 | 7.16 | 1.74 | 2.22 |
| 10°C | EM | 23.73 | 6.54 | 1.55 | 2.32 |
| 10°C | Ffo | 24.97 | 6.33 | 1.69 | 2.33 |
| 10°C | Ffo | 22.48 | 6.95 | 1.37 | 2.43 |
| 10°C | Ffo | 23.93 | 6.22 | 1.50 | 1.91 |
| 10°C | Ffo | 23.68 | 6.28 | 1.46 | 2.40 |
| 10°C | Ffo | 24.49 | 6.56 | 1.41 | 2.26 |
| 10°C | Ffo | 26.10 | 6.12 | 1.55 | 2.37 |
| 10°C | Ffo | 24.57 | 6.72 | 1.64 | 2.57 |
| 10°C | Ffo | 24.95 | 6.51 | 1.42 | 2.56 |
| 10°C | Ffo | 22.90 | 6.65 | 1.44 | 2.41 |
| 10°C | Ffo | 23.99 | 6.34 | 1.65 | 2.28 |
| 10°C | Ffo | 25.44 | 6.18 | 1.50 | 2.46 |
| 10°C | Ffo | 25.21 | 6.45 | 1.72 | 2.79 |
| 10°C | Ffo | 24.91 | 6.42 | 1.73 | 2.46 |
| 10°C | Ffo | 23.83 | 6.88 | 1.82 | 2.33 |
| 10°C | Ffo | 23.80 | 6.69 | 1.50 | 2.23 |
| 10°C | Ffo | 23.83 | 6.27 | 1.50 | 2.44 |
| 10°C | Ffo | 24.01 | 6.80 | 1.46 | 2.04 |
| 10°C | Ffo | 23.69 | 6.56 | 1.74 | 2.37 |
| 10°C | Ffo | 22.82 | 6.77 | 1.70 | 2.29 |
| 10°C | Ffo | 24.75 | 7.65 | 1.80 | 1.96 |
| 10°C | Ffo | 24.76 | 6.75 | 1.57 | 2.18 |
| 10°C | Ffo | 24.32 | 7.53 | 1.75 | 1.90 |
| 10°C | Ffo | 24.19 | 6.71 | 1.54 | 2.25 |
| 10°C | Ffo | 23.67 | 6.62 | 1.55 | 2.47 |
| 10°C | Ffo | 22.66 | 6.59 | 1.80 | 2.20 |
| 10°C | Ffo | 24.84 | 6.42 | 1.36 | 2.38 |
| 10°C | Ffo | 23.41 | 6.31 | 1.40 | 2.04 |
| 10°C | Ffo | 23.35 | 6.05 | 1.40 | 2.11 |
| 10°C | Ffo | 21.39 | 5.54 | 1.58 | 2.03 |
| 10°C | Ffo | 22.85 | 6.21 | 1.55 | 2.38 |
| 10°C | Ffo | 22.99 | 6.56 | 1.45 | 1.98 |
| 10°C | Ffo | 23.09 | 6.81 | 1.29 | 1.49 |
| 10°C | Ffo | 23.79 | 6.46 | 1.55 | 2.40 |
| 10°C | Ffo | 23.81 | 6.72 | 1.54 | 2.46 |
| 10°C | Ffo | 23.06 | 6.30 | 1.57 | 2.25 |
| 10°C | Ffo | 23.27 | 6.47 | 1.88 | 2.23 |
| 10°C | Ffo | 23.21 | 6.89 | 1.65 | 2.36 |
| 10°C | Ffo | 23.08 | 6.68 | 1.46 | 1.87 |
| 10°C | Ffo | 23.09 | 6.00 | 1.67 | 2.16 |
| 10°C | Ffo | 22.95 | 6.86 | 1.37 | 1.84 |
| 10°C | Ffo | 22.38 | 6.85 | 1.24 | 2.27 |
| 10°C | Ffo | 23.01 | 7.17 | 1.51 | 1.78 |
| 10°C | Ffo | 23.00 | 6.60 | 1.32 | 2.25 |
| 10°C | Ffo | 25.50 | 5.90 | 1.07 | 2.00 |
| 10°C | Ffo | 26.04 | 6.63 | 1.49 | 1.77 |
| 10°C | Ffo | 24.61 | 6.85 | 1.64 | 2.22 |
| 10°C | Ffo | 23.24 | 6.71 | 1.40 | 2.40 |
| 10°C | Ffo | 24.88 | 6.69 | 1.40 | 1.97 |
| 10°C | Ffo | 23.40 | 6.50 | 1.32 | 2.50 |
| 10°C | Ffo | 23.49 | 6.37 | 1.24 | 1.93 |
| 10°C | Ffo | 20.40 | 6.59 | 1.17 | 2.46 |
| 10°C | Ffo | 24.71 | 7.79 | 1.53 | 1.93 |
| 10°C | Ffo | 25.27 | 6.35 | 1.46 | 2.23 |
| 10°C | Ffo | 25.02 | 6.52 | 1.20 | 2.51 |
| 10°C | Ffo | 24.03 | 6.37 | 1.52 | 2.16 |
| 10°C | Ffo | 22.05 | 5.69 | 1.05 | 2.42 |
| 10°C | Ffo | 24.05 | 6.26 | 1.63 | 1.92 |
| 10°C | Ffo | 24.08 | 6.48 | 1.30 | 2.21 |
| 10°C | Ffo | 22.93 | 6.90 | 1.41 | 2.02 |
| 10°C | Ffo | 22.87 | 5.98 | 1.54 | 2.10 |
| 10°C | Ffo | 23.33 | 6.53 | 1.43 | 2.12 |
| 10°C | Ffo | 26.44 | 6.21 | 1.29 | 2.34 |
| 10°C | Ffo | 24.68 | 5.96 | 1.46 | 2.09 |
| 10°C | Ffo | 24.17 | 6.37 | 1.72 | 2.32 |
| 10°C | Ffo | 23.74 | 6.03 | 1.53 | 2.14 |
| 10°C | Ffo | 23.19 | 6.11 | 1.19 | 2.32 |
| 10°C | Ffo | 24.28 | 6.62 | 1.34 | 2.09 |
| 10°C | Ffo | 23.12 | 6.15 | 1.81 | 2.27 |
| 10°C | Ffo | 23.22 | 6.65 | 1.36 | 2.14 |
| 10°C | Ffo | 23.60 | 6.52 | 1.25 | 2.25 |
| 10°C | Ffo | 24.66 | 5.76 | 1.66 | 1.94 |
| 10°C | Ffo | 23.09 | 6.76 | 1.45 | 2.12 |
| 10°C | Ffo | 23.11 | 6.78 | 1.62 | 2.05 |
| 10°C | Ffo | 25.23 | 6.49 | 1.29 | 1.80 |
| 10°C | Ffo | 22.68 | 7.08 | 1.21 | 2.09 |
| 10°C | Ffo | 22.62 | 6.46 | 1.54 | 1.81 |
| 10°C | Ffo | 23.47 | 6.94 | 1.23 | 2.08 |
| 10°C | Ffo | 24.36 | 7.47 | 1.73 | 2.59 |
| 10°C | Ffo | 24.40 | 6.65 | 1.43 | 2.41 |
| 10°C | Ffo | 24.61 | 6.14 | 1.71 | 2.08 |
| 10°C | Ffo | 26.56 | 6.48 | 1.54 | 2.35 |
| 10°C | Ffo | 23.98 | 6.69 | 1.77 | 2.37 |
| 10°C | Ffo | 24.34 | 6.82 | 1.50 | 2.09 |
| 10°C | Ffo | 23.79 | 7.46 | 1.54 | 2.23 |
| 10°C | Ffo | 24.80 | 6.09 | 1.63 | 2.34 |
| 10°C | Ffo | 22.76 | 6.57 | 1.47 | 2.38 |
| 10°C | Ffo | 23.31 | 6.10 | 1.32 | 2.06 |
| 10°C | Ffo | 23.01 | 6.61 | 1.42 | 2.07 |
| 10°C | Ffo | 24.18 | 6.08 | 1.41 | 2.04 |
| 10°C | Ffo | 23.26 | 7.04 | 1.44 | 2.17 |
| 10°C | Ffo | 23.66 | 6.66 | 1.37 | 2.24 |
| 10°C | Ffo | 24.57 | 6.55 | 1.54 | 2.28 |
| 10°C | Ffo | 21.37 | 6.48 | 1.54 | 2.43 |
| 10°C | Ffo | 23.86 | 8.15 | 1.52 | 2.05 |
| 10°C | Ffo | 24.04 | 7.08 | 1.50 | 2.63 |
| 10°C | Ffo | 23.57 | 6.94 | 1.49 | 2.37 |
| 10°C | Ffo | 24.13 | 6.72 | 1.52 | 2.07 |
| 10°C | Ffo | 21.25 | 6.59 | 1.48 | 2.35 |
| 10°C | Ffo | 26.29 | 6.53 | 1.58 | 2.07 |
| 10°C | Ffo | 24.45 | 6.23 | 1.48 | 2.50 |
| 10°C | Ffo | 24.89 | 6.25 | 1.46 | 2.37 |
| 10°C | Ffo | 24.87 | 6.76 | 1.52 | 2.41 |
| 10°C | Ffo | 23.73 | 6.25 | 1.32 | 2.32 |
| 10°C | Ffo | 22.71 | 6.25 | 1.50 | 2.35 |
| 10°C | Ffo | 26.69 | 7.16 | 1.34 | 2.04 |
| 10°C | Ffo | 23.90 | 6.03 | 1.45 | 2.28 |
| 10°C | Ffo | 24.62 | 6.77 | 1.46 | 1.92 |
| 10°C | Ffo | 18.85 | 6.66 | 1.27 | 2.38 |
| 10°C | Ffo | 22.85 | 6.66 | 1.37 | 1.45 |
| 10°C | Ffo | 25.29 | 6.42 | 1.61 | 2.21 |
| 10°C | Ffo | 22.80 | 6.79 | 1.43 | 2.40 |
| 10°C | Ffo | 23.35 | 6.31 | 1.36 | 2.12 |
| 10°C | Ffo | 24.18 | 6.24 | 1.78 | 2.09 |
| 10°C | Ffo | 22.15 | 6.62 | 1.48 | 2.15 |
| 10°C | Ffo | 23.54 | 6.16 | 1.37 | 2.20 |
| 10°C | Ffo | 23.26 | 6.02 | 1.44 | 2.10 |
| 10°C | Ffo | 23.74 | 6.26 | 1.51 | 2.18 |
| 10°C | Ffo | 23.83 | 6.53 | 1.42 | 2.34 |
| 10°C | Ffo | 23.75 | 6.65 | 1.51 | 2.16 |
| 12°C | H | 13.32 | 6.97 | 1.15 | 0.94 |
| 12°C | H | 15.95 | 6.78 | 1.00 | 1.02 |
| 12°C | H | 15.83 | 6.99 | 0.90 | 1.21 |
| 12°C | H | 14.84 | 25.11 | 0.90 | 0.99 |
| 12°C | H | 17.85 | 7.89 | 0.98 | 1.54 |
| 12°C | H | 14.94 | 7.88 | 0.92 | 1.84 |
| 12°C | H | 16.58 | 6.93 | 0.84 | 1.27 |
| 12°C | H | 15.72 | 7.41 | 0.97 | 0.93 |
| 12°C | H | 15.47 | 7.20 | 0.92 | 1.34 |
| 12°C | H | 13.00 | 6.74 | 0.99 | 1.17 |
| 12°C | H | 17.11 | 6.49 | 1.06 | 0.97 |
| 12°C | H | 15.29 | 6.56 | 0.90 | 1.28 |
| 12°C | H | 15.29 | 6.56 | 0.90 | 1.28 |
| 12°C | H | 14.94 | 7.88 | 0.92 | 1.84 |
| 12°C | H | 15.29 | 6.59 | 0.94 | 1.18 |
| 12°C | H | 15.18 | 6.92 | 0.90 | 1.46 |
| 12°C | EM | 21.88 | 8.20 | 1.24 | 2.84 |
| 12°C | EM | 21.88 | 6.96 | 1.14 | 2.28 |
| 12°C | EM | 25.29 | 7.26 | 1.50 | 2.21 |
| 12°C | EM | 23.08 | 6.93 | 1.12 | 2.09 |
| 12°C | EM | 24.63 | 6.79 | 1.70 | 2.17 |
| 12°C | EM | 23.15 | 6.78 | 1.26 | 2.29 |
| 12°C | EM | 22.14 | 7.50 | 1.31 | 2.27 |
| 12°C | EM | 22.45 | 6.08 | 1.27 | 1.95 |
| 12°C | EM | 24.55 | 7.34 | 1.60 | 2.46 |
| 12°C | EM | 23.14 | 6.86 | 1.38 | 2.10 |
| 12°C | EM | 24.07 | 6.76 | 1.22 | 1.95 |
| 12°C | EM | 22.64 | 7.53 | 1.28 | 2.20 |
| 12°C | EM | 23.93 | 6.87 | 1.60 | 2.13 |
| 12°C | Ffo | 27.56 | 7.62 | 1.68 | 2.73 |
| 12°C | Ffo | 27.84 | 8.64 | 1.53 | 2.96 |
| 12°C | Ffo | 28.67 | 6.56 | 1.94 | 2.75 |
| 12°C | Ffo | 27.55 | 7.94 | 1.91 | 2.90 |
| 12°C | Ffo | 28.21 | 6.80 | 2.09 | 2.77 |
| 12°C | Ffo | 26.98 | 6.92 | 1.62 | 3.09 |
| 12°C | Ffo | 27.44 | 6.77 | 1.75 | 2.79 |
| 12°C | Ffo | 28.25 | 7.38 | 1.83 | 2.69 |
| 12°C | Ffo | 27.33 | 6.88 | 1.27 | 2.58 |
| 12°C | Ffo | 28.58 | 7.61 | 1.82 | 2.70 |
| 12°C | Ffo | 28.05 | 7.86 | 1.64 | 2.69 |
| 12°C | Ffo | 27.66 | 5.00 | 1.77 | 2.40 |
| 12°C | Ffo | 28.93 | 7.62 | 1.68 | 2.62 |
| 12°C | Ffo | 26.78 | 7.16 | 1.85 | 2.55 |
| 12°C | Ffo | 26.87 | 6.89 | 1.86 | 2.77 |
| 12°C | Ffo | 28.40 | 7.05 | 1.77 | 3.05 |
| 12°C | Ffo | 29.95 | 8.47 | 2.25 | 3.28 |
| 12°C | Ffo | 28.55 | 7.21 | 1.68 | 2.85 |
| 12°C | Ffo | 28.22 | 7.51 | 1.71 | 3.00 |
| 12°C | Ffo | 26.82 | 8.09 | 1.81 | 2.95 |
