# Supplementary material for: Strong Effects of Temperature on the Early Life Stages of a Cold Stenothermal Fish Species, Brown Trout (Salmo trutta L.)
Source: PLoS One. 2016 May 12;11(5):e0155487. doi: 10.1371/journal.pone.0155487 (PMC4865038; doi:10.1371/journal.pone.0155487)
Supplement: Supplementary file 3 [file pone.0155487.s003.docx]

Fertilisation												
		C	H	N	S	O	Weight mg	C/N	% P	%Ash	V cal J.	VE J.ind-1
4°C	1	51.56	7.80	10.98	0.75	23.33	2.00	4.69	63.48	5.58	26.10	52.20
	2	51.91	7.86	10.82	0.79	22.58	2.01	4.80	62.56	6.04	26.45	53.19
	3	52.62	7.94	10.63	0.79	23.33	2.03	4.95	61.42	4.69	26.69	54.26
	5	51.05	7.68	11.03	0.84	23.45	2.03	4.63	63.73	5.96	25.72	52.25
	6	54.34	8.13	11.43	0.92	23.46	2.01	4.75	66.08	1.71	27.59	55.57
	7	53.62	8.24	11.58	0.88	23.72	2.00	4.63	66.94	1.96	27.43	54.87
	8	54.94	8.31	11.00	0.86	22.78	2.01	4.99	63.58	2.12	28.17	56.50
6°C	1	53.57	8.37	11.61	0.77	22.78	2.00	4.61	67.11	2.89	27.75	55.52
	2	54.75	8.36	11.03	0.81	22.78	2.00	4.96	63.74	2.27	28.18	56.48
	3	54.92	8.30	10.83	0.82	22.78	2.01	5.07	62.60	2.35	28.16	56.53
	4	53.26	8.35	11.37	0.75	22.78	2.00	4.68	65.74	3.49	27.61	55.24
	5	52.89	7.86	11.15	0.86	22.78	2.00	4.75	64.42	4.47	26.78	53.65
	6	53.20	8.10	11.44	0.76	22.78	2.00	4.65	66.12	3.72	27.23	54.48
	7	53.42	8.11	11.24	0.82	22.78	2.00	4.75	64.98	3.62	27.33	54.68
	8	55.40	8.46	10.79	0.79	22.78	2.00	5.13	62.35	1.78	28.56	57.21
8°C	1	53.75	8.33	11.22	0.80	22.78	1.99	4.79	64.82	3.12	27.76	55.17
	2	55.14	8.48	10.74	0.82	22.78	1.99	5.13	62.07	2.03	28.50	56.60
	3	52.84	7.96	11.30	1.03	22.78	1.99	4.68	65.30	4.10	26.92	53.69
	4	51.61	7.92	11.58	0.89	22.78	1.99	4.46	66.92	5.22	26.40	52.63
	5	51.50	7.95	11.21	0.84	22.78	1.98	4.59	64.79	5.72	26.38	52.31
	6	52.80	7.78	11.32	0.89	22.78	1.99	4.67	65.41	4.43	26.63	53.04
	7	54.03	7.84	11.05	0.88	22.78	1.98	4.89	63.86	3.41	27.17	53.81
	8	54.95	8.20	10.74	0.77	22.78	1.99	5.12	62.05	2.56	28.02	55.70
10°C	1	53.80	7.93	11.15	0.85	22.78	1.99	4.83	64.43	3.49	27.22	54.08
	2	53.18	7.95	11.10	0.84	22.78	2.00	4.79	64.13	4.16	27.00	54.01
	3	54.40	8.19	10.70	0.76	22.78	1.98	5.08	61.85	3.17	27.80	55.17
	4	53.60	7.58	10.96	0.98	22.78	2.00	4.89	63.35	4.09	26.65	53.31
	5	52.75	7.96	11.24	0.79	22.78	2.00	4.70	64.94	4.48	26.86	53.73
	6	52.61	7.91	11.50	0.85	22.78	1.98	4.58	66.44	4.36	26.74	52.99
	7	53.69	8.20	10.99	0.74	22.78	2.00	4.89	63.52	3.60	27.55	55.10
	8	52.92	8.13	11.61	0.80	22.78	2.01	4.56	67.10	3.77	27.17	54.61
12°C	1	55.46	8.06	10.35	0.75	22.78	2.00	5.36	59.80	2.60	28.00	56.02
	2	53.33	8.05	11.37	0.80	22.78	2.01	4.69	65.74	3.67	27.20	54.68
	3	53.88	7.71	11.05	0.89	22.78	1.97	4.87	63.89	3.68	26.93	53.18
	4	53.22	7.62	11.39	0.92	22.78	2.01	4.67	65.81	4.08	26.55	53.49
	5	54.78	7.86	10.53	0.79	22.78	1.97	5.20	60.87	3.25	27.47	54.18
	6	52.47	7.97	11.77	0.84	22.78	2.10	4.46	68.00	4.17	26.77	56.33
	7	51.68	7.81	11.81	0.86	22.78	1.96	4.38	68.27	5.06	26.26	51.47
	8	54.07	7.99	11.09	0.80	22.78	1.96	4.88	64.08	3.27	27.40	53.64

Eyed stage												
		N	C	H	S	O	Weight mg	C/N	% P	%Ash	V cal J.	V E J.ind-1
4°C	1	11.06	53.83	9.00	0.67	24.70	1.98	4.87	63.94	0.73	28.41	56.11
	2	11.84	52.77	8.90	0.67	22.74	2.01	4.46	68.43	3.08	28.22	56.74
	3	10.58	55.14	8.96	0.68	24.51	1.03	5.21	61.15	0.13	28.86	29.82
	4	11.63	53.54	8.85	0.65	24.31	2.12	4.61	67.19	1.03	28.14	59.75
	5	11.38	54.20	8.82	0.66	24.87	2.01	4.76	65.80	0.07	28.25	56.90
	6	11.43	54.35	8.78	0.66	24.25	1.67	4.76	66.05	0.54	28.35	47.46
	7	11.08	54.01	8.80	0.70	24.60	2.01	4.88	64.02	0.81	28.20	56.55
	8	11.59	51.79	8.07	0.74	24.62	1.11	4.47	66.99	3.19	26.33	52.67
6°C	1	11.82	51.67	8.88	0.62	25.76	2.00	4.37	68.30	1.25	27.24	54.60
	2	11.14	52.66	8.92	0.69	22.25	2.00	4.73	64.41	4.35	28.29	56.79
	3	10.47	52.64	8.26	0.96	21.69	2.01	5.03	60.51	5.99	27.47	55.23
	4	11.12	53.46	8.73	0.69	21.31	2.01	4.81	64.30	4.69	28.49	57.08
	5	10.98	53.33	8.91	0.73	25.38	2.00	4.86	63.48	0.67	27.97	58.76
	6	11.45	52.11	8.88	0.67	25.50	2.10	4.55	66.17	1.39	27.45	54.92
	7	12.11	52.30	9.02	0.62	21.17	2.00	4.32	69.98	4.79	28.50	56.99
	8	12.35	52.49	8.99	0.67	24.37	2.00	4.25	71.39	1.14	27.95	55.51
8°C	1	11.72	52.68	9.01	0.70	25.69	1.99	4.50	67.73	0.20	27.82	55.22
	2	11.35	53.96	9.14	0.67	25.60	1.99	4.75	65.62	-0.73	28.49	56.26
	3	11.77	52.93	9.05	0.67	25.43	1.97	4.50	68.05	0.15	28.01	42.11
	4	11.59	53.22	8.90	0.76	25.57	1.50	4.59	66.96	-0.03	27.88	54.99
	5	11.59	53.20	9.07	0.67	25.69	1.97	4.59	67.01	-0.22	28.09	53.38
	6	11.66	52.85	8.87	0.77	25.75	1.90	4.53	67.38	0.10	27.68	54.54
	7	11.38	53.30	10.47	0.63	25.32	1.97	4.68	65.76	-1.10	30.20	60.02
	8	11.82	52.89	9.00	0.66	25.40	1.99	4.47	68.34	0.24	27.93	55.50
10°C	1	11.68	52.55	10.28	0.79	25.14	1.82	4.50	67.51	-0.44	29.70	51.20
	2	10.78	52.29	10.08	0.81	25.46	1.72	4.85	62.34	0.58	29.26	50.51
	3	11.51	52.86	10.15	0.83	25.68	1.73	4.59	66.54	-1.03	29.54	54.00
	4	11.16	52.87	10.04	0.69	25.46	1.83	4.74	64.49	-0.22	29.41	56.77
	5	11.07	53.03	9.98	0.76	24.40	1.93	4.79	64.00	0.77	29.57	54.19
	6	10.99	53.18	9.91	0.82	26.77	1.83	4.84	63.52	-1.67	29.12	53.36
	7	10.90	53.33	9.85	0.69	26.64	1.83	4.89	63.03	-1.42	29.09	56.34
	8	10.38	51.80	9.82	0.71	27.06	1.94	4.99	59.97	0.23	28.42	55.04
12°C	1	11.07	51.19	7.72	0.88	25.35	2.00	4.63	63.96	3.80	25.49	51.23
	2	11.92	50.12	7.52	0.95	24.21	2.01	4.20	68.92	5.28	25.02	49.13
	3	11.27	49.99	7.76	0.79	25.34	1.96	4.43	65.15	4.85	25.10	50.57
	4	11.40	51.55	7.72	0.87	25.33	2.01	4.52	65.87	3.15	25.62	47.96
	5	10.80	51.93	7.71	0.90	25.43	1.87	4.81	62.44	3.23	25.74	54.12
	6	11.28	51.25	7.79	0.80	25.25	2.10	4.55	65.18	3.64	25.63	47.41
	7	11.27	51.27	7.88	0.80	25.65	1.85	4.55	65.14	3.13	25.69	50.30
	8	11.07	52.09	7.88	0.82	26.02	1.96	4.70	64.00	2.12	25.92	50.75

Hatching yolk sac												
		C	H	N	S	O	Weight mg	C/N	% P	%Ash	V cal J.	V E J.ind-1
4°C	1	51.01	7.35	10.50	0.84	23.00	0.02	4.86	60.66	7.30	1580.75	33.72
	2	51.27	7.58	11.19	0.80	23.17	0.02	4.58	64.70	6.00	1650.01	35.26
	3	51.20	7.56	10.01	0.73	23.12	0.02	5.11	57.86	7.39	1643.14	39.16
	4	51.26	7.53	10.20	0.72	22.70	0.03	5.03	58.93	7.59	1652.06	46.26
	5	50.76	7.44	11.04	0.81	22.92	0.02	4.60	63.81	7.04	1612.85	30.38
	6	51.40	7.38	10.68	0.77	22.79	0.03	4.81	61.73	6.98	1598.27	41.82
	7	50.43	7.29	11.27	0.85	22.90	0.02	4.47	65.14	7.27	1562.78	34.38
	8											
6°C	1	50.14	7.20	10.80	0.89	22.81	0.02	4.64	41.61	8.16	16294.75	36.33
	2	51.21	7.42	10.73	0.71	22.76	0.02	4.77	62.00	7.16	1611.32	35.11
	3	51.07	7.37	10.73	0.75	22.57	0.02	4.76	62.01	7.51	1602.76	35.14
	4	50.48	7.29	11.19	0.78	22.42	0.02	4.51	64.67	7.83	1581.76	35.31
	5	49.90	7.31	11.01	0.77	22.24	0.02	4.53	63.65	8.77	1597.44	34.03
	6	51.02	7.72	11.18	0.67	22.22	0.02	4.57	64.59	7.20	1733.83	35.63
	7											
	8											
8°C	1	49.72	7.16	11.37	0.94	24.44	0.03	4.38	65.69	6.37	1453.57	38.91
	2	50.53	7.27	10.96	0.84	24.59	0.02	4.61	63.32	5.81	1481.56	33.29
	3	50.14	7.20	10.80	0.89	24.19	0.03	4.64	62.42	6.78	1477.16	42.59
	4	50.13	7.42	11.36	0.80	24.34	0.02	4.41	65.65	5.95	1545.57	35.65
	5	49.74	7.22	11.33	0.80	24.24	0.02	4.39	65.46	6.67	1480.32	22.25
	6	49.99	7.26	11.25	0.82	23.82	0.03	4.44	65.04	6.86	1512.18	38.96
	7	51.57	7.17	10.68	0.86	23.95	0.03	4.83	61.73	5.77	1476.64	39.43
	8	49.70	7.31	11.63	0.78	23.87	0.02	4.27	67.23	6.71	1524.61	33.80
10°C	1	51.62	7.66	10.77	0.79	23.10	0.03	4.79	62.26	6.05	1681.37	42.82
	2	49.93	7.40	11.80	0.80	23.21	0.03	4.23	68.20	6.87	1584.33	40.00
	3	50.14	7.91	10.97	0.76	23.37	0.04	4.57	63.38	6.86	1752.87	31.46
	4	50.94	8.01	10.15	0.70	23.27	0.03	5.02	58.68	6.93	1790.12	29.25
	5	50.80	7.83	10.13	0.70	23.39	0.02	5.01	58.57	7.15	1724.93	37.96
	6	51.39	7.76	10.98	0.77	23.17	0.02	4.68	63.45	5.93	1712.97	32.40
	7	50.93	7.74	11.36	0.81	23.11	0.02	4.49	65.63	6.05	1708.51	36.22
	8	51.83	7.61	11.38	0.80	23.17		4.55	65.76	5.22	1660.78	
12°C	1	49.46	7.95	10.79	0.74	23.39	0.02	4.58	62.35	7.67	1765.41	34.87
	2	51.75	7.53	10.90	0.85	23.08	0.02	4.75	63.01	5.90	1636.93	31.35
	3	51.94	7.44	10.27	0.88	23.03	0.02	5.06	59.35	6.45	1609.42	31.63
	4	50.13	7.35	12.64	0.81	22.98	0.02	3.97	73.03	6.10	1578.68	26.66
	5	52.32	7.26	10.00	0.74	22.93	0.02	5.23	57.81	6.75	1549.40	26.74
	6	52.51	7.17	10.37	0.67	22.88	0.02	5.06	59.94	6.39	1519.39	28.31
	7	52.70	7.08	10.74	0.60	22.84	0.03	4.91	62.06	6.05	1489.30	43.19
	8	52.89	7.00	11.10	0.60	22.79	0.02	4.76	64.18	5.62	1461.10	28.30

Emergence yolk sac												
		C	H	N	S	O	Weigth mg	C/N	% P	%Ash	Vcal J.	VE J.ind-1
	1	52.55	7.17	10.10	0.67	23.32	0.01	5.21	58.35	6.20	1500.93	12.91
	2	51.77	6.68	10.19	0.80	24.01	0.01	5.08	58.89	6.55	1305.61	11.92
	3	54.44	7.37	8.70	0.62	23.94	0.01	6.26	50.31	4.92	1540.70	16.28
4°C	4	54.33	7.35	9.80	0.73	24.25	0.01	5.55	56.63	3.54	1523.87	21.39
	5	51.92	6.78	9.93	0.75	24.04	0.01	5.23	57.41	6.58	1335.12	12.55
	6	50.87	6.54	10.16	0.82	23.60	0.01	5.01	58.70	8.03	1272.73	8.36
	7	50.68	6.55	10.73	0.86	24.56	0.01	4.72	62.02	6.62	1238.15	14.40
	8	50.66	6.60	9.59	0.83	24.93	0.01	5.28	55.42	7.39	1236.71	11.54
	1	52.34	7.60	10.42	1.06	23.60	0.02	5.02	60.24	4.99	1645.84	35.88
	2	50.71	7.17	10.72	1.27	24.04	0.02	4.73	61.98	6.09	1481.72	24.74
	3	50.38	7.08	11.16	1.30	24.00	0.02	4.52	64.49	6.08	1452.97	25.43
6°C	4	50.96	6.89	11.12	1.30	23.96	0.02	4.58	64.29	5.77	1392.46	21.30
	5	51.35	7.06	10.53	1.09	24.05	0.01	4.88	60.87	5.92	1441.11	18.66
	6	51.32	7.19	10.69	0.98	23.67	0.02	4.80	61.81	6.15	1499.13	29.01
	7	49.80	7.16	11.52	0.96	23.47	0.02	4.32	66.59	7.09	1494.19	28.09
	8	50.23	7.22	11.59	0.97	23.53		4.33	66.98	6.46	1515.89	
	1	50.64	7.20	10.92	0.73	23.59	0.02	7.03	63.13	6.92	1500.44	33.56
	2	49.12	6.85	11.86	0.78	23.78	0.02	7.17	68.56	7.61	1372.48	28.46
	3	47.68	6.87	11.37	0.84	23.15	0.02	6.94	65.70	10.10	1406.33	22.78
8°C	4	49.47	6.92	11.40	0.82	23.59	0.02	7.15	65.86	7.82	1403.18	32.97
	5	47.95	6.72	11.79	0.84			7.14	68.12			
	6	52.32	6.73	10.15	0.84	25.01	0.02	7.77	58.64	4.96	1280.17	29.66
	7	51.92	7.03	10.22	0.80	23.59	0.02	7.39	59.07	6.43	1443.06	29.39
	8	51.92	7.03	10.22	0.80			7.39	59.07			
	1	51.51	7.37	11.45	0.78	23.25	0.02	6.99	66.20	5.63	1575.36	32.14
	2	51.65	6.79	10.87	0.82	23.03	0.01	7.61	62.83	6.85	1384.16	16.61
	3	52.41	7.08	9.80	0.67	23.12	0.01	7.40	56.62	6.93	1476.90	20.68
10°C	4	50.41	7.51	11.75	0.83	23.28	0.02	6.71	67.90	6.23	1621.64	26.59
	5	51.36	7.07	11.22	0.87	23.01	0.02	7.27	64.86	6.49	1481.73	32.30
	6	53.72	7.62	9.36	0.66		0.03	7.05	54.08	5.96	1683.41	
	7											
	8											
	1	50.94	6.19	9.69	0.79	21.96	0.00	5.26	56.00	10.43	1225.21	6.15
	2	52.59	7.07	9.37	0.64	22.88	0.00	5.61	54.18	7.46	1482.00	10.94
	3	56.06	6.80	7.97	0.58	21.66	0.00	7.04	46.04	6.93	1444.27	1.53
12°C	4	54.89	7.23	9.17	0.60	23.44	0.00	5.99	53.00	4.68	1513.62	1.69
	5	54.09	6.70	9.37	0.62	22.57	0.00	5.77	54.18	6.66	1370.78	4.33
	6	54.09	6.70	9.37	0.62	22.96	0.00	5.77	54.18	6.27	1354.01	2.84
	7											
	8											

First food intake yolk sac												
		C	H	N	S	O	poids	C/N	% p	%cendre	V cal	VE
	1.00	51.31	7.12	10.86	0.99	24.65	0.01	7.20	62.78	5.07	1434.34	7.75
	2.00	50.18	7.18	11.19	0.98	25.25	0.00	6.99	64.67	5.23	1425.61	5.23
	3.00	50.37	7.43	11.61	0.93	24.56	0.00	6.78	67.09	5.09	1543.02	7.41
	4.00	50.53	7.36	11.72	0.94	24.69	0.00	6.87	67.76	4.75	1512.18	3.18
	5.00	50.70	7.53	11.60	0.86	24.07	0.00	6.74	67.03	5.25	1593.87	7.92
	6.00	50.00	7.04	11.41	1.06	24.69	0.01	7.10	65.96	5.80	1405.10	10.26
	7.00	50.51	7.08	11.64	1.04	24.79	0.01	7.14	67.30	4.94	1412.53	7.72
4°C	8.00	50.44	7.29	11.35	0.83	25.31	0.00	6.92	65.62	4.78	1459.80	5.94
	1.00	48.60	7.02	11.90	0.98	24.51	0.01	6.93	68.77	6.99	1402.20	10.10
	2.00	47.41	7.06	12.03	0.96	24.14	0.01	6.71	69.54	8.40	1432.50	21.11
	3.00	47.69	7.12	11.97	0.95	24.08	0.02	6.70	69.19	8.19	1454.97	27.64
	4.00	48.92	7.24	11.51	0.93	24.16	0.01	6.76	66.53	7.24	1491.96	13.97
	5.00	47.87	6.84	11.86	1.01	24.11	0.02	7.00	68.57	8.30	1359.73	22.89
	6.00	48.22	7.13	11.82	0.92	24.11	0.02	6.76	68.34	7.80	1456.44	27.38
	7.00	48.48	7.02	12.29	0.94	24.40	0.01	6.91	71.01	6.87	1405.19	15.84
6°C	8.00	48.26	7.11	11.97	0.87	23.68	0.01	6.78	69.21	8.10	1468.05	21.97
	1.00	50.04	6.98	10.83	0.78	24.42	0.01	4.62	62.58	6.95	1390.41	8.81
	2.00	51.37	6.68	9.99	0.84	24.78	0.01	5.14	57.73	6.34	1273.18	7.85
	3.00	49.86	6.73	11.36	0.81	25.50	0.01	4.39	65.68	5.74	1255.83	11.93
	4.00	49.68	7.00	11.11	0.79	25.55	0.01	4.47	64.19	5.88	1347.25	18.05
	5.00	48.74	6.35	10.98	0.99	25.47	0.01	4.44	63.47	7.47	1133.10	10.16
	6.00	50.61	7.27	11.29	0.75	25.27	0.01	4.48	65.28	4.81	1452.59	14.53
	7.00	49.42	6.91	11.18	0.78	25.38	0.02	4.42	64.60	6.34	1321.73	19.87
8°C	8.00	50.27	6.93	10.73	0.77	24.49	0.01	4.68	62.02	6.81	1367.57	15.00
	1.00	51.16	7.12	11.46	0.88	22.72	0.01	4.46	66.23	6.66	1514.11	22.46
	2.00	52.50	7.53	10.75	0.85	23.20	0.01	4.88	62.14	5.18	1632.19	18.82
	3.00	51.46	7.44	10.85	0.84	22.86	0.01	4.74	62.71	6.57	1614.98	23.74
	4.00	52.24	7.58	10.63	0.83	22.58	0.01	4.92	61.42	6.15	1676.08	16.82
	5.00	52.63	7.58	10.85	0.80	22.89	0.01	4.85	62.74	5.24	1664.05	16.03
	6.00	52.17	7.41	11.26	0.85	23.16	0.01	4.63	65.05	5.16	1593.50	13.70
	7.00	51.82	7.43	10.64	0.88	22.54	0.01	4.87	61.52	6.69	1629.10	17.54
10°C	8.00	50.26	7.14	11.21	0.93	23.21		4.48	64.78	7.26	1500.12	
	1.00	46.14	6.64	10.56	0.92	24.20	0.00	6.95	61.04	11.54	1283.31	1.54
	2.00	45.65	6.79	10.87	0.82	23.03	0.00	6.72	62.83	12.85	1381.95	1.66
	3.00	49.41	6.08	9.80	0.67	23.12	0.00	8.13	56.62	10.93	1131.79	1.70
	4.00	49.41	7.51	11.75	0.83	23.28	0.01	6.58	67.90	7.23	1621.27	15.79
	5.00	45.36	7.07	9.22	0.87	23.01	0.00	6.42	53.30	14.49	1479.52	1.92
	6.00	50.75	6.49	11.50	0.80	22.68	0.00	7.82	66.46	7.79	1294.96	3.72
	7.00	45.67	6.65	9.72	0.79	22.47	0.00	6.87	56.17	14.71	1356.18	4.77
12°C	8.00	46.59	6.80	11.94	0.78	22.26	0.00	6.85	69.00	11.64	1419.61	5.65
