# Supplementary material for: Strong Effects of Temperature on the Early Life Stages of a Cold Stenothermal Fish Species, Brown Trout (Salmo trutta L.)
Source: PLoS One. 2016 May 12;11(5):e0155487. doi: 10.1371/journal.pone.0155487 (PMC4865038; doi:10.1371/journal.pone.0155487)
Supplement: Supplementary file 4 [file pone.0155487.s004.docx]

Hatching tissue fry												
		C	H	N	S	O	Weight mg	C/N	% P	%Ash	V cal J.	VE J.ind-1
4°C	1.00	47.61	6.69	11.87	0.92	24.01	0.01	4.01	68.61	8.91	1310.02	11.62
	2.00	47.66	7.03	11.17	0.82	23.73	0.01	4.27	64.54	9.59	1434.82	13.30
	3.00	46.66	6.60	11.76	0.81	23.75	0.01	3.97	67.98	10.42	1287.92	11.42
	4.00	49.78	7.04	11.13	0.81	24.04	0.01	4.47	64.30	7.21	1426.65	12.03
	5.00	49.92	7.22	11.40	0.89	22.92	0.01	0.14	65.87	7.65	2959.90	44.30
	6.00	47.59	6.90	11.49	0.78	23.74	0.01	4.14	66.42	9.51	1389.86	12.23
	7.00	47.26	6.97	11.26	0.73	23.42	0.01	4.20	65.10	10.35	1425.93	12.02
	8.00	47.47	6.61	11.28	0.87	22.49	0.01	4.21	65.19	11.28	1346.04	17.05
6°C	1.00	48.09	7.40	11.29	0.79	22.63	0.01	4.26	65.27	9.80	1608.29	21.66
	2.00	46.51	6.85	11.20	0.83	23.18	0.01	4.15	64.72	11.44	1397.93	10.14
	3.00	46.61	6.86	11.64	0.79	23.28	0.01	4.00	67.29	10.82	1394.93	15.20
	4.00	47.15	6.91	11.50	0.84	23.49	0.02	4.10	66.46	10.12	1405.03	27.40
	5.00	48.30	7.08	11.53	0.75	23.21	0.02	4.19	66.64	9.13	1473.62	23.82
	6.00	48.14	7.10	11.96	0.80	22.80	0.01	4.03	69.12	9.21	1498.94	16.34
	7.00	47.74	7.16	11.57	0.76	22.96	0.02	4.13	66.85	9.82	1513.37	29.51
	8.00	48.68	7.18	11.02	0.81	22.86	0.02	4.42	63.69	9.45	1526.12	25.28
8°C	1.00	51.68	7.58	11.31	0.81	22.82	0.01	4.57	65.37	5.81	1664.24	24.63
	2.00	51.75	7.43	10.71	0.75	22.62	0.01	4.83	61.89	6.74	1620.64	16.61
	3.00	52.34	7.16	10.45	0.81	21.65	0.02	5.01	60.38	7.59	1571.58	26.72
	4.00	50.74	7.04	11.28	0.89	23.06	0.01	4.50	65.18	7.00	1471.07	20.89
	5.00	52.50	7.27	10.86	0.86	21.88	0.02	4.84	62.74	6.63	1600.50	25.93
	6.00	49.44	7.21	11.96	0.94	22.34	0.01	4.13	69.15	8.11	1560.04	20.28
	7.00	52.65	7.43	10.53	0.77	21.99	0.02	5.00	60.88	6.63	1649.60	25.49
	8.00	52.14	7.37	10.50	0.76	22.30	0.02	4.96	60.71	6.92	1615.32	29.56
10°C	1.00	50.55	7.46	11.47	0.85	22.79	0.02	4.41	66.29	6.89	1626.40	27.49
	2.00	51.37	7.46	10.86	0.88	23.17	0.02	4.73	62.78	6.27	1609.33	26.55
	3.00	48.97	7.44	11.64	0.82	23.64	0.01	4.21	67.30	7.50	1580.73	20.94
	4.00	50.10	7.59	10.82	0.82	22.62	0.01	4.63	62.52	8.04	1676.87	15.75
	5.00	49.21	7.42	10.46	0.84	22.75	0.01	4.70	60.46	9.32	1612.82	19.68
	6.00	49.98	7.53	11.46	0.83	23.56	0.02	4.36	66.24	6.64	1615.64	24.23
	7.00	50.20	7.68	10.34	0.76	23.23	0.01	4.86	59.75	7.78	1680.44	20.33
	8.00	51.83	7.98	11.38	0.76	23.02	0.01	4.55	65.76	5.03	1793.62	23.68
12°C	1.00	51.73	7.86	10.87	0.73	22.77	0.01	4.76	62.81	6.06	1760.94	22.63
	2.00	50.63	7.70	10.16	0.78	23.22	0.01	4.99	58.70	7.52	1688.58	16.40
	3.00	50.87	7.74	9.96	0.77	23.23	0.01	5.11	57.57	7.44	1702.40	23.91
	4.00	51.10	7.78	9.76	0.76	23.24	0.01	5.23	56.43	7.35	1716.22	17.98
	5.00	51.34	7.83	9.57	0.75	23.26	0.01	5.37	55.29	7.27	1730.04	20.42
	6.00	49.57	7.87	9.37	0.74	23.27	0.02	5.29	54.15	9.18	1743.12	26.38
	7.00	51.80	7.91	9.17	0.73	23.28	0.01	5.65	53.02	7.10	1757.68	16.46
	8.00	49.04	7.95	8.98	0.72	23.30	0.01	5.46	51.88	10.02	1770.39	20.53

Emergence tissue												
		C	H	N	S	O	Weight mg	C/N	% p	%Ash	V cal J.	VE J.ind-1
4°C	1.00	51.31	7.12	10.86	0.99	24.65	0.02	7.20	62.78	5.07	1434.34	22.09
	2.00	50.18	7.18	11.19	0.98	25.25	0.01	6.99	64.67	5.23	1425.61	23.78
	3.00	50.37	7.43	11.61	0.93	24.56	0.00	6.78	67.09	5.09	1543.02	27.41
	4.00	50.53	7.36	11.72	0.94	24.69	0.01	6.87	67.76	4.75	1512.18	33.76
	5.00	50.70	7.53	11.60	0.86	24.07	0.00	6.74	67.03	5.25	1593.87	27.18
	6.00	50.00	7.04	11.41	1.06	24.69	0.02	7.10	65.96	5.80	1405.10	23.89
	7.00	50.51	7.08	11.64	1.04	24.79	0.01	7.14	67.30	4.94	1412.53	27.72
	8.00	50.44	7.29	11.35	0.83	25.31	0.01	6.92	65.62	4.78	1459.80	33.59
6°C	1.00	50.64	7.20	10.92	0.73	23.59	0.02	7.03	63.13	6.92	1500.44	29.06
	2.00	49.12	6.85	11.86	0.78	23.78	0.02	7.17	68.56	7.61	1372.48	27.08
	3.00	47.68	6.87	11.37	0.84	23.15	0.02	6.94	65.70	10.10	1406.33	22.78
	4.00	49.47	6.92	11.40	0.82	23.59	0.02	7.15	65.86	7.82	1403.18	32.97
	5.00	47.95	6.72	11.79	0.84	23.59	0.02	7.14	68.12	9.12	1334.72	21.62
	6.00	52.32	6.73	10.15	0.84	25.01	0.02	7.77	58.64	4.96	1280.17	24.41
	7.00	51.92	7.03	10.22	0.80	23.59	0.02	7.39	59.07	6.43	1443.06	27.93
	8.00	51.92	7.03	10.22	0.80	23.59	0.02	7.39	59.07	6.44	1443.27	23.38
8°C	1.00	48.60	7.02	11.90	0.98	24.51	0.01	6.93	68.77	6.99	1402.20	10.10
	2.00	47.41	7.06	12.03	0.96	24.14	0.01	6.71	69.54	8.40	1432.50	21.11
	3.00	47.69	7.12	11.97	0.95	24.08	0.02	6.70	69.19	8.19	1454.97	27.64
	4.00	48.92	7.24	11.51	0.93	24.16	0.01	6.76	66.53	7.24	1491.96	13.97
	5.00	47.87	6.84	11.86	1.01	24.11	0.02	7.00	68.57	8.30	1359.73	22.89
	6.00	48.22	7.13	11.82	0.92	24.11	0.02	6.76	68.34	7.80	1456.44	27.38
	7.00	48.48	7.02	12.29	0.94	24.40	0.01	6.91	71.01	6.87	1405.19	15.84
	8.00	48.26	7.11	11.97	0.87	23.68	0.01	6.78	69.21	8.10	1468.05	21.97
10°C	1.00	51.51	7.37	11.45	0.78	23.25	0.02	6.99	66.20	5.63	1575.36	28.99
	2.00	51.65	6.79	10.87	0.82	23.03	0.01	7.61	62.83	6.85	1384.16	16.61
	3.00	50.41	7.08	9.80	0.67	23.12	0.01	7.12	56.62	8.93	1476.16	20.67
	4.00	50.41	7.51	11.75	0.83	23.28	0.02	6.71	67.90	6.23	1621.64	26.43
	5.00	51.36	7.07	11.22	0.87	23.01	0.02	7.27	64.86	6.49	1481.73	32.30
	6.00	49.72	7.62	9.36	0.66	22.69	0.02	6.52	54.08	9.96	1681.94	28.93
	7.00											
	8.00											
12°C	1.00	50.22	7.24	11.69	0.93	24.86	0.01	6.94	67.57	5.07	1461.56	29.16
	2.00	49.84	7.21	12.05	0.89	25.01	0.01	6.92	69.64	5.00	1445.90	27.81
	3.00	50.03	7.22	11.87	0.91	24.93	0.01	6.93	68.60	5.04	1453.73	29.48
	4.00	50.16	7.23	11.75	0.92	24.88	0.01	7.94	67.92	5.06	1458.95	35.60
	5.00	50.09	7.23	11.81	0.91	24.91	0.01	6.93	68.26	5.05	1456.34	28.04
	6.00	49.78	7.21	12.11	0.88	25.03	0.01	7.91	69.98	4.99	1443.29	32.25
	7.00	49.97	7.22	11.93	0.90	24.96	0.01	6.93	68.95	5.02	1451.12	28.92
	8.00	49.90	7.22	11.99	0.90	24.98	0.01	7.92	69.29	5.01	1448.51	35.37

First food intake fry												
		C	H	N	S	O	Weight mg	C/N	% P	%Ash	V cal J.	VE J.ind-1
4°C	1.00	53.08	7.11	9.53	0.75	24.47	0.02	5.57	55.08	5.06	1432.62	29.13
	2.00	49.57	6.62	11.21	0.85	23.72	0.02	4.42	64.78	8.04	1294.98	28.14
	3.00	50.22	6.98	10.88	0.74	24.66	0.02	4.62	62.88	6.52	1377.42	22.27
	4.00	49.19	6.59	10.15	0.75	25.51	0.02	4.85	58.67	7.81	1205.79	24.28
	5.00	49.56	7.12	11.09	0.80	23.71	0.02	4.47	64.09	7.72	1468.20	35.53
	6.00	48.05	6.76	10.80	0.81	24.16	0.02	4.45	62.42	9.42	1325.65	29.61
	7.00	46.40	6.60	11.15	0.82	24.76	0.02	4.16	64.45	10.27	1242.71	23.69
	8.00	45.81	6.70	11.54	0.87	25.35	0.02	3.97	66.70	9.74	1254.52	26.43
6°C	1.00	49.42	6.82	12.19	0.78	24.72	0.03	4.06	70.44	6.08	1321.64	33.61
	2.00	48.14	6.58	11.90	0.82	25.27	0.02	4.05	68.78	7.29	1214.80	29.07
	3.00	49.40	6.98	12.00	0.78	25.03	0.02	4.12	69.36	5.82	1362.25	31.51
	4.00	50.38	7.05	11.72	0.79	24.43	0.02	4.30	67.71	5.64	1411.55	29.78
	5.00	49.08	6.92	12.33	0.76	24.98	0.02	3.98	71.29	5.93	1343.13	32.15
	6.00	49.24	6.98	11.46	0.73	24.47	0.03	4.30	66.23	7.12	1386.59	39.29
	7.00	50.43	7.29	11.00	0.72	23.98	0.03	4.59	63.56	6.59	1512.11	41.23
	8.00	50.14	7.05	11.43	0.72	24.07		4.38	66.09	6.60	1425.20	
8°C	1.00	49.65	7.49	12.04	0.95	22.27	0.02	4.12	69.61	7.61	1660.51	22.25
	2.00	50.78	7.78	11.46	0.91	20.43	0.02	4.43	66.22	8.65	1838.07	24.88
	3.00	48.97	7.33	11.78	0.94	23.03	0.02	4.16	68.11	7.96	1571.41	23.49
	4.00	52.40	7.55	9.59	0.92	20.87	0.02	5.47	55.41	8.68	1742.92	25.29
	5.00	47.30	7.13	11.63	0.83	22.34	0.02	4.07	67.23	10.78	1529.16	29.86
	6.00	49.54	7.40	11.96	0.84	21.85	0.02	4.14	69.13	8.41	1646.40	24.46
	7.00	50.13	7.28	11.61	0.91	23.77	0.02	4.32	67.11	6.29	1524.25	24.62
	8.00	51.89	7.49	11.16	0.86	23.46		4.65	64.53	5.14	1606.90	26.24
10°C	1.00	47.19	6.42	11.95	1.14	25.22	0.02	3.95	69.09	8.08	1171.28	30.06
	2.00	47.23	6.50	11.82	1.09	23.40	0.02	4.00	68.30	9.97	1273.55	38.29
	3.00	48.13	6.77	11.33	0.92	24.98	0.02	4.25	65.46	7.88	1295.65	27.76
	4.00	47.90	6.79	11.29	0.92	25.35	0.02	4.24	65.26	7.75	1286.05	29.98
	5.00	49.83	6.79	11.22	0.93	24.40	0.02	4.44	64.82	6.84	1327.09	29.31
	6.00	47.48	6.89	11.91	0.83	26.41	0.02	3.99	68.86	6.48	1273.85	32.10
	7.00	46.35	6.74	12.70	0.90	24.48	0.02	3.65	73.38	8.84	1304.74	27.59
	8.00	46.88	6.89	12.09	0.85	26.45		3.88	69.86	6.85	1269.44	
12°C	1.00	49.17	6.96	11.76	0.79	23.17	0.02	4.18	67.98	8.15	1435.46	27.42
	2.00	50.19	7.33	10.57	0.79	21.66	0.02	4.75	61.10	9.47	1627.82	36.30
	3.00	51.24	7.16	9.72	0.72	22.49	0.02	5.27	56.15	8.67	1534.01	37.58
	4.00	46.93	6.14	11.51	0.82	22.67	0.02	4.08	66.55	11.92	1175.64	24.69
	5.00	49.54	7.11	10.96	0.76	23.58	0.03	4.52	63.33	8.06	1470.58	41.03
	6.00	49.28	6.94	11.40	0.76	22.79	0.02	4.32	65.88	8.83	1444.11	25.42
	7.00											
	8.00											
